# Aortic Stenosis Hyalectan Remodeling Revealed by Proteomics and Glycoproteomics

**DOI:** 10.1161/ATVBAHA.126.323867

**Published:** 2026-07-16

**Authors:** Siqi Yin, Ursula Mayr, Javier Barallobre-Barreiro, Elisa Duregotti, Anna K. Barton, Rong Bing, Dyana Markose, Xiaoke Yin, Padmini Sarathchandra, Bhawana Singh, Wen-Yu Lin, Marika Fava, Lukas E. Schmidt, Ferheen Baig, Ajay M. Shah, Konstantinos Theofilatos, Najma Latif, Christian Hengstenberg, Tamás Radovits, Béla Merkely, Neil C. Henderson, Marc R. Dweck, Manuel Mayr

**Affiliations:** 1 National Heart and Lung Institute, Faculty of Medicine, Imperial College London, United Kingdom (S.Y., U.M., J.B.-B., E.D., X.Y., P.S., B.S., N.L., M.M.).; 2 School of Cardiovascular and Metabolic Medicine & Sciences, Faculty of Life Sciences & Medicine, King’s College London, British Heart Foundation Centre, London, United Kingdom (M.F., F.B., A.M.S., K.T.).; 3 British Heart Foundation Centre for Cardiovascular Science, College of Medicine and Veterinary Medicine, University of Edinburgh, United Kingdom (A.K.B., R.B., M.R.D.).; 4 Centre for Inflammation Research, Institute for Regeneration and Repair, College of Medicine and Veterinary Medicine, University of Edinburgh, United Kingdom (D.M., N.C.H.).; 5 Division of Cardiology, Department of Internal Medicine, Tri-Service General Hospital, National Defense Medical Center, Taipei, Taiwan (W.-Y.L.).; 6 Division of Cardiology, Department of Internal Medicine II, Medical University of Vienna, Austria (L.E.S., C.H., M.M.).; 7 Heart and Vascular Center, Department of Cardiology, Semmelweis University, Budapest, Hungary (T.R., B.M.).

**Keywords:** aortic valve, extracellular matrix, osteoblasts, proteoglycans, proteomics

## Abstract

**BACKGROUND:**

Calcific aortic valve (AV) disease (CAVD) is recognized as an active pathological process involving extracellular matrix remodeling. This study investigates extracellular matrix remodeling through proteomic analysis and a novel mouse model of aortic stenosis.

**METHODS:**

Proteomic and glycoproteomic analyses were conducted on AV leaflets from heart transplant donors (n=29) and patients with CAVD (n=17). Each CAVD sample was subdivided into noncalcified and calcified regions. To investigate the functional impact of extracellular matrix remodeling on aortic stenosis, we crossed apolipoprotein E-deficient mice (*ApoE*^*−/−*^) with mice lacking the catalytic domain of ADAMTS5 (*Adamts5*^*Δcat*^) to generate a mouse model combining hyalectan accumulation with hypercholesterolemia.

**RESULTS:**

Proteomic and glycoproteomic analyses revealed hyalectan accumulation in CAVD compared with control valves. Versican predominated in noncalcified regions, while aggrecan was enriched in calcified regions. The shift in hyalectan composition correlated with changes in AV pressure gradient, elevated osteoblast-like cell markers, and inflammatory proteins, most notably pentraxin 3. Both versican and aggrecan are characterized by their ability to bind hyaluronan and serve as substrates of ADAMTS5. In *Adamts5*^*Δcat*^*/ApoE*^*−*^^*/−*^ mice, hyalectan accumulation was associated with narrowed aortic cusp separation and increased post-AV velocity. Proteomic analysis of AVs from *Adamts5*^*Δcat*^*/ApoE*^*−*^^*/−*^ mice revealed elevated versican, aggrecan, and pentraxin 3, recapitulating key features of human CAVD. Single-cell RNA sequencing and in vitro experiments linked versican to activated valve interstitial cells, while aggrecan colocalized with calcification markers in osteoblast-like cells. Pentraxin 3 was bound to hyaluronan and accumulated in calcified AVs. ADAMTS5 deficiency was sufficient to cause intact versican accumulation and promote valve interstitial cell activation, accompanied by increased expression of osteopontin. Osteopontin is a ligand of CD44, a principle hyaluronan receptor.

**CONCLUSIONS:**

This study highlights hyaluronan remodeling during aortic stenosis pathogenesis. A shift from versican to aggrecan in human CAVD correlates with changes in AV pressure gradient. In *Adamts5*^*Δcat*^*/ApoE*^*−*^^*/−*^ mice, impaired hyalectan catabolism promotes aortic stenosis.

**What Are the Clinical Implications?**

In the first glycoproteomic comparison of human aortic valves (AVs), we identified a shift from versican to aggrecan in calcific AV disease, consistent with cartilage-like matrix remodelling associated with endochondral ossification. This shift is linked to the clinical severity of calcific AV disease in patients. Additionally, scRNA-seq revealed that *Vcan* and *Acan* are expressed by distinct valve interstitial cell subtypes, with *Acan* linked to cells expressing osteogenic markers. Both hyalectans are substrates of ADAMTS5 (a disintegrin and metalloproteinase with thrombospondin motifs 5). Loss of ADAMTS5 activity combined with hypercholesterolemia induces AV dysfunction in mice, supporting hyalectan accumulation as a causal contributor to disease progression rather than a secondary consequence. Increased hyaluronan-associated remodeling and pentraxin 3 deposition connect inflammatory signaling with structural AV deterioration. Therefore, targeting hyalectan- and hyaluronan-related matrix remodeling represents a potential therapeutic strategy for calcific AV disease.

Calcific aortic valve (AV) disease (CAVD) is common in older adults^1^ and ranges from AV sclerosis^2^ to aortic stenosis (AS).^3,4^ Risk factors include aging, hypertension, hypercholesterolemia, male sex, and genetic factors.^3,4^ Most patients are diagnosed only after symptom onset,^5^ and treatments are limited to surgical or transcatheter AV replacement. Although hypercholesterolemia is a known risk factor, cholesterol-lowering therapies have not been effective in treating CAVD, highlighting the need for alternative therapeutic targets, including mechanisms that reduce lipoprotein retention in AVs rather than solely reducing circulating cholesterol levels.^6^

Proteoglycans are key extracellular matrix (ECM) components with glycosaminoglycan chains.^7^ Small leucine-rich proteoglycans (SLRPs) and hyalectans can bind lipoproteins,^8^ thereby contributing to lipoprotein retention in atherosclerosis.^9^ Hyalectans are large aggregating proteoglycans that contribute to tissue hydration through their negatively charged glycosaminoglycan chains. Aggrecan is predominantly found in cartilage,^10^ whereas versican is the most abundant hyalectan in vascular and valve tissue.^11,12^ *ACAN* was recently implicated in AS risk through integrated genetic and transcriptome-wide association analyses, with AV-enriched expression and evidence of colocalization between signals from genome-wide association studies and AV eQTLs.^13^ However, the underlying mechanism remains poorly understood.

ADAMTS (a disintegrin and metalloproteinase with thrombospondin motifs) proteases cleave versican and aggrecan.^14^ Loss of ADAMTS5 activity in *Adamts5*^*Δcat*^ mice causes verscian accumulation and myxomatous AVs,^15,16^ and ADAMTS5 silencing promotes the osteoblast-like transition of human valve interstitial cells (VICs) in vitro.^17^ However, the contribution of hyalectans to human CAVD remains unclear.

In this study, we characterized the ECM composition of AV leaflets from patients with CAVD using proteomic and glycoproteomic analyses. To examine whether hyalectan accumulation contributes to AS pathogenesis, we crossed apolipoprotein E-deficient mice (*ApoE*^−/−^) with *Adamts5*^*Δcat*^ mice, enabling assessment of the combined effects of hypercholesterolemia and impaired ECM remodeling in AV disease.

## Methods

Proteomics and glycoproteomics data from human AVs have been deposited in the ProteomeXchange Consortium via the PRIDE partner repository under accession numbers PXD078618 and PXD078686, respectively. Mouse AV proteomics data have also been deposited in PRIDE under accession number PXD078502. The authors declare that all other supporting data are available within the article. An expanded Materials and Methods section is available in Supplemental Methods.

### Human AV Tissue Collection

Control AVs (n=29) were obtained from heart failure patients undergoing cardiac transplantation at Semmelweis University, Budapest, Hungary (ETT TUKEB 7891/2012/EKU [119/PI/12]; King’s College London REC LRS-17/18–5080). Control AVs were free of calcification, stenosis, and regurgitation. AVs from patients undergoing AV replacement for severe AS (n=17) were obtained from the University of Edinburgh, United Kingdom (REC 16/SS/0027). AS leaflets were segmented into macroscopically noncalcified and calcified regions. In total, 63 samples were analyzed by proteomics. Written informed consent was obtained from all patients.

### Mouse Experiments

All animal procedures were performed by authorized researchers at the School of Cardiovascular and Metabolic Medicine & Sciences, King’s College London, in accordance with the UK Animals (Scientific Procedures) Act 1986. *Adamts5*^*∆cat*^ mice, which lack the catalytic domain of Adamts5 (JAX stock 005771, B6.129P2-Adamts5^tm1Dgen/J^), and *Adamts5*^*+/+*^ wild type (WT) littermates on a C57BL/6J background were used. To generate hyperlipidemic mice lacking ADAMTS5 activity, *ApoE*^*−/−*^ mice (JAX stock 002052, B6.129P2-Apoe^tm1Unc/J^) were crossed with heterozygous *Adamts5*^*Δcat*^^*/+*^ mice. The resulting double-knockout mice were referred to as *Adamts5*^*Δcat*^*/ApoE*^*−/−*^ and abbreviated as *TS5*^*Δcat*^*/ApoE*^*−/−*^ in figures. Because bicuspid AV (BAV) is a major risk factor for AS and CAVD in humans, animals with BAV were excluded from subsequent analyses due to low numbers and potential confounding.

### ECM Protein Extraction

Human AV proteins were extracted using a modified version of our previously published 3-step extraction protocol.^18,19^ Because AVs have limited cellular content, a simplified 2-step extraction was used. Briefly, tissues were sequentially incubated in 0.5 mol/L NaCl buffer for 2 hours to release loosely attached extracellular and cellular proteins, followed by incubation with 4M guanidine hydrochloride (GuHCl) buffer for 48 hours to solubilize core structural ECM proteins. Mouse AVs were extracted using 4 mol/L GuHCl buffer for 48 hours only. The NaCl and GuHCl extracts were enzymatically deglycosylated and digested before proteomic analysis.

### Human ECM Proteomics Analysis

For discovery proteomics, digested peptides from 126 protein extracts generated by NaCl and GuHCl extraction were separated using a nanoflow liquid chromatography (LC) system and analyzed by data-dependent acquisition on a Q Exactive HF Hybrid mass spectrometer (Thermo Fisher Scientific). Raw files were searched against a human database using Proteome Discoverer software (version 2.3.0.523, Thermo Scientific) with Mascot (version 2.6.0, Matrix Science). Precursor and fragment ion mass tolerances were set at 10 ppm and 20 mmu, respectively. Trypsin was specified as the digestion enzyme, allowing up to 2 missed cleavages. Cysteine carbamidomethylation was set as a static modification, whereas lysine, methionine, and proline oxidation and asparagine deamidation in the presence of H_2_^18^O were set as variable modifications. Peptide and protein identifications were filtered at 1% FDR, with at least 2 unique peptides required per protein. Data were normalized to total ion intensity to account for intersample variation.

### Glycoproteomic Analysis

Glycopeptide enrichment and glycoproteomics were performed using an adapted protocol.^20^ Briefly, 35 µg of protein per sample was precipitated, resuspended in urea buffer, reduced, alkylated, reprecipitated, and digested with trypsin. Peptides were labeled with TMTpro 16plex (Thermo Fisher Scientific), enriched for glycopeptides by zwitterionic hydrophilic interaction LC, and fractionated into 16 fractions.

Enriched glycopeptides were separated on a Vanquish Neo nanoflow LC system (Thermo Fisher Scientific) using a C18 column and analyzed on an Orbitrap Ascend mass spectrometer. An acquistion method alternating higher-energy collisional dissociation and electron transfer dissociation with higher-energy collisional dissociation supplemental activation (HCD-alt-EThcD) was applied. Glycopeptides were identified using Byonic software (version 2.9.30, Proteome Metrics) and quantified using TMT reporter ions. Data were normalized to the total peptide amount to account for variation in sample abundance and scaled to an internal pooled sample.

### Echocardiography

Transthoracic echocardiography was performed using standard protocols to assess cardiac and AV function and morphometry with a Vevo 2100 system (VisualSonics). B-mode images were acquired in parasternal views to assess cardiac function, and M-mode was used to measure left ventricular wall thickness and aortic root cusp separation. Pulsed-wave Doppler was used to measure aortic velocity time integral, velocity, and gradient. AS/aortic regurgitation (AR) phenotypes were assessed using a combination of cusp separation, transvalvular blood flow velocity, and color Doppler.

### Single-Cell RNA Sequencing Analysis

Publicly available single-cell RNA sequencing data sets of AVs from hypercholesterolemic mice^21^ were obtained from GEO under accession code GSE180278 using the Seurat v5 tool kit in R version 4.2.3. Preprocessed and normalized gene expression matrices provided by the original authors were used directly for downstream analysis. Cell-cell communication patterns were inferred from the integrated single-cell RNA sequencing data set using CellChat v1.5.0.^22^

### Statistical Analysis

Statistical information for individual experiments is provided in the figure legends. Data are represented as mean±SEM. Two-group comparisons were performed using an unpaired Student *t* test, and multiple-group comparisons involving 1 variable were performed using 1-way ANOVA with Tukey post hoc test. Normality was assessed using the Shapiro-Wilk test before Student *t* tests and ANOVA. Statistical analyses were performed using GraphPad Prism version 9.0 for Mac (GraphPad Software, San Diego, CA). For human samples, statistical comparisons were performed using the limma package^23^ for CAVD versus control comparisons, with eBayes moderation and adjustment for age and sex. Paired *t* tests were used for comparisons between noncalcified and calcified regions. Statistical significance was defined as Benjamini-Hochberg adjusted *P*<0.05. The *lm* function in R was used to perform multivariable relative risk regression analysis, evaluating the association between ECM protein changes and AV pressure gradients while adjusting related covariates.

Proteins from NaCl or GuHCl extracts were filtered for ECM-related proteins using Matrisome annotations^24^ plus selected key proteins listed in Table S1, consistent with previous studies.^18,19^ The data set was further refined to retain proteins with <30% missing values for proteomics, <35% for glycoproteomics, or >90% missingness in 1 phenotype and <10% in another. Missing values were imputed using KNN-Impute (k=3) in the former case and as 0 in the latter. Relative protein quantities were log2-transformed. Glycoproteomics data filtering is described in the Supplemental Methods. Visualizations were generated in R using packages including ggplot2 and ComplexHeatmap. PCA was performed using scikit-learn, and enrichment analysis was performed using DAVID Bioinformatics Resources^25^ and presented as dot plots generated with ggplot2.

## Results

### Comprehensive ECM Analysis of Human AVs

**Figure 1.**
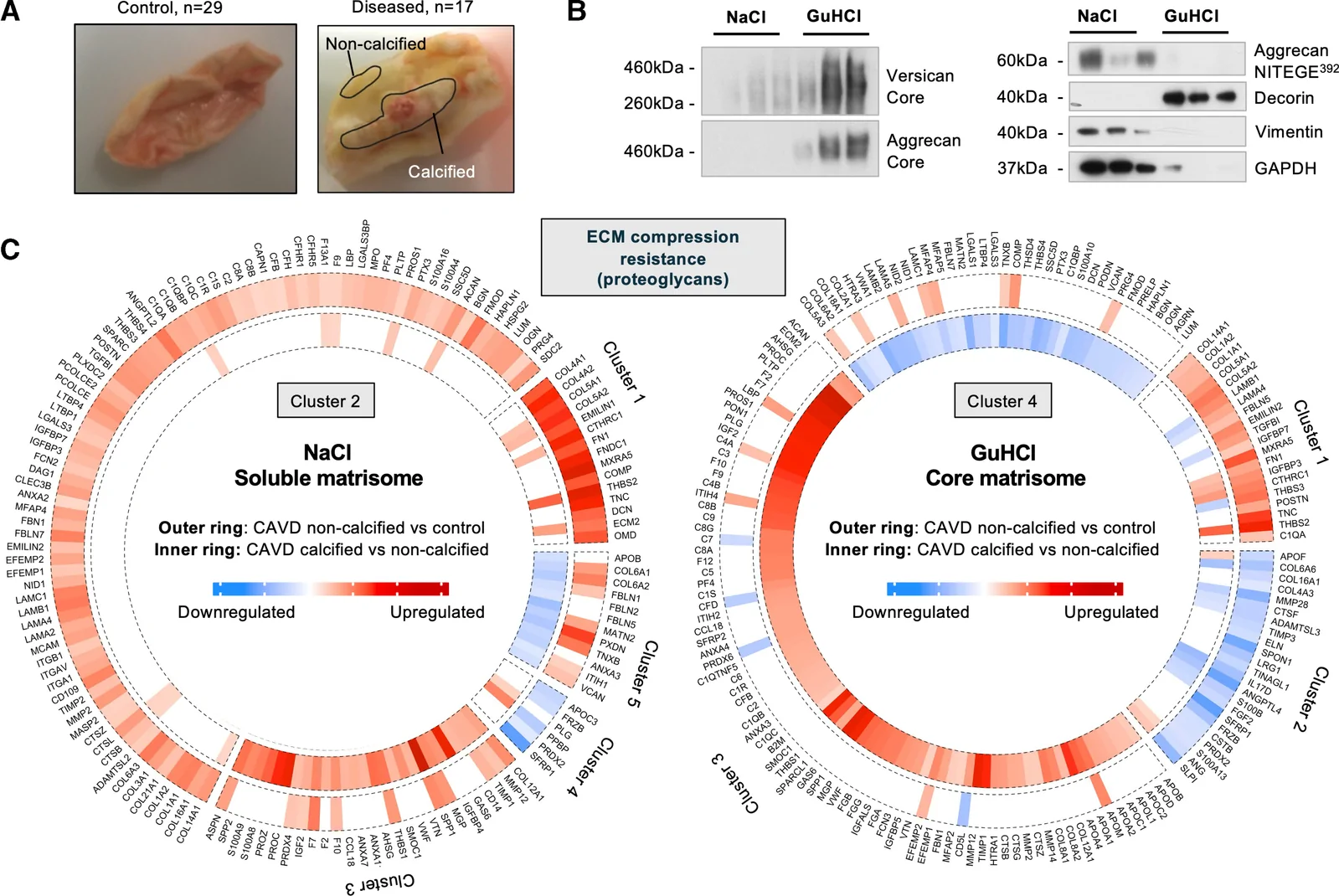
**Proteomics of extracellular matrix (ECM) and ECM-associated proteins in human aortic valves (AVs). A**, Representative images illustrating the analyzed sample groups. **B**, Immunoblots validate the efficiency of the ECM extraction protocol. Intracellular proteins (such as GAPDH and vimentin), as well as ECM cleavage products like the neoepitope NITEGE^392^ of aggrecan (generated by ADAMTS [a disintegrin and metalloproteinase with thrombospondin motifs]-specific cleavage), are detected in the NaCl fraction, while decorin, versican, and the aggrecan core protein are predominantly found in the GuHCl fraction. **C**, The circular heatmap shows hierarchical clustering of significantly altered ECM proteins in the soluble (**left**, NaCl extract) and core (**right**, GuHCl extract) matrisome fractions. The outer ring compares noncalcified calcific aortic valve disease (CAVD) samples (n=17) to controls (n=29), while the inner ring compares calcified to noncalcified CAVD samples (n=17). Red and blue indicate up- and downregulated protein changes relative to median levels, respectively (see color scale). For CAVD noncalcified vs control, statistical significance was calculated using the limma package with the eBayes algorithm, adjusted for age and sex (*P*<0.05; Benjamini-Hochberg correction). Proteins with fold changes (FC) >1.5 or <0.67 with adjusted *P*<0.05 were considered significant. For CAVD calcified vs noncalcified, significance was assessed using Student paired *t* test (*P*<0.05, Benjamini-Hochberg correction), with proteins showing fold changes >1.5. Full protein names are provided in Table S1.

Control AV leaflets were obtained from patients with heart failure without AV disease, and diseased AV leaflets from patients undergoing AV replacement surgeries for severe AS. All samples were fresh-frozen, as we have recently shown^26^ that autopsy samples can yield quantitatively inaccurate results in proteomic analyses due to postmortem protein degradation. Clinical characteristics are provided in Table S2. CAVD samples were dissected into noncalcified (area without calcification nodules) and calcified regions (center of the calcification nodules) within the same valve leaflet (Figure 1A). A total of 63 samples from 46 patients (n=29 control, n=17 CAVD noncalcified, n=17 CAVD calcified) underwent 2-step extraction to obtain soluble (NaCl extracts) and core (GuHCl extracts) matrisome fractions. The resulting 126 protein extracts were analyzed by LC-tandem mass spectrometry (LC-MS/MS).

Extraction efficiency was assessed by immunoblotting (Figure 1B). Hyalectans, including full-length versican and aggrecan, and SLRPs like decorin, were primarily detected in the core matrisome (GuHCl extracts). In contrast, ECM protein fragments, including the ADAMTS-specific aggrecan fragment (neoepitope NITEGE^392^), were observed predominantly in the soluble matrisome (Figure 1B). In AVs, the NaCl extraction was also sufficient to solubilize the bulk of cellular proteins. Intracellular proteins such as GAPDH and vimentin were primarily identified in the NaCl extracts.

LC-MS/MS analysis (Figure S1A) consistently quantified 350 soluble matrisome proteins (Table S3) and 372 core matrisome proteins (Table S4). Principal component analysis (Figure S1B) revealed clear separation of control and noncalcified regions of diseased samples in the soluble matrisome and clear distinction between calcified and noncalcified CAVD regions in the core matrisome (Figure S1C). These findings suggest that the soluble matrisome better distinguishes control from diseased AVs, whereas the core matrisome better captures regional differences between noncalcified and calcified regions within CAVD.

### Discovery ECM Proteomics of Human AVs

Changes in ECM and ECM-associated proteins in the soluble and core matrisome are illustrated in the circular heatmaps in Figure 1C for noncalcified regions versus control AVs and for paired noncalcified and calcified regions from the same AVs. Comparisons between calcified and noncalcified regions were paired, whereas comparisons between control and CAVD samples were adjusted for age and sex. Consistent with the PCA analysis, differences between noncalcified regions and control AVs were more pronounced in the soluble matrisome (outer ring, Figure 1C), whereas differences between noncalcified and calcified CAVD regions were better captured in the core matrisome (inner ring, Figure 1C).

Pathway enrichment analysis using DAVID Bioinformatics Resources^25^ (Figures S1B and S2) identified key pathways altered in CAVD. In the soluble matrisome, proteins upregulated in noncalcified regions compared with control AVs were primarily associated with complement and coagulation (*q*=1.89×10^−17^, cluster 2), cell adhesion (*q*=5.68×10^−12^, clusters 1 and 2), and ECM compression resistance (proteoglycans; *q*=1.37×10^−11^, cluster 2; Figure S1B). In the core matrisome, complement and coagulation pathways were upregulated in calcified versus noncalcified regions (*q*=9.30×10^−43^, cluster 3; Figure S2), along with proteolysis pathways involving cathepsins and collagen/elastin degradation (*q*=5.70×10^−14^). Notably, ECM compression resistance (proteoglycans) was the most downregulated pathway in calcified regions (*q*=3.28×10^−20^, cluster 4; Figure S1C).

Identification of the ECM compression resistance pathway in both extracts (Figure S1B and S1C) highlights the potential role of proteoglycans in preserving AV integrity and mechanical function. Among proteoglycans, hyalectans are large, aggregating proteoglycans that mediate resistance to compression forces through extensive glycosylation, interactions with hyaluronan (HA), and water retention by their glycosaminoglycan chains.

### Glycoproteomics of Human AVs

**Figure 2.**
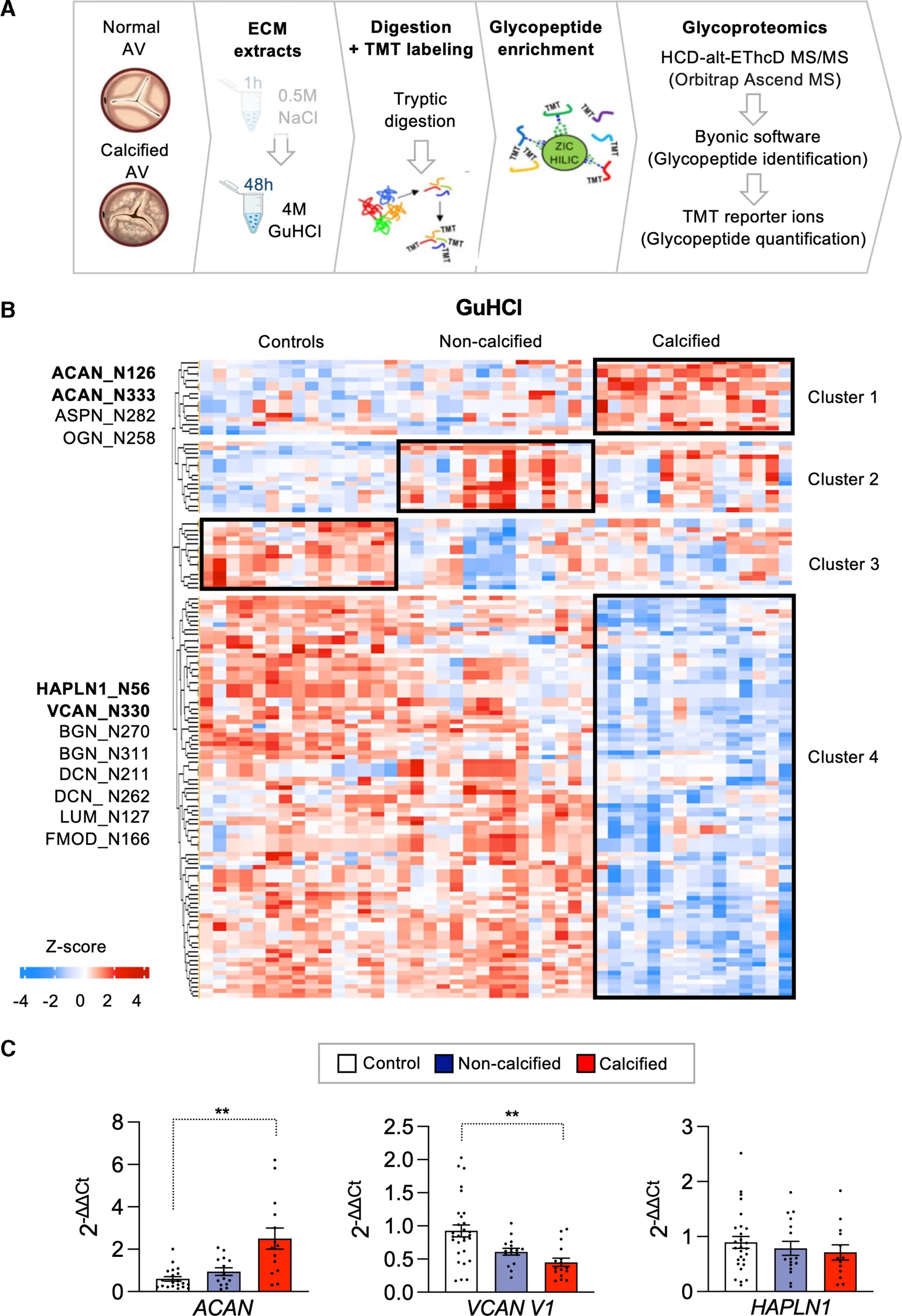
**Glycoproteomic analysis of calcific aortic valve disease (CAVD). A**, The glycoproteomics workflow employed advanced mass spectrometry fragmentation techniques for glycopeptide analysis: HCD-alt-EThcD alternates between Higher-energy Collisional Dissociation (HCD) and Electron Transfer/Higher-energy Collisional Dissociation (EThcD). HCD provides detailed peptide sequencing; EThcD preserves glycosidic bonds, revealing glycan masses and glycosylation sites. **B**, Heatmap of significantly altered proteoglycan glycopeptides in the core matrisome. Glycopeptides from glycosites with >3 glycoforms were labeled. Detailed information on glycopeptides and glycoform information can be found in Tables S5 and S6. Significance was determined using the limma package (eBayes adjustment; *P*<0.05, Benjamini-Hochberg correction; fold changes [FC] >1.5) and Student paired *t* test for noncalcified vs calcified regions in CAVD (*P*<0.05, Benjamini-Hochberg correction; FC >1.5). **C**, Gene expression of versican (*VCAN*), aggrecan (*ACAN*), hyaluronan and proteoglycan link protein 1 (*HAPLN1*) in control, noncalcified, and calcified CAVD. Significance was calculated using the limma package for CAVD vs controls and paired Student *t* test within CAVD groups (**P<0.01). Data are shown as mean±SEM.

Given that glycosylation is a key feature of hyalectans and other proteoglycans, we applied a glycoproteomics workflow for glycopeptide enrichment with tandem mass tag labeling for multiplexed quantification across samples. To improve glycopeptide characterization and glycosylation-site assignment, we used HCD-alt-EThcD fragmentation on an Orbitrap Ascend mass spectrometer. We identified 3229 unique glycopeptides from 274 glycoproteins in core matrisome extracts, with approximately one-quarter derived from proteoglycans (Figure 2A; Table S5).

Focusing on N-linked glycopeptides containing canonical N-glycosylation consensus motifs, we identified 141 N-linked glycoforms from 12 proteoglycans that differed significantly across groups. Glycoproteomic analysis identified 4 distinct ECM-associated clusters that segregated by disease status (Figure 2B; Table S6). Calcified regions were characterized by an aggrecan-dominant proteoglycan profile (cluster 1), whereas noncalcified regions within diseased valves showed enrichment of SLRPs (cluster 2). Control valves showed a higher abundance of matrix-organizing proteoglycans, including perlecan (HSPG2) and biglycan (cluster 3), which declined in diseased tissue. Finally, versican, HAPLN1 (hyaluronan and proteoglycan link protein 1), and multiple SLRPs were markedly reduced in calcified regions compared with both control and noncalcified tissue (cluster 4), consistent with a compositional shift from a versican-rich to an aggrecan-enriched ECM in calcified tissue.

Among the proteoglycan changes observed, the divergence between versican and aggrecan was particularly notable. Although both are hyalectans, they displayed distinct patterns during disease progression. Our extraction protocol is optimized for the recovery of large aggregating proteoglycans, thereby improving their representation compared with conventional proteomic workflows. Notably, the observed shift from versican toward aggrecan parallels the transition toward a cartilage-like ECM during endochondral ossification, which precedes matrix mineralization. Whether similar regulatory mechanisms contribute to CAVD remains to be determined.

To investigate this further, we performed qPCR analysis. Consistent with the proteomics data, aggrecan expression was higher in calcified AV regions, whereas *VCAN* expression was higher in noncalcified regions (Figure 2C). *HAPLN1* transcript levels were unchanged despite reduced protein abundance in calcified areas. Together, reduced versican and HAPLN1 protein abundance with increased aggrecan expression indicate pathological ECM remodeling during calcification. qPCR analysis showed reduced *ADAMTS1* and *ADAMTS5* expression in noncalcified regions (Figure S3A). In contrast, neoepitope staining for ADAMTS-generated versican (DPEAAE, V1, the predominant isoform) and aggrecan (NITEGE) fragments revealed region-specific cleavage patterns, with versican fragments more abundant in noncalcified areas and aggrecan fragments enriched in calcified regions (Figure S3B). Despite lower *ADAMTS* transcript levels in noncalcified tissue, cleavage fragments remained detectable, consistent with previous findings^27^ indicating that neoepitope abundance depends not only on enzyme expression but also on substrate availability. Higher *VCAN* expression in noncalcified regions and higher *ACAN* expression in calcified regions likely contribute to these distribution patterns (Figure 2C). Together, the transcript, neoepitope, and substrate abundance data provide context for interpreting proteoglycan turnover across CAVD progression.

### Hyalectan Remodeling Correlates With AV Pressure Gradients

**Figure 3.**
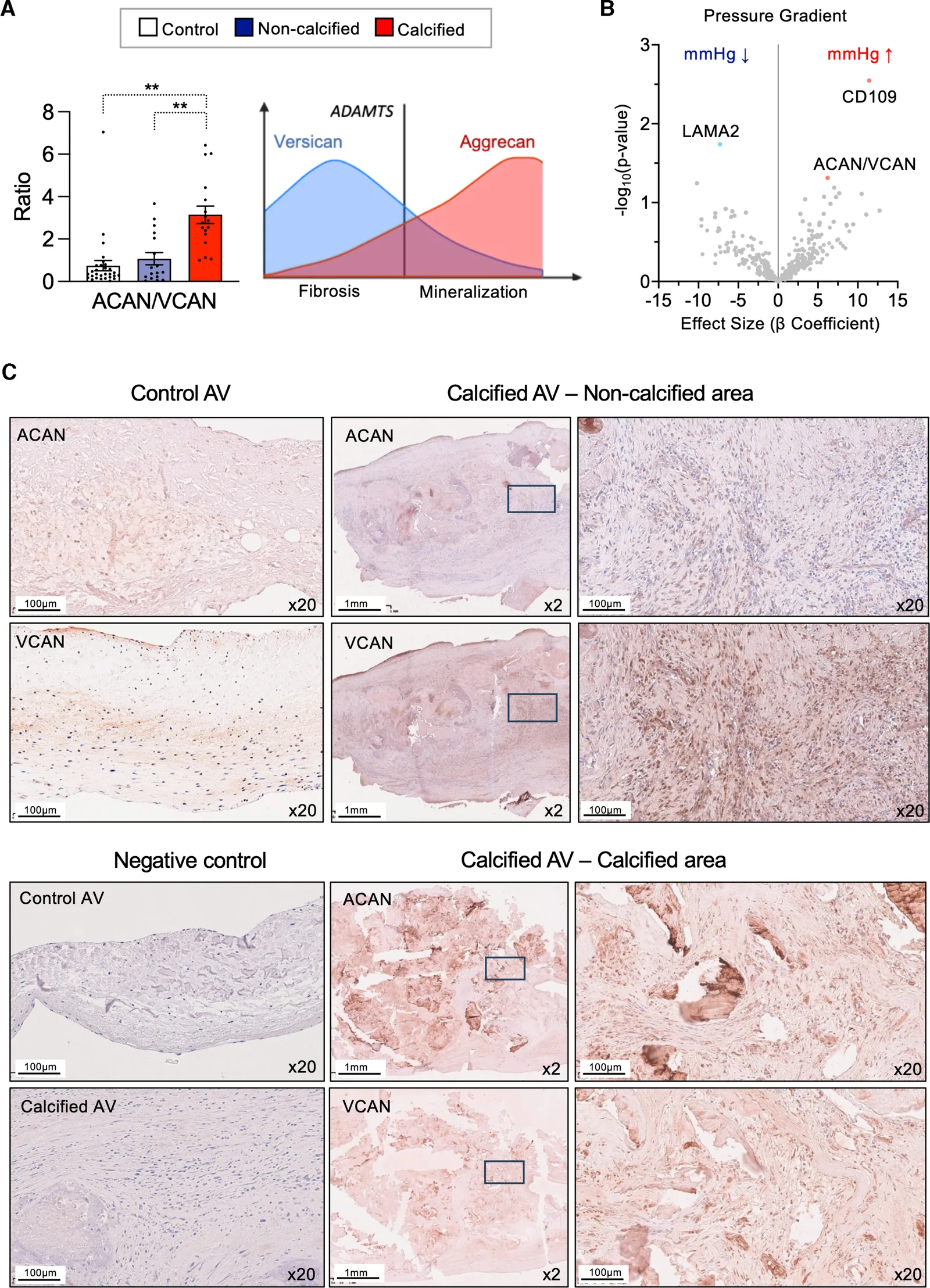
**Versican (VCAN)-to-aggrecan (ACAN) transition during calcification development in human aortic valves (AVs). A**, Scaled ACAN/VCAN ratios in control AVs, noncalcified and calcified calcific aortic valve disease (CAVD) regions, determined by proteomics analysis Statistical significance was calculated using the limma package (age and sex adjustment and Benjamini Hochberg correction) or paired Student *t* test for noncalcified vs calcified regions (**P<0.01; Benjamini-Hochberg correction). Data are shown as mean±SEM. **B**, Volcano plot showing a significant positive association between the ACAN/VCAN ratio and AV pressure gradient. Ratios were based on scaled protein abundances from calcified AV regions. Multivariable relative risk regression analysis was performed after adjustment for age and bicuspid valve covariates to examine the association between extracellular matrix protein changes and AV pressure gradients. **C**, Immunoperoxidase images showing staining for VCAN (noncleaved) and ACAN (noncleaved) in human control and calcified AVs. All sections were counterstained with hematoxylin to visualize nuclei.

To validate the discovery proteomics findings, we developed a targeted parallel reaction monitoring (PRM) method to quantify 118 ECM proteins in the core matrisome (Figure S3C; Table S7). PRM showed strong correlations with untargeted proteomics (Spearman correlation>0.9; Figure S3D), supporting the reliability of the discovery data set. To quantify hyalectan remodeling in CAVD, we calculated the scaled aggrecan/versican abundance ratio as a measure of their relative balance. The aggrecan/versican ratio was highest in calcified regions of diseased AVs (Figure 3A). To assess clinical relevance, we examined the association between AV pressure gradient, a marker of AS severity, and hyalectan protein abundance within diseased AVs using regression models adjusted for age and BAV status. Although individual aggrecan and versican levels were not significantly associated with pressure gradient, the aggrecan/versican ratio showed a significant positive association with pressure gradient (Figure 3B). Immunoperoxidase staining localized versican to the proteoglycan-rich spongiosa of control AV leaflets, with increased staining in noncalcified regions of calcified AVs (Figure 3C). In contrast, aggrecan staining was enhanced in the calcified region, consistent with the proteomics data. These staining patterns support stage-specific accumulation of aggrecan and versican during CAVD progression (Figure 3C).

### VIC Phenotype Changes and Hyalectan Expression

**Figure 4.**
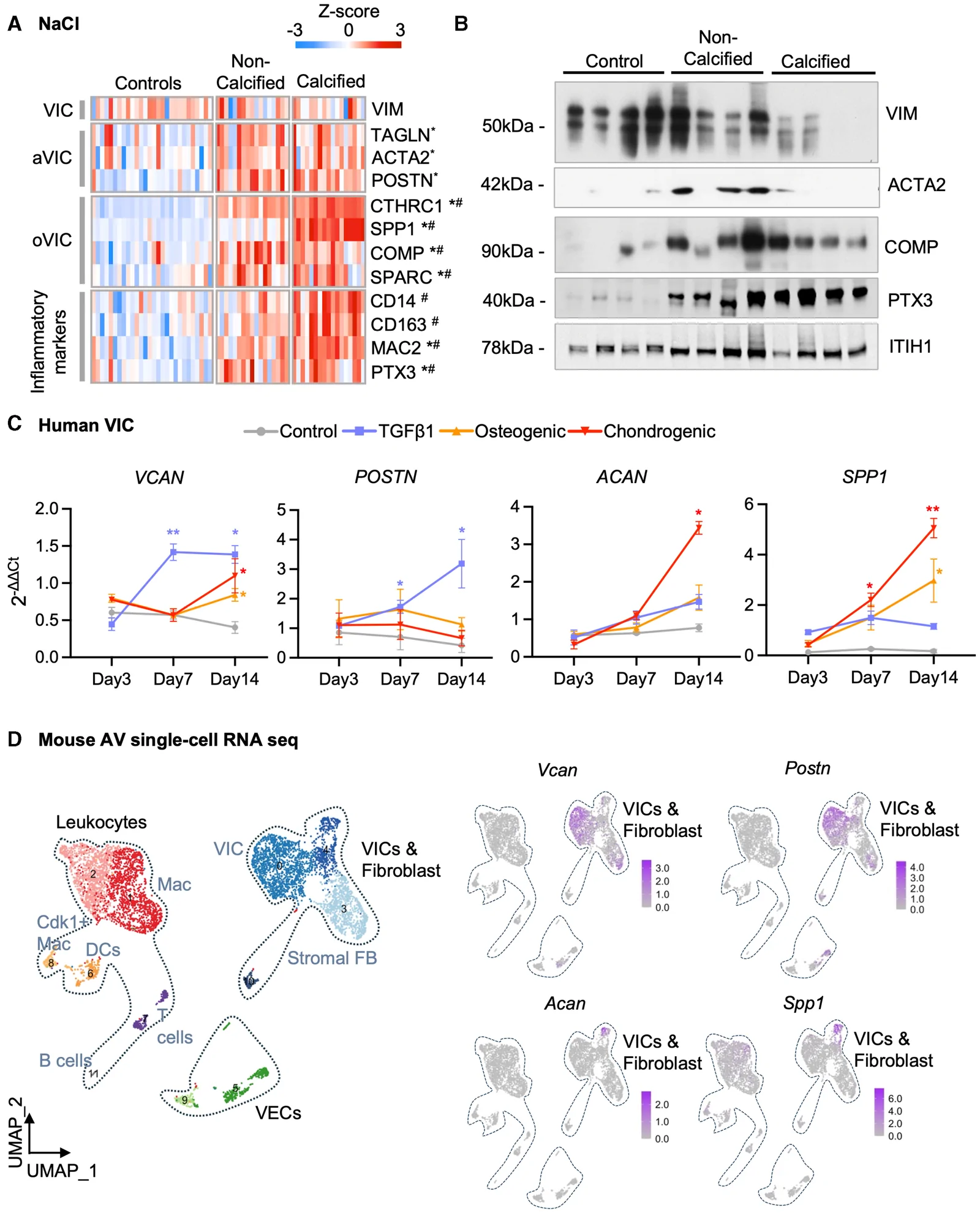
**Valve interstitial cells (VIC) subpopulation marker analysis in human and mouse single-cell RNA-seq. A**, Heatmap showing normalized protein abundance of selected cell markers. For calcific aortic valve disease (CAVD) noncalcified vs controls (*P<0.05) and CAVD calcified vs controls (^#^*P*<0.05) in the NaCl fraction, significance was calculated using the limma package for CAVD vs controls with Benjamini-Hochberg correction for *P* value. **B**, Immunoblot validation of selected proteins among control, CAVD noncalcified, and CAVD calcified groups. Corresponding densitometry quantification in the NaCl fraction can be found in Figure S4B. **C**, Gene expression analysis of human VICs cultured in control, TGFβ1, osteogenic, or chondrogenic media shows a strong increase of *VCAN*, particularly under TGFβ1 treatment with myofibroblast marker periostin (*POSTN*), and a strong induction of *ACAN*, particularly under chondrogenic conditions with chondrogenic/osteogenic biomarker *SPP1*. Statistical significance across different time points was assessed using 1-way ANOVA followed by Tukey post hoc test, *P<0.05, and **P<0.01. Data are shown as mean±SEM. **D**, **Left**: Uniform Manifold Approximation and Projection (UMAP) of scRNA-seq data from mouse AVs. **Right**, UMAP plot showing *Vcan*, *Acan,* Periostin (*Postn*), and osteopontin (*Spp1*) expression in VICs and stromal fibroblasts (FBs; **right**). DC indicates dendritic cells; Mac, macrophages; and VEC, valvular endothelial cells.

Accumulation of a hyalectan-rich matrix in AVs may promote a microenvironment conducive to VIC activation and pathological remodeling. VICs are central to the calcification process^28^ and can adopt distinct phenotypes (Figure S4A). In calcified AVs, activated VICs (aVIC) can acquire a myofibroblast phenotype, contributing to fibrosis,^29^ whereas osteogenic VICs (oVIC) are linked to calcium deposition.^29^ These phenotypic transitions were reflected in the NaCl proteomics data, with increased aVIC markers, including transgelin (*TAGLN*), alpha-smooth muscle actin (*ACTA2*), and periostin (*POSTN*), and oVIC markers, including collagen triple helix repeat-containing 1 (*CTHRC1*), osteopontin (*SPP1*), cartilage oligomeric matrix protein (*COMP*), and osteonectin (*SPARC*; Figure 4A). PTX3 (pentraxin 3) accumulation coincided with elevated inflammatory markers, including *CD14, CD163,* and galectin-3 (*MAC2*). The proteomics findings were confirmed by immunoblotting (Figure 4B; Figure S4B). AVs from patients with CAVD exhibited elevated PTX3, indicating increased inflammatory responses. Although aVIC and oVIC marker expression was upregulated, *PTX3* transcript levels were unchanged (Figure S4C).

We next examined whether TGFβ1-related signaling and chondrogenic or osteogenic differentiation pathways (Figure S4D)^30^ contribute to the observed proteoglycan patterns. *VCAN* expression increased by day 7 during TGFβ1-induced activation of human VICs (Figure 4C; Figure S4E). In contrast, *ACAN* expression was upregulated under chondrogenic conditions at day 14 (Figure 4C; Figure S4E), accompanied by marked induction of *SPP1*. Osteogenic conditions elicited a delayed *SPP1* response. These data indicate stage- and stimulus-specific regulation of hyalectans during VIC phenotypic transitions.

To assess whether similar patterns are observed in vivo, we analyzed murine AVs. Mouse AVs exhibited a hyalectan expression profile broadly similar to that observed in human AVs, with *Vcan V1* representing the predominant isoform and *Acan* expressed at lower levels (Figure S5A). Interrogation of a single-cell RNA sequencing data set from AVs of WT and hypercholesterolemic mice^21^ (Figure 4D) revealed distinct *Vcan*- and *Acan*-expressing VIC subpopulations. Consistent with our in vitro findings, activated VICs (*Postn*^+^) predominantly expressed *Vcan*, whereas *Spp1*^+^ VIC subpopulations showed enriched *Acan* expression, consistent with a shift in hyalectan expression during VIC phenotypic transition (Figure 4D).

Source analysis indicated that *Acan*-expressing cells were predominantly derived from hypercholesterolemic mice, whereas *Vcan* expression showed a broader distribution (Figure S5B). *Spp1*^*+*^ VICs were enriched for calcification-associated markers, including *Comp* (Figure S5C), and proteoglycan-related transcripts such as *Adamts5* and *Hapln1*. These patterns support an association between hyalectan remodeling, *ADAMTS5* expression, and VIC phenotypic transition. CellChat analysis further suggested that Spp1 was primarily involved in outgoing signaling from *Spp1*^+^ VICs, with predicted interactions involving the HA receptor CD44 (Figure S5D).

### Combined Effects of Hypercholesterolemia and Hyalectan Accumulation

**Figure 5.**
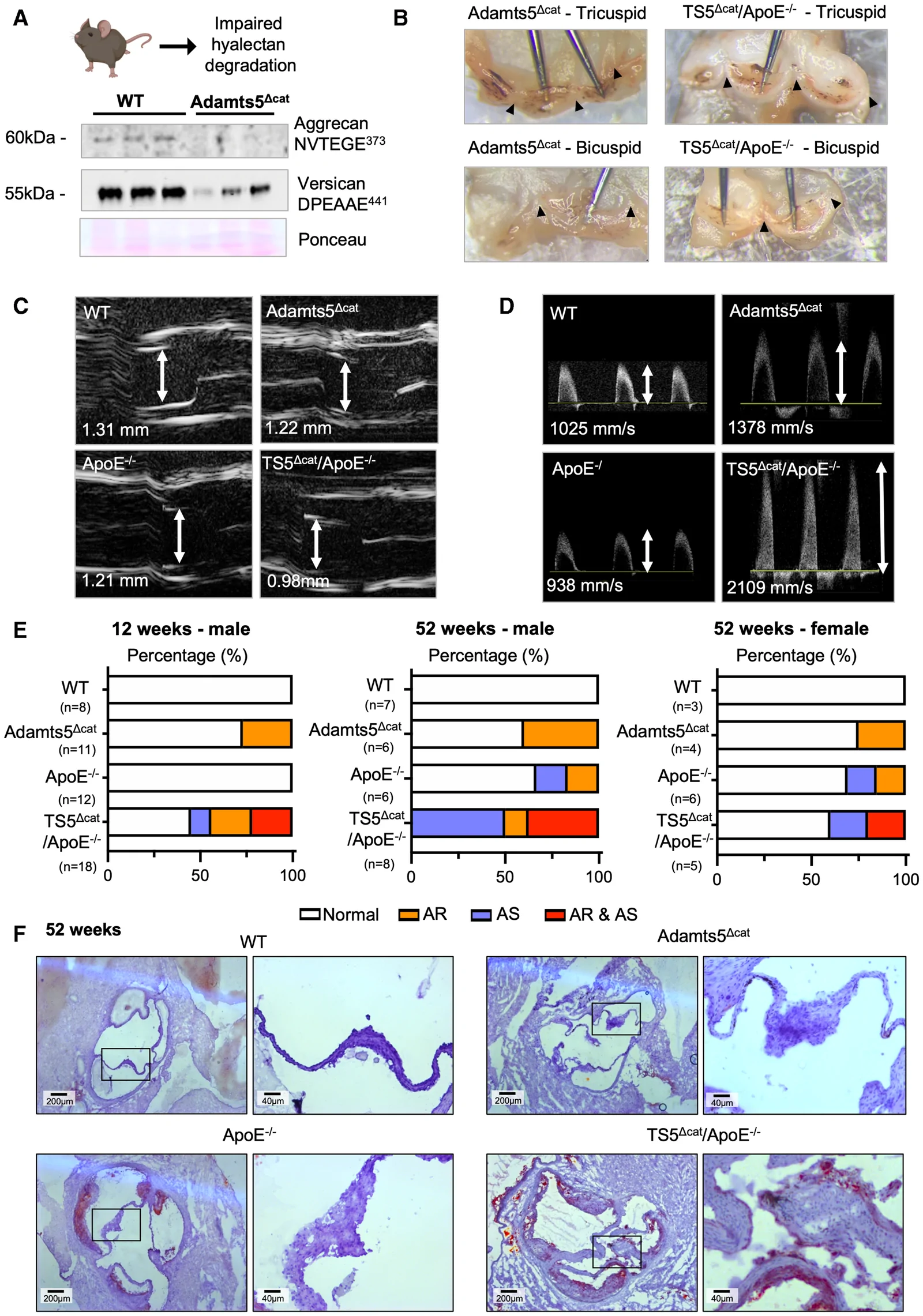
***Adamts5*^*Δcat*^*/ApoE*^−/−^ mice exhibit an aortic stenosis (AS) phenotype. A**, *Adamts5*^*Δcat*^ mice lack ADAMTS5 (a disintegrin and metalloproteinase with thrombospondin motifs) catalytic activity, hindering hyalectan cleavage. Reduced levels of ADAMTS-mediated aggrecan (NVTEGE^373^) and versican (DPEAAE^441^) fragments confirmed impaired hyalectan processing in their aortic valves (AVs). **B**, Representative images of dissected tricuspid/bicuspid AV from 12-week-old *Adamts5*^*Δcat*^ and *Adamts5*^*Δcat*^*/ApoE*^*−/−*^ mice; arrowheads indicate commissures between AV leaflets. The percentage of mice with different genotypes displaying bicuspid AVs can be found in Figure S6A. **C**, Representative aortic cusp separation distance and (**D**) mean blood velocity in 12-week-old mice and corresponding ultrasound images. Four-group data analysis can be found in Figure S6C and S6D. **E**, Percentage of 12-week-old and 52-week-old mice showing AS and aortic regurgitation (AR). **F**, Representative Oil Red O-stained images of aortic roots from 52-week-old mice. Their corresponding AV thickness and lipid deposition quantification can be found in Figure S6E. *Adamts5*^*Δcat*^*/ApoE*^*−/−*^ abbreviated as TS5^Δcat^/ApoE^*−*^^/*−*^.

Given the similarities in hyalectan expression between mice and humans, we used an existing mouse model of impaired hyalectan degradation for further mechanistic studies. In *Adamts5*^*Δcat*^ mice, other ADAMTS family members with proteoglycanase activity cannot compensate for the loss of ADAMTS5 activity (Figure 5A). Neoepitope antibodies detecting ADAMTS-specific versican (Glu441↓442Ala, DPEAAE) and aggrecan (Glu373↓Ala374, NVTEGE) fragments revealed a significant reduction in ADAMTS-mediated hyalectan cleavage. Given that *ApoE*^*−/−*^ mice model hypercholesterolemia,^31^ a key risk factor for AS in humans and mouse models,^32–34^ we crossed *Adamts5*^*Δcat*^ mice with *ApoE*^*−/−*^ mice to generate *Adamts5*^*Δcat*^*/ApoE*^*−/−*^ double-knockout mice.

Consistent with prior studies linking hyalectan remodeling to AV development,^15^ ≈10% of *Adamts5*^*Δcat*^ mice displayed a BAV phenotype, which was also observed in *Adamts5*^*Δcat*^*/ApoE*^*−/−*^ mice (Figure 5B; Figure S6A). To assess AV function, echocardiography was performed on WT, *Adamts5*^*Δcat*^, *ApoE*^*−/−*^, and *Adamts5*^*Δcat*^*/ApoE*^*−/−*^ mice at 12 weeks (Table S8) and 52 weeks (Table S9). AS and AR were evaluated using color Doppler echocardiography (Figure S6B). Transaortic blood flow was measured to assess functional impairment (Figure 5C and 5D). At 12 weeks, 30% of male *Adamts5*^*Δcat*^ mice displayed AR or AR with AS compared with 50% of *Adamts5*^*Δcat*^*/ApoE*^*−/−*^ males with AS or AR with AS (Figure 5E). By 52 weeks, disease prevalence reached 100% in males and 40% in females of the *Adamts5*^*Δcat*^*/ApoE*^*−/−*^ group (Figure 5E), suggesting the combined effects of hypercholesterolemia, hyalectan remodeling, age, and sex.

By 12 and 52 weeks, *Adamts5*^*Δcat*^*/ApoE*^*−/−*^ mice showed a greater reduction in aortic cusp separation than *ApoE*^*−/−*^ or *Adamts5*^*Δcat*^ mice (Figure S6C and S6D), with an almost 2-fold increase in mean transvalvular velocity. These changes in cusp mechanics and AV function are consistent with AS progression and may contribute to the development of AR. While ventricular weight was comparable at 12 weeks, 52-week-old *Adamts5*^*Δcat*^*/ApoE*^*−/−*^ mice exhibited significant ventricular hypertrophy (Figure S6C and S6D) compared with age-matched controls. Histological analyses^35^ revealed that *Adamts5*^*Δcat*^*/ApoE*^*−/−*^ mice had significantly thickened AV leaflets and greater lipid deposition in the AV cusp area at 52 weeks compared with *ApoE*^*−/−*^ mice (Figure 5F; Figure S6E), despite similarly elevated total cholesterol levels and no difference in body weight between the groups (Figure S6F and S6G).

To further assess the impact of hypercholesterolemia, *ApoE*^*−/−*^ and *Adamts5*^*Δcat*^*/ApoE*^*−/−*^ mice were fed a Western diet for 12 weeks (Figure S7A). With elevated cholesterol levels (Figure S7B), *Adamts5*^*Δcat*^*/ApoE*^*−/−*^ mice exhibited reduced aortic cusp separation, a change not observed in 12-week Western diet–fed *ApoE*^*−/−*^ mice or normal laboratory diet-fed controls (Figure S7C). Consistent with this finding, *Adamts5*^*Δcat*^*/ApoE*^*−/−*^ mice also displayed greater lipid accumulation in the aortic cusp area after 12 weeks of Western diet compared with controls (Figure S7D).

### Hyalectan and Pentraxin 3 Accumulation

**Figure 6.**
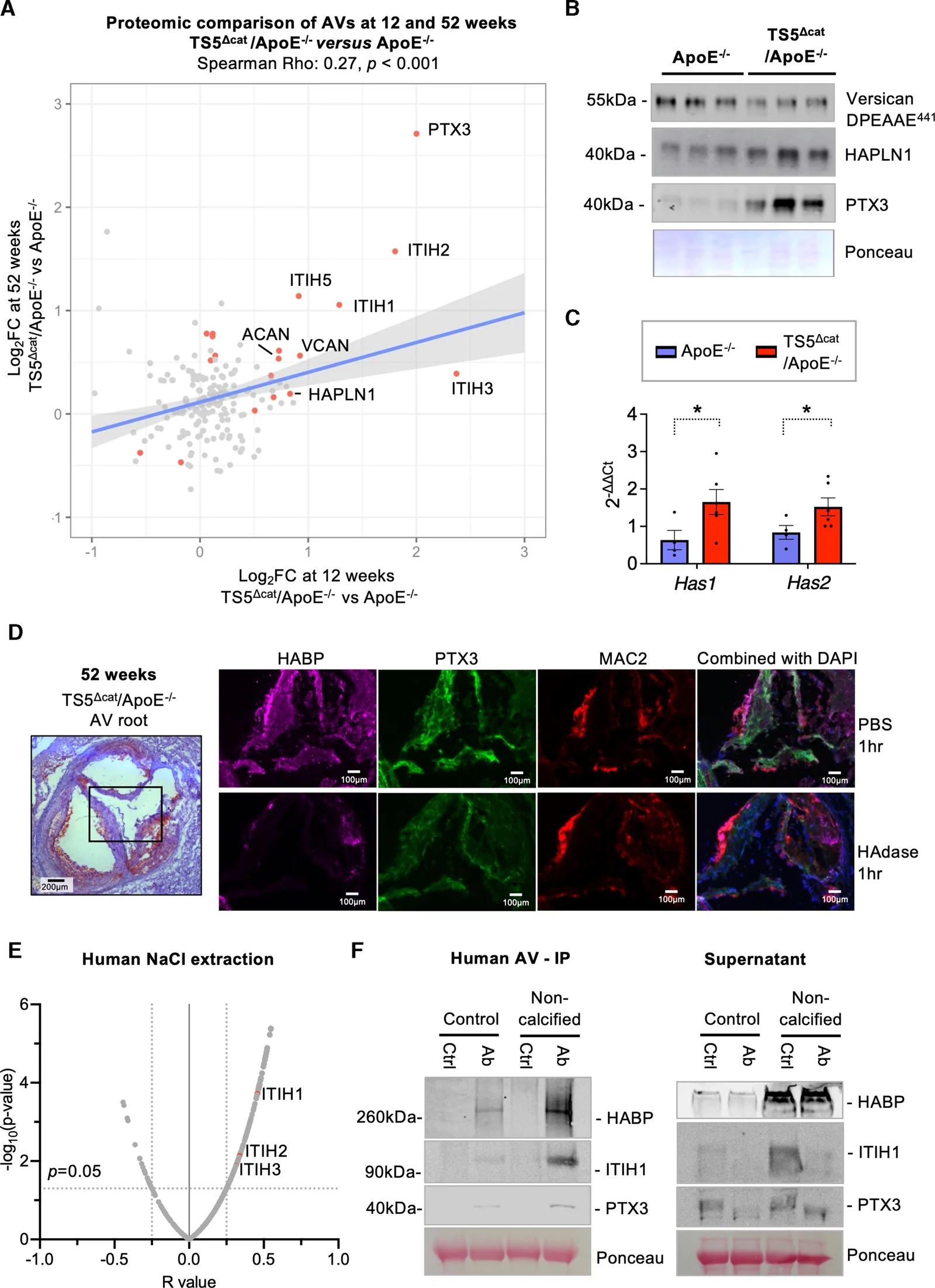
**Proteomics analysis of aortic valves (AVs) from *Adamts5*^*Δcat*^*/ApoE*^*−/−*^ mice. A**, Scatter plots highlighting common changes between 12-week male mice (*ApoE*^*−*^^*/−*^, n=8; *Adamts5*^*Δcat*^*/ApoE*^*−*^^*/−*^, n=8) and 52-week male mice (*ApoE*^*−*^^*/−*^, n=4; *Adamts5*^*Δcat*^*/ApoE*^*−*^^*/−*^, n=4). Red dots represent proteins with consistent change directions across both comparisons and significantly altered in at least one comparison. Each point represents an individual protein, and the solid blue line represents the linear regression fit. The gray shaded area indicates the 95% CI of the regression line. **B**, Immunoblots for PTX3 (pentraxin 3), HAPLN1 (hyaluronan and proteoglycan link protein 1) and the ADAMTS (a disintegrin and metalloproteinase with thrombospondin motifs)-specific cleavage product of Vcan, identified by the versican DPEAAE^441^ neoepitope. Comparisons between *Adamts5*^*Δcat*^*/ ApoE*^*−/−*^ (abbreviated as TS5^Δcat^/ApoE^*−*^^/*−*^ in figures) vs ApoE^*−*^^/*−*^ mice are shown. **C**, Gene expression levels of HA synthases (*Has1*, *Has2*) in *ApoE*^*−*^^*/−*^, and *Adamts5*^Δcat^/*ApoE*^*−*^^/*−*^ mice at 12 weeks. Statistical significance was calculated using an unpaired Student *t* test. *P<0.05. Data are presented as means±SEM. **D**, Representative immunofluorescence images showing staining for PTX3, hyaluronan (using the HABP [HA-binding protein]), and MAC2 (galectin-3) in murine AVs with 1-hour HAdase (hyaluronidase) or 1-hour PBS treatment. Nuclei are counterstained with DAPI. Corresponding staining quantification can be found in Figure S9B. *Adamts5*^*Δcat*^*/ApoE*^*−/−*^ abbreviated as TS5^Δcat^/ApoE^*−*^^/*−*^. **E**, Volcano plot of proteins extracted from human NaCl–treated samples, showing the correlation coefficient (R value) of PTX3 to other ECM-associated proteins. ITIH1 (inter-α-trypsin inhibitor heavy chain 1), ITIH2, and ITIH3 display strong positive correlations with PTX3. **F**, Immunoprecipitation (IP) from human control AVs and noncalcified AVs using an antibody against ITIH1 (labeled as Ab). The immunoblots show results for the IP pull-downs as well as the supernatants stained for hyaluronan (using the HABP), ITIH1, and PTX3.

To compare protein changes in murine AVs, we performed LC-MS/MS analysis of core matrisome extracts (GuHCl) from 12-week-old (Table S10) and 52-week-old (Table S11) *ApoE*^*-/*-^ and *Adamts5*^*Δcat*^*/ApoE*^*−/−1*^ mice. WT and *Adamts5*^*Δcat*^ mice were not included because neither develops AS-like changes under the conditions studied; therefore, this analysis focused on proteomic changes driven by ADAMTS5 deficiency in the context of hypercholesterolemia. A total of 239 ECM and ECM-associated proteins were consistently quantified. In male mice, versican, aggrecan, HAPLN1, and other HA-binding proteins, such as interalpha-trypsin ITIH1 (inhibitor heavy chain 1), ITIH2, and ITIH3, were increased in AVs of *Adamts5*^*Δcat*^*/ApoE*^*−/−*^ mice compared with *ApoE*^*−/−*^ controls. PTX3 (Figure 6A) also accumulated alongside differentially abundant proteins involved in HA binding or crosslinking. PTX3 is involved in innate immunity, inflammation, and ECM remodeling. In contrast, female *Adamts5*^*Δcat*^*/ApoE*^*−/−*^ mice exhibited a less pronounced ECM remodeling pattern. Although PTX3 remained significantly increased, hyalectan deposition was attenuated (Figure S8A).

Immunoblotting confirmed reduced ADAMTS-mediated hyalectan degradation and accumulation of PTX3 and HAPLN1 in AVs from male *Adamts5*^*Δcat*^*/ApoE*^*−/−*^ mice (Figure 6B). Expression of the HA synthase genes *Has1* and *Has2* was significantly upregulated in AVs from *Adamts5*^*Δcat*^*/ApoE*^*−/−*^ mice compared with *ApoE*^*−/−*^ controls (Figure 6C), whereas other HA-related genes, including *Hyal1*, *Hyal2*, *Hyal3*, and *Cd44,* were unchanged (Figure S8B). qPCR analysis showed increased *Adamts4* expression in AVs of *Adamts5*^*Δcat*^*/ApoE*^*−/−*^ mice (Figure S8C), suggesting a compensatory response. However, this increase did not restore ADAMTS-mediated hyalectan cleavage, as indicated by reduced ADAMTS-specific neoepitope staining. In contrast to our previous findings in aortas from Adamts5^Δcat^ mice, no compensatory increase in *Adamts1* expression was observed in AVs.^36^

Similar to findings in AVs from patients with CAVD, AVs from *Adamts5*^*Δcat*^*/ApoE*^*-/*-^ mice showed significant upregulation of the aVIC marker *Tgfb1* and oVIC markers, including *Spp1* and *Comp* (Figure S8D). *Ptx3* transcript levels did not differ despite elevated protein levels. To determine whether these changes were intrinsic to VICs lacking ADAMTS5 activity, we cultured VICs from WT and *Adamts5*^*Δcat*^ mice. VICs from *ApoE*^*−/−*^ mice were not used, allowing the cell-autonomous consequences of ADAMTS5 deficiency to be isolated from systemic effects of hypercholesterolemia. VICs lacking ADAMTS5 activity exhibited reduced versican cleavage, accumulation of intact versican in conditioned media (Figure S8E), and increased expression of *Tgfb1* and *Spp1*.

We further examined inflammatory cell involvement in lipid lesion formation by performing MAC2 staining on AV root sections from *Adamts5*^*Δcat*^*/ApoE*^*−/−*^ and *ApoE*^*−/−*^ mice. In 52-week-old ApoE^−/−^ mice, MAC2-positive cells were present in aortic root atherosclerotic lesions, as expected, but were not detected in AV cusps. In contrast, *Adamts5*^*Δcat*^*/ApoE*^*−/−*^ mice exhibited MAC2-positive cells within lipid-rich regions of AV cusps, alongside expected staining in adjacent aortic root atherosclerotic lesions (Figure S9A). Aortic root atherosclerotic lesion burden was not quantified, as the analysis was focused on AV pathology. Immunofluorescence analysis demonstrated that hyaluronidase treatment markedly reduced HA staining and substantially decreased PTX3 signal, whereas MAC2 co-staining was unaffected. Residual PTX3 staining likely reflects intrinsic expression. These findings support an HA-dependent component of PTX3 accumulation (Figure 6D; Figure S9B). To determine whether elevated tissue PTX3 levels in *Adamts5*^*Δcat*^*/ApoE*^*-/*-^ AVs were reflected systemically, we quantified plasma PTX3 by ELISA in mice aged 12 and 52 weeks (Figure S9C). At 12 weeks, no significant differences were observed among genotypes. By 52 weeks, *Adamts5*^*Δcat*^*/ApoE*^*−/−*^ mice exhibited significantly higher plasma PTX3 than WT controls but not than *ApoE*^*−/−*^ mice.

ITIH1, a well-characterized HA-binding protein, has been reported to facilitate PTX3 retention within HA-rich matrices.^37^ Correlation analysis of PTX3 with ECM-associated proteins in human AV extracts revealed significant associations with established HA-binding proteins, including ITIH1, ITIH2, and ITIH3, which are known to anchor circulating PTX3 (Figure 6E). To further examine this interaction, immunoprecipitation was performed on protein extracts from human control and noncalcified AVs using an ITIH1 antibody. Both HA and PTX3 coprecipitated with ITIH1, as demonstrated by their presence in the pull-down fraction and corresponding reduction in the supernatant (Figure 6F). Western blot analysis under nonreducing conditions confirmed that PTX3 was recovered predominantly in its multimeric form (Figure S9D), the biologically active configuration associated with high-avidity HA binding and stabilization of HA-rich matrices.^37^ Immunoperoxidase staining localized PTX3 primarily to the subendothelial region in control AVs, whereas in calcified AVs, PTX3 was enriched in areas of ECM disruption and early calcification (Figure S9E).

## Discussion

Abnormal ECM remodeling is a hallmark of CAVD.^38^ To our knowledge, this study represents the most comprehensive ECM-focused proteomic analyses on CAVD to date, based on 126 human protein extracts and integrating untargeted, targeted, and glycoproteomic approaches. Although previous proteomic analyses have provided valuable insights into CAVD,^39–43^ our study builds on these publications by employing sequential ECM protein extraction to achieve a more comprehensive understanding of ECM remodeling.

Conventional cellular lysis buffers are insufficient to extract core ECM components such as aggrecan, owing to their poor solubility and tight integration within the matrix. To enable efficient ECM recovery and comprehensive matrisome profiling, we therefore performed a 48-hour extraction using highly stringent conditions (4 mol/L GuHCl). Comparative ECM proteomic analysis of control AVs and AVs from patients with AS revealed differential regulation of proteoglycans associated with compression-resistant ECM pathways in CAVD. Specifically, versican was significantly enriched in noncalcified regions, whereas calcified regions exhibited a relative shift from versican toward aggrecan. In addition, the accumulation of HA-binding proteins such as PTX3 further highlights the value of proteomic analysis in CAVD, as several key ECM alterations are predominantly detectable at the protein rather than the transcript level.

Proteoglycans are key ECM components in AV leaflets.^7^ SLRPs primarily contribute to collagen organization, while hyalectans, such as versican, bind to HA and play essential roles in hydration and lubrication within AV leaflets.^38^ These functions are mediated by spatially and temporally regulated glycosylation.^44^ Our findings indicate that the aggrecan/versican ratio in CAVD is positively correlated with the AV pressure gradient, a critical indicator of CAVD severity. Notably, the shift from versican toward aggrecan resembles ECM remodelling during cartilage development preceding endochondral ossification, the process by which a cartilage template is progressively replaced by bone: versican-rich provisional matrices are permissive for progenitor cell migration and proliferation, whereas aggrecan accumulates during chondrocyte differentiation to establish a more mature, cartilage-specific ECM. This cartilage template subsequently undergoes calcification.^45^ A similar chondrogenic-to-osteogenic ECM process may occur during CAVD development. Our in vitro experiments and single-cell RNA sequencing analysis linked *Acan* and *Vcan* expression to distinct VIC subpopulations, with *Vcan* present in myofibroblast-like aVICs and *Acan* restricted to calcification-associated *Spp1+* oVICs.^46^ Moreover, SPP1, an osteogenic differentiation marker of VICs, was primarily involved in the outgoing communication of *Spp1+* VICs through the HA receptor CD44. Notably, loss of ADAMTS5 activity was sufficient to induce VIC transdifferentiation toward an *Spp1+* oVIC phenotype. Supporting this, COMP, an ECM glycoprotein known to interact with aggrecan, showed similar spatial enrichment in calcified regions, whereas PTX3 aligned more closely with versican -rich, activated matrices.

Together, these findings support a model of staged ECM remodeling in CAVD. Versican enrichment in aVICs may promote the formation of HA-rich matrices that support PTX3 retention during early inflammatory remodeling. As disease progresses, a shift toward aggrecan expression within *Spp1*^*+*^ VICs coincides with COMP enrichment, consistent with established COMP-aggrecan interactions in cartilage. This coordinated remodeling of HA-associated proteoglycans and other ECM-binding proteins is accompanied by increased expression of ADAMTS5 and HAPLN1. Collectively, these findings link inflammatory signaling with structural ECM maturation during CAVD progression and support the concept that CAVD involves a cartilage-like ECM stage preceding mineralization.

Due to the lack of animal models that capture hyalectan remodeling in AV disease development, we crossed *ApoE*^*−/−*^ with *Adamts5*^*Δcat*^ mice,^15,16^ allowing us to investigate whether aggrecan and versican accumulation affects AV function under hypercholesterolemic conditions. *Adamts5*^*Δcat*^*/ApoE*^*−/−*^ mice showed impaired AV function, increased AV thickness, and lipid lesion deposition, with disease severity aggravated by age and Western diet, consistent with established risk factors for AS in humans.

In AVs of *Adamts5*^*Δcat*^*/ApoE*^*−/−*^ mice, we observed an accumulation of hyalectans and HA, along with HA-binding proteins. HA comprises around 35% of the total glycosaminoglycans in normal AV leaflets and further accumulates in noncalcified regions of diseased AVs.^11,47^ As a glycosaminoglycan, HA is not detectable by proteomics; instead, proteomics identifies proteins associated with HA, such as HA-binding proteins ITIH1, ITIH2, and ITIH3 (Figure 6A). PTX3 does not directly bind HA but interacts with heavy chains of ITIH, facilitating its incorporation into HA-rich matrices.^37^ This interaction also explains the marked increase in PTX3 protein levels in both human and murine diseased AVs, despite the absence of corresponding changes in gene expression. In staining of human AVs, we observed an enhanced and disrupted pattern of spatial localization of PTX3 and HA around the calcified regions, suggesting changes in ECM organization that may influence the localization of inflammatory mediators such as PTX3 and affect cellular responses. Our findings align with previous reports identifying circulating PTX3 as an independent predictor of AV calcification,^48,49^ with strong correlations between plasma and tissue PTX3 levels.^49^ PTX3 has also been implicated in promoting osteogenic differentiation via HA/CD44-dependent signaling pathways.^50^

Changes in ECM composition between *Adamts5*^*Δcat*^*/ApoE*^*−/−*^ and *ApoE*^*−/−*^ mice were less pronounced in females, consistent with their comparatively milder disease phenotype. Although PTX3 remained significantly elevated, hyalectan deposition did not differ significantly from *ApoE*^*−/−*^ controls in female mice. In our work on human carotid plaques, we reported sex-related differences in HA-binding proteoglycans,^18^ including an inverse association between hyalectan content and circulating estradiol levels. These findings are consistent with a potential modulatory role of female hormones in regulating hyalectan deposition.

The *Adamts5*^*Δcat*^*/ApoE*^*−/−*^ mouse model was designed to investigate the combined effects of hyalectan accumulation and hypercholesterolemia in AS. However, it does not fully recapitulate the advanced calcification observed in human CAVD. Although ADAMTS5 demonstrates high specificity for hyalectans,^51^ other substrates may also be impacted by the loss of ADAMTS5 activity. To investigate broader changes in the ECM, we performed a proteomics analysis of the AV leaflets from *Adamts5*^*Δcat*^*/ApoE*^*−/−*^ mice. The analysis revealed a predominant increase in HA-binding proteins such as PTX3 alongside hyalectans. Our study specifically focused on *Adamts5*^*Δcat*^*/ApoE*^*−/−*^ mice with tricuspid valves. The low incidence of BAVs in this mouse model (≈10%) limits the feasibility of generating statistically meaningful cohorts. Although 2 of the 17 human CAVD samples exhibited a BAV phenotype, exclusion of these cases did not materially affect the key findings (Figure S10). A substantially larger and more balanced study population will be required to assess sex-related effects on ECM remodeling in human CAVD.

In summary, this study highlights hyalectan remodeling as a prominent feature of CAVD pathogenesis. Through comprehensive proteomic and glycoproteomic analyses of the ECM matrisome in human CAVD, we demonstrated that hyalectan remodeling correlates with CAVD severity. In *Adamts5*^Δcat^/*ApoE*^*−/−*^ mice, loss of ADAMTS5 activity resulted in hyalectan accumulation, AV leaflet thickening, and structural abnormalities, recapitulating key ECM features observed in patients with CAVD. Notably, the lack of ADAMTS5 activity was sufficient to promote VIC activation, as indicated by increased *SPP1* expression and hyalectan accumulation linked to PTX3 deposition. Collectively, these findings support hyalectan remodeling as a disease-relevant ECM pathway and potential therapeutic target in CAVD. Future studies should explore therapeutic approaches targeting hyalectan- and HA-related mechanisms to mitigate the consequences of progressive proteoglycan accumulation. A chimeric mouse/human monoclonal antibody has been developed^52^ to prevent glycosaminoglycan chain binding to LDL, reducing atherosclerotic lesion development. Moreover, a pegylated recombinant human hyaluronidase has already undergone clinical testing as an anticancer agent.^53^ In parallel, circulating ADAMTS-derived proteoglycan fragments could provide clincially accessible biomarkers of tissue-level ECM remodeling in CAVD.

## ARTICLE INFORMATION

### Acknowledgments

All authors have read and approved the final article. The authors thank Dr Ella Reed (King’s College London, UK) for her contribution to mouse sample collection and preliminary processing. The authors gratefully acknowledge Jenny Ho of Thermo Fisher Scientific (Hemel Hempstead, UK) for her valuable contribution to the glycoproteomic sample running.

### Disclosures

None.

### Supplemental Material

Expanded Materials & Methods

Tables S1–S11

Figures S1–S10

Major Resources Table S12

## Supplementary Material
