# Supplementary material for: Aortic Stenosis Hyalectan Remodeling Revealed by Proteomics and Glycoproteomics
Source: Arterioscler Thromb Vasc Biol. 2026 Jul 16;46(9):e323867. doi: 10.1161/ATVBAHA.126.323867 (PMC13506200; doi:10.1161/ATVBAHA.126.323867)
Supplement: Supplementary file 1 [file atv-46-e323867-s001.pdf]

SUPPLEMENTAL MATERIAL

Aortic Stenosis Hyalectan Remodeling Revealed by Proteomics and Glycoproteomics

Siqi Yin[1], Ursula Mayr[1], Javier Barallobre-Barreiro[1], Elisa Duregotti[1], Anna K. Barton[3], Rong Bing[3], Dyana Markose[4], Xiaoke Yin[1], Padmini Sarathchandra[1], Bhawana Singh[1], Wen-Yu Lin[5], Marika Fava[2], Lukas E. Schmidt[6], Ferheen Baig[2], Ajay M. Shah[2], Konstantinos Theofilatos[2], Najma Latif[1], Christian Hengstenberg[6], Tamás Radovits[7], Béla Merkely[7], Neil C. Henderson[4], Marc R. Dweck[3], Manuel Mayr[1]

[1] National Heart and Lung Institute, Imperial College London, London, UK

[2] School of Cardiovascular and Metabolic Medicine & Sciences, King's College London British Heart Foundation Centre, London, UK

[3] British Heart Foundation Centre for Cardiovascular Science, University of Edinburgh, Edinburgh, UK

[4] Centre for Inflammation Research, Institute for Regeneration and Repair, University of Edinburgh, Edinburgh, UK

[5] Division of Cardiology, Department of Internal Medicine, Tri-Service General Hospital, National Defense Medical Center, Taipei, Taiwan

[6] Department of Internal Medicine II, Division of Cardiology, Medical University of Vienna, Vienna, Austria

[7] Heart and Vascular Center, Department of Cardiology, Semmelweis University, Budapest, Hungary

SUPPLEMENTAL METHODS

A Materials and Reagents Table is provided in **Supplemental Table S12**.

Human Sample Collection

We obtained AV leaflets through a collaboration with Semmelweis University (Budapest, Hungary, approved by Internal Research Ethics Committee of King's College London, REC LRS-17/18–5080) and the University of Edinburgh (Edinburgh, UK, approved by Academic and Clinical Central Office for Research and Development, REC 16/SS/0027). Written informed consent was obtained from all patients. In total, AV leaflets from 46 patients were used in this study. AV leaflets obtained from Budapest were derived from patients with heart failure undergoing cardiac transplantation. AV leaflets from Budapest (n=29) were free from calcification and patients showed no signs of stenosis or regurgitation. AV leaflets obtained in Edinburgh were derived from patients undergoing AV replacement surgeries for severe AS (n=17). Sample collection and processing were standardized across both contributing centers to mitigate batch effects and reduce technical variability. Importantly, the AV leaflets from patients with CAVD were dissected into two distinctly different areas: non-calcified and calcified. A total of 63 samples were analyzed for all experiments. To minimize anatomical bias, sampling was distributed across comparable locations depending on tissue availability. The use of paired calcified and non-calcified regions from the same AVs helped control for inter-individual variability and enabled internal comparisons of ECM composition. The STROBE checklist was used to demonstrate adherence to the data-reporting guidelines.

Animal Experiments

All animal procedures were performed by authorized researchers in the School of Cardiovascular and Metabolic Medicine & Sciences, King's College London. Housing and animal care was in accordance with the UK Animals (Scientific Procedures) Act 1986. *Adamts5*$^{\Delta cat}$ (ie, lacking the catalytic domain of ADAMTS5; JAX stock 005771, B6.129P2-Adamts5$^{tm1Dgen/J}$) and WT littermates on a C57Bl/6J background were used. The generation of ADAMTS5 and ApoE double deficient mice (*Adamts5*$^{\Delta cat}$*/ApoE*$^{-/-}$) was achieved by crossing

ApoE$^{-/-}$ mice (JAX stock 002052, B6.129P2-Apoe$^{tm1Unc/J}$) with *Adamts5*$^{\Delta cat}$ heterozygous mice. All genetically modified mouse strains underwent at least one additional backcross to C57BL/6J mice, with the backcrossing process extending beyond five generations. Both female and male mice aged 12 and 52 weeks were used for echocardiography analysis. In general, *Adamts5*$^{\Delta cat}$ mice were compared with their *Adamts5*$^{+/+}$ littermates (WT), whereas *Adamts5*$^{\Delta cat}$/*ApoE*$^{-/-}$ mice were compared with *ApoE*$^{-/-}$ mice to assess the effect of ADAMTS5 deficiency, or to WT mice to evaluate the combined effect of ADAMTS5 and APOE deficiency. A single animal was considered the experimental unit. Due to the low sample size and the unique phenotype of mice with BAVs, data from these mice were excluded from subsequent analyses to ensure robustness and consistency in the results.

Echocardiography

All imaging studies were performed using the VisualSonics Vevo®2100 imaging system with an MS400 (18–38 MHz) or MS550D (55 MHz) linear array transducer depending on the cardiac hypertrophy status of the mouse heart. Anesthesia was induced using 5% isoflurane mixed with 1L/min of 100% oxygen. Mice were placed on a heating pad with embedded electrocardiogram electrodes in a supine position, anesthesia was maintained by inhalation of 1.5% isoflurane in 98.5-99.0% O_2 at a flow rate of 1 L/min. Body temperature and heart rate were monitored throughout the whole procedure, maintained at 37.0 °C ± 1.5 °C and below 500bpm, respectively. Two-dimensional (B-Mode) images were recorded in left parasternal long- and short-axis projections to assess cardiac function, with guided one-dimensional (M-mode) recordings at the mid-ventricular level (for left ventricle wall thickness measurement), at the apical region of the papillary muscle and at the aortic root level (for cusp separation distance measurement). Cine loops of ≥3 consecutive cardiac cycles were recorded for offline analysis. Pulsed-wave Doppler was used to measure the aortic velocity time integral (VTI), from which the mean peak velocity and the gradient were calculated. The severity of AS/AR was assessed and documented using a combination of aortic cusp separation, transvalvular blood flow velocity and Color Doppler. Maximal aortic diameter and cusp separation measurements were obtained at mid-systole, defined as the frame

corresponding to maximal valve opening and peak anterior motion of the aortic cusps. An initial pilot study (n=3) was conducted to estimate the optimal sample size per group. Due to the incomplete penetrance of the phenotype in *Adamts5*$^{\Delta cat}$*/ApoE*$^{-/-}$ mice at 12 weeks of age, additional mice were included to account for the observed variability. The final sample size was also influenced by the availability of mice during the experimental period. Echocardiography data were obtained independently by two observers and analyzed in a blinded manner. After post-mortem analysis, animals displaying a bicuspid AV phenotype were excluded from further analysis due to their low number and heterogeneous echocardiographic presentation, which precluded meaningful statistical comparison.

Tissue Harvesting and Serum Collection in Mice

Mice were euthanized by administration of a sodium pentobarbital overdose. Hearts were washed and excised in cold phosphate-buffered saline (PBS). For proteomics and RNA isolation, AV cusps were snap-frozen at -80°C. For histology, mouse hearts were snap-frozen in OCT in -80°C. The AV status was examined during tissue harvesting, mice with bicuspid AV were excluded from the subsequent analysis.

Whole blood was obtained from mice via cardiac puncture after overdosing with intraperitoneally administered pentobarbital. Blood was gently aspirated into a 1ml syringe with 25G needle then transferred to a sterile Eppendorf tube. Blood samples were then left to clot at room temperature for 30-45 min and centrifuged thereafter for 15 min at 2000 x g (with no brake). After centrifugation, the upper phase (serum) was transferred to a clean tube and stored at -80°C. Serum cholesterol levels were assessed using the Cholesterol Assay Kit in accordance with the manufacturer's instructions.

PTX3 ELISA measurement

Pentraxin 3 levels in mouse plasma were measured using the Mouse Pentraxin 3/TSG-14 ELISA Kit (Bio-Techne) according to the manufacturer’s instructions. Briefly, sample and standard solutions were added to the pre-coated wells and incubated, followed by washing and incubation with the detection antibody. After the addition of the substrate solution,

absorbance was measured at 450 nm using a microplate reader. PTX3 concentrations in the samples were calculated from the standard curve generated within each assay.

Western Diet (WD) Feeding

Male and female $ApoE^{-/-}$ and $TS5^{\Delta cat}/ApoE^{-/-}$ were fed either a normal laboratory diet (ND; 13.1%kcal fat, 24.5%kcal protein, 62.3%kcal carbohydrates) or a Western diet (WD; SDS Diets; http://sdsdiets.com; 34.2%kcal fat, 16.1%kcal protein, 49.7%kcal carbohydrates, 0.25% cholesterol) from 6-8 weeks of age for 12 weeks. At this point, tissues were collected under terminal anesthesia when the animals were 18 weeks old. Echocardiography was conducted before the diet introduction (baseline) and before harvesting.

Proteomics Analysis of Human and Mouse AV samples

ECM Extraction

Diced human AV leaflets were weighed and washed with PBS to remove the plasma protein contamination. 1:100 (v:v) of a broad-spectrum protease inhibitor cocktail and 25mM EDTA were added to the PBS to ensure inhibition of proteases. Extracellular matrix (ECM) protein enrichment of AV samples was performed using an adaptation of a previously published 3-step extraction method from our lab.[17,18] For human AV samples, 40mg diced leaflets were placed on a shaker and incubated with a 0.5M sodium chloride (NaCl) solution (0.5M NaCl, 10mM Tris, pH 7.5, 25mM EDTA, supplemented with 1:100 (v:v) of proteinase inhibitor cocktail, in a 10:1 volume:sample weight ratio) for 1 hour, followed by incubation with 4M guanidine hydrochloride (GuHCl) solution (4M GuHCl, 50mM sodium acetate, pH 5.8, 25mM EDTA supplemented with 1:100 (v:v) of proteinase inhibitor cocktail, in a 5:1 volume:sample weight ratio) on a shaker for 48 hours. Protein concentration was measured using the Pierce Bradford Protein Assay Kit. The protein extracts were subjected to proteomics analyses and validation by immunoblotting.

For mouse AV samples, the protein extraction steps were similar to the human samples. Briefly, the diced tissues were placed on a shaker and incubated with 4M GuHCl for 48 hours with three 1.4mm ceramic beads (zirconia). Protein extracts pooled from 2 murine

AV leaflets were used for Western blot validation.

Deglycosylation of ECM Extracts

For proteomics of human samples, 40µg of protein from NaCl and GuHCl extracts were incubated with 100% acetone and 100% ethanol (1:10 volume ratio), respectively at -20°C overnight. For mouse samples, 7µg of protein from GuHCl extracts were incubated with acetone (1:10 volume ratio) at -20°C overnight. After incubation, proteins were precipitated by centrifugation at 16,000 g for 1h at 4°C. Supernatants were removed and protein pellets were dried using a speed vacuum (Thermo Fisher Savant SpeedVac SPD131DDA). For protein deglycosylation (i.e. removal of glycans attached to proteins), a two-step protocol was pursued. In the first step, samples were resuspended in deglycosylation buffer (50mM Tris, 50mM sodium acetate, 25mM EDTA, 12.5mM sodium phosphate, pH 6.8) containing Chondroitinase ABC (1:100), Heparinase II (1:500) and Keratanase (1:500) for glycosaminoglycan (GAG)-removal and three additional enzymes that ensure debranching of sugar residues: α2-3,6,8,9-Neuraminidase, β1,4-Galactosidase and β-N-Acetylglucosaminidase (1:200). Endo-α-N-acetylgalactosaminidase was added to ensure removal of O-glycosylation. The samples were incubated for 1 hour at 25°C and 24 hours at 37°C, with shaking at 200rpm. Afterwards, samples were dried in a speed vacuum and resuspended in same volume of $H_2{}^{18}O$ containing 1:100 PNGase F (N-glycosidase F). Subsequently, samples were incubated under constant agitation, at 37°C for 48 hours. Deglycosylation in the presence of $H_2{}^{18}O$ ensures the incorporation of ^{18}O into asparagines at deglycosylation sites, with the resulting mass shift enabling the identification, characterization and quantitation of glycopeptides.

In-solution Digestion

The peptide yield obtained from prior tryptic digestion of AV extracts was determined using a Quantitative Colourimetric Peptide Assay. Based on this yield, the protein extract volumes required to achieve a peptide yield of 7 µg after digestion were calculated.

Deglycosylated NaCl and GuHCl extracts were denatured by the addition of 9M urea, 3M thiourea (final conc. 6M urea, 2M thiourea), and reduced by the addition of 100mM dithiothreitol (final conc. 10mM) followed by incubation at 37°C for 1 hour, 180rpm. The

samples were cooled to RT before being alkylated by adding 500mM iodoacetamide (final conc. 50mM), followed by incubation in the dark for 1hour. To precipitate protein, pre-chilled (-20°C) acetone (8x sample volume) was added, and the samples were incubated overnight at -20°C. Samples were centrifuged at 14,000 g for 25 minutes at 4°C and the supernatant subsequently discarded.

Protein pellets were dried using a speed vacuum (Thermo Scientific, Savant SPD131DDA), re-suspended in 0.1M triethylammonium bicarbonate (TEAB) buffer, pH 8.2, containing trypsin (1:50, trypsin:protein) and digested overnight at 37°C, 180rpm. The digestion was stopped by acidification with 10% Trifluoroacetic acid (TFA) (final concentration 1% TFA).

C18 Clean-up of ECM Extracts

Peptide samples were purified using a 96-well C18 spin plate (Harvard Apparatus). The resin was activated using 200µl methanol and centrifuged at 1,000 g for 1 minute. Wash steps included 200µl of 80% acetonitrile (ACN), 0.1% TFA in H_2O, and three equilibration steps using 200µl of 1% ACN, 0.1% TFA in H_2O with centrifugation (1,000 g for 1 minute) after each step. Samples were loaded onto the resin and centrifuged at 2,000 g for 1 minute; the flow-through was reloaded onto the resin a second time and centrifugation repeated. The resin was then washed three times with 200 µl 1% ACN, 0.1% TFA in H_2O (centrifugation at 1,500 g for 1 minute). Finally, the samples were eluted with 170µl of 50% acetonitrile, 0.1% TFA in H_2O (centrifugation at 1,500 g for 1 minute); this step was repeated, combining the collected eluate. The eluates were dried using a speed vacuum and re-suspended in 0.05% TFA in 2% ACN for analysis by liquid chromatography–tandem mass spectrometry (LC-MS/MS).

Untargeted Proteomics

The purified peptide samples were separated on a nanoflow LC system (Thermo Scientific, Dionex UltiMate 3000 RSLCnano). Samples were injected onto a trap column (Thermo Scientific, catalogue number 160454) at a flow rate of 25µl/min for 3min, using 0.1% formic acid (FA) in water. Two mobile phases were used: A = 0.1% FA in water; B = 80% ACN, 0.1% FA in water. The following nano LC gradient was then used to separate the peptides at

0.25µl/min by gradually increasing the percentage of mobile phase B: 0–5min, 4%–10% B; 5–75min, 10%–30% B; 75−80min, 30%−40% B; 80–85min, 40%–99% B; 85–89.8min, 99% B; with a final column re-equilibration stage by dropping the percentage B to the initial stage, 89.8–90min,99%–4% B; 90–120min, 4% B. The nano column (Thermo Scientific, ES803A) was kept at 45°C and coupled to an EASY-Spray Source (Thermo Scientific).

The eluate was sprayed into a Q Exactive HF mass spectrometer (Thermo Scientific) configured for data-dependent acquisition using a full MS/data-dependent MS2 setup. Survey full scan spectra were acquired in Orbitrap over the mass-to-charge (m/z) range 350–1,600 and a resolution of 60,000 at 200m/z. Dynamic exclusion duration was 40s. Data-dependent MS^2 scans were performed on the top 15 ions in each full scan (resolution of 15,000 at 200m/z).

Database Search and Data Filtering

LC-MS data were processed using Proteome Discoverer (Thermo Scientific, version 2.3.0.523) and Mascot (version 2.6.0, Matrix Science). Raw files were searched against a human UniProtKB/Swiss-Prot protein database (version 2020_01, 20365 protein entries) or a mouse UniProtKB/Swiss-Prot protein database (version from January 2021, 17,063 entries). The mass tolerance was set at 10 ppm for precursor ions and 20 mmu for fragment ions. Trypsin was set as the protein-digesting enzyme with up to two missed cleavages being allowed. Carbamidomethylation of cysteine was set as a static modification, and oxidation of lysine, methionine, proline, and deglycosylation of asparagine with the presence of $H_2{}^{18}O$ were set as dynamic modifications. Before further analysis in Proteome Discoverer, the data were filtered to include only proteins with a minimum of two unique peptides per protein and a high confidence. The confidence level was determined using Proteome Discoverer's Protein False Discovery Rate (FDR) Validator node, with a threshold of 0.01.

Targeted Proteomics

A parallel reaction monitoring (PRM) method was developed with the help of Skyline software (version 4.1, MacCoss Lab Software). Data from the untargeted analysis were used to select proteotypic peptides of ECM proteins. Precursor ions for ECM proteins of interest not detected in the untargeted analysis were predicted *in silico* using SRMAtlas. Skyline was also

used to optimize retention times. Proteotypic peptides were scheduled using the retention time obtained from test experiments with the same LC configuration and eluting gradient; retention time windows were set at ± 4 min. After initial testing, samples were quantified for a final total of 210 peptides from 118 ECM proteins (see **Supplemental Table 4** for entire list).

Samples were injected onto a nano-trap column (Acclaim PepMap100 C18 Trap, 5 mm x 300 µm), at a flow rate of 25 µL/min for 3 minutes, using 0.1% FA in H_2O. The following LC gradient was then used to separate the peptides at 0.25 µL/min: 0–20 min, 4–8% B; 20–190 min, 8–25% B; 190–210 min, 25–40% B; 210–215 min, 40–99% B; 215–219.8 min, 99%B, 220–250 min 4%B.

Spectra were collected from an Orbitrap mass analyzer (Q Exactive HF, Thermo Scientific) using PRM mode (MS2 resolution of 30,000 at 200 m/z). An isolation window of 2.0 m/z, automatic gain control values of 2E5 and a maximum injection time of 60ms was used. Fragmentation was performed with a normalized collision energy of 28, and MS/MS scans were acquired with a fixed first mass of 110.0 m/z. The identity of a specific peptide was confirmed by the presence of multiple transitions at the same retention time. All peaks were reviewed and integrated manually. Quantification of each peptide was performed by summing the peak areas of all fragment ions. A minimum of three fragment ions and a signal-to-noise ratio of 3 to 1 were required for a peptide to be considered quantifiable. Total ion intensity values from full scan data were used for normalization. For proteins with more than one peptide, peptide correlation was checked. To be included in the analysis, peptides belonging to the same protein were required to exhibit a strong correlation ($R^2 \geq 0.65$), while low abundant peptides and peptides with poor correlation were excluded. KNN-Impute algorithm was used to impute the values of missing data.

Glycopeptide Enrichment

For glycoproteomics analysis, glycopeptide enrichment was performed using an adaptation of our previously published protocol.[19]

35µg of protein from GuHCl extracts were incubated with 100% ethanol (1:10 volume ratio) at -20°C overnight. After incubation, proteins were precipitated by centrifugation at

16,000 g for 1h at 4°C. Supernatants were removed and protein pellets were dried using a speed vacuum and resuspended in 6M urea, 2M thiourea, 0.1M TEAB (pH=8.5). After reduction with 10 mM dithiothreitol at 37°C for 1 hour, 240 rpm, the samples were cooled down to room temperature before being alkylated by 50 mM iodoacetamide in the dark for 1 hour. Prechilled (−20°C) acetone (8x volume) was used to precipitate the samples overnight at −20°C. Samples were precipitated by centrifugation at 16,000 g for 1h at 4°C and the supernatant subsequently discarded. Protein pellets were dried using SpeedVac, resuspended in 170 µL of 0.1 M TEAB (pH=8.2), and digested with 1.4 µg of trypsin/LysC at 37°C overnight. The digestion was stopped with 1% TFA.

The peptides were purified using a C18 cartridge on a Bravo AssayMAP robot (Agilent) to ensure high-quality samples for subsequent LC-MS/MS analysis. The eluted peptides were dried using SpeedVac and resuspended in 35µL of 0.1M TEAB. Peptides were labelled using the TMTpro 16plex Isobaric Tandem Mass Tag Labeling Reagent Set (TMTpro), according to the manufacturer's instructions (Thermo Fisher Scientific). A pooled sample consisting of aliquots from all individual samples was used as a control and labelled with TMTpro-126.

TMTpro-labelled peptides were further enriched for glycopeptides using zwitterionic hydrophilic interaction liquid chromatography (Agilent Poroshell 120 HILIC-Z 2.7um, 2.1mm x 100mm, PN 685775-924) and separated into 16 fractions.

Glycoproteomic Analysis

The glycopeptide enriched fraction was separated using the Vanquish Neo nanoflow liquid chromatography (LC) system (Thermo Fisher Scientific) with a C18 column (EASY-Spray, 50 cm×75 µm, Thermo Fisher Scientific). Peptides were eluted using 5% to 35% B (80% acetonitrile, 0.1% formic acid in H_2O) within 70min at a flow rate of 300 nL/min and directly analyzed with an Orbitrap Ascend MS (Thermo Fisher Scientific) with electron transfer dissociation (ETD). The Orbitrap Ascend was operated in DDA mode (data dependent acquisition) where high resolution FT-MS scans (m/z 380-1500, resolution 120,000 (at m/z 200) and alternating ETD and HCD FT-MS2 scans (m/z 110-2000, resolution 45,000 (at m/z 200) were acquired. Precursor selection for MS2 included charge states 2-6, 40s dynamic

exclusion window, intensity threshold 5e4 and quadrupole isolation width of 0.7m/z. Precursors meeting the criteria were prioritized based on highest charge state and most intense. The same precursor was fragmented by ETD and HCD within a 3s cycle time. ETD parameters included calibrated ETD reaction times and precursor AGC of 2e5 and 91ms. HCD parameters include 34 normalized collision energy and precursor AGC 2e4 and 91ms. N-glycopeptide and O-glycopeptide were identified using Byonic (version 2.9.30, Proteome Metrics) with built-in N-glycan and O-glycan databases and a custom-made protein database (1526 protein entries) from the previous LC-MS/MS experiment using deglycosylated samples. To detect TMTpro-labelled peptides, the modifications specified in the quantification method (lysine and N-terminal residue modification + 304.2071 Da) were included in the search. The signal intensities of TMTpro reporter ions generated in HCD spectra were used as quantification value of the peptides. Correction for the reagent lot-specific isotopic impurities of TMTpro tags was applied. To account for variation in abundance between samples, data was normalized to the total peptide amount. After normalization, protein abundance was scaled to the average abundance of the pooled sample.

Bioinformatics Analysis of Glycoproteomics Data

The quantities of proteins and peptides were normalized using log2 transformation. The data sets were then filtered to retain only measurements with less than 35% missing values due to TMT proteomics batch effects (3 batches were performed in total), or those where one of the examined conditions had more than 90% missing values and the remaining conditions had fewer than 10%. In the latter case, missing values in the condition with over 90% missing data were imputed as zeros. All other missing values were imputed using the KNN-Impute method, with k set to the minimum of 3 or the sample size of the phenotype with the fewest samples.

Peptide-spectrum matches (PSMs) for intact glycopeptides were further validated using a score filter. A4 refers to search results from HCD spectra, providing more peptide information but less glycan information, and was used to generate TMT reporter ions. A8

represents search results from EThcD spectra, which used ETD and supplementary activation from HCD, yielding more comprehensive post-translational modification (PTM) information. In this study, we used glycan peptides with an A8 PMI-Byonic score greater than 100, or those with an A8 PMI-Byonic score below 100 but no missing values, alongside an A4 PMI-Byonic score greater than zero.

Bioinformatics Analysis of Proteomics Data

Data from NaCl or GuHCl extracts were filtered for ECM-related proteins using Matrisome annotations[22] and manually added known ECM proteins (**Supplemental Table S1**). The dataset was further filtered to keep only proteins with less than 30% missing or with more than 90% missing values in one of the examined phenotypes and less than 10% for the rest of the phenotype. In the latter case missing values of the phenotype which presented more than 90% of missing values were imputed with zeros. All remaining missing values were imputed with KNN-Impute method with k equal to 3. The relative quantities of the proteins were scaled using $\log_2$ transformation.

For proteomics data obtained from human AV samples, the limma package[23] was used to compare between CAVD non-calcified samples and control AV leaflets using the eBayes algorithm and correcting for age and sex; Student's paired t-test was used to compare between CAVD non-calcified vs CAVD calcified groups; a threshold of 0.05 was used for the obtained *p*-values after Benjamini-Hochberg correction to infer statistically significant changes. Circular heatmap, heatmap and correlation plots showing significant proteomic data changes were constructed using R packages such as ggplot2 and ComplexHeatmap. PCA analysis was conducted using sklearn package. Enrichment analysis has been conducted using DAVID Bioinformatics Resource[23] and results are presented as dot plots using R package ggplot2. For proteomics data obtained from mouse samples, Student's unpaired t-tests were used to compare between two groups. The data were tested for normality with Shapiro-Wilk test prior to Student unpaired t-test.

Standard Laboratory Techniques

Western Blot

After protein concentrations were determined, aliquots from NaCl and GuHCl extracts were enzymatically deglycosylated and denatured. Protein samples were separated on Bis-Tris gradient gels in 1x NuPage MOPs SDS Running Buffer. Subsequently, the samples were transferred to nitrocellulose membranes in ice-cold transfer buffer (25 mM Tris-Base and 200mM Glycine in 20% methanol). Ponceau S red staining was used to ensure efficient transfer and equal loading. Membranes were blocked in 5% milk in PBS with 0.1% Tween-20 (PBST) for 1 hour and incubated overnight with primary antibody solution at 4°C. The following day, the membranes were incubated for 1 hour at RT with the appropriate HRP-conjugated secondary antibody diluted in 5% milk in PBST. For chemiluminescent detection, membranes were incubated with ECL Western blotting detection reagent and exposed to X-ray film (FUJIFILM). For fluorescence detection, membranes were incubated for 1 hour at RT with appropriate fluorescently conjugated secondary antibody diluted in 0.5% milk in PBST in the dark. The membranes were then scanned using an Odyssey CLx Imager. When necessary, after ECL Western detection, the post-ECL membrane was incubated in 0.1% sodium azide for 15 to 30 minutes to permanently inactivate the previous HRP and thoroughly washed before incubating with a new primary antibody from a different host species. Western blots were quantified by densitometry using ImageJ software. Relative abundances were normalized to the corresponding Ponceau staining (all the corresponding Ponceau staining are attached with original Western Blot image), wherein the total staining intensity of the entire lane was utilized for normalization purposes.

Histology Sections and Staining of Murine Aortic Roots

Frozen mouse aortic roots were sectioned with 8μm thickness. The sections were fixed with 4% paraformaldehyde for 10 min. Oil red O staining was performed to examine the morphology and lesion depositions of AV leaflets. Histological analysis of the AV leaflet thickness was performed following previously developed methods.[35] Due to the technical difficulty of preserving all three aortic valve leaflets within a single histological section in mouse

samples, the section demonstrating the most representative visualization of all three leaflets was used for aortic valve (AV) cusp thickness quantification. Maximal leaflet thickness was measured at the midpoint of each leaflet using ImageJ. For each mouse, AV leaflet thickness was defined as the average of the three largest measurements obtained across the sections.

For immunofluorescence, detection of pentraxin3 (PTX3), versican (VCAN) neoepitope and hyaluronan (HA) was performed in AVs. After fixation with PFA, tissues were permeabilized with 0.1% Triton for 10 min at RT and blocked with 0.5% FBS PBST buffer for 1 hour at room temperature. An indirect immunofluorescence technique was used to detect the protein or GAG of interest, where slides were treated with primary antibody overnight at 4°C in the dark then incubated with secondary antibody for 1hr at RT in the dark. Sections were visualized with an inverted Nikon NI-E microscope equipped with a spinning disk confocal unit and a Leica DM2000 LED camera. For negative controls of PTX3 and VCAN neo, sections were treated with Rb IgG instead of primary antibody. For negative controls in HA staining, sections were treated with 40U/ml hyaluronidase for 1 hour at 37°C, followed by three washes for 5 minutes with PBS-T. The endogenous avidin and biotin were blocked in the experimental sections using Avidin-Biotin Blocking Kit before primary and secondary antibody treatment.

For immunoperoxide staining, 5µm thick paraffin wax sections of control and calcified leaflets were dewaxed and rehydrated into water. Antigen retrieval was carried out by immersing the slides in 0.1M citrate buffer (pH 6) and microwaving for 10 minutes and slides were then left in the same buffer for a further 20 minutes followed by washing with tap water. Endogenous peroxidases in the tissue were blocked by incubating with 3% hydrogen peroxide for 5 minutes. To reduce non-specific binding, the slides were incubated with 3% bovine serum albumin + 3% normal horse serum for 30 minutes. Slides were then incubated overnight in a moist chamber with antibodies against pentraxin 3, VCAN core and hyaluronan binding protein (AMSBIO, 1/250). Negative controls consisted of 3% BSA in PBS. After thorough washing, all the slides were incubated with VECTASTAIN® Elite® ABC-Peroxidase Kit (R.T.U. Universal) for 30 minutes for each secondary and tertiary antibody (Vector Laboratories). Specimens

were then incubated with DAB (Sigma) for 5 minutes, washed well in tap water, nuclei were stained with hematoxylin for 1 minute and slides were mounted using Aquatex (VWR). Digital images were taken on a Nanozoomer (Hamamatsu).

Protein Quantification Assay

Protein concentrations were quantified using a Micro BCA^{TM} Protein Assay Kit, following the manufacturer's protocol. Standards for the calibration curve were prepared using serial dilutions of bovine serum albumin (BSA) (2 mg/mL). After incubation (1 hr, 60 °C), absorbance readings were measured (λ = 570 nm) using the Infinite M200 Pro Microplate reader (Tecan, 1112001880). Protein concentration was calculated using the BSA standard calibration curve.

Immunoprecipitation

A total of 100 µg of pooled human AV protein extracted in PBS was used for each immunoprecipitation, along with 10 ng of ITIH1 antibody. An equal amount of rabbit IgG was used as a negative control. The human AV protein extracts were maintained under non-denaturing conditions in PBS buffer. The protein–antibody–magnetic bead complexes were incubated at 4 °C for 16 hours with end-over-end mixing. After washing, the bound proteins were eluted under low pH conditions and preserved for subsequent proteomics analysis and Western blotting.

RNA Extraction and qPCR

Gene expression levels in normal, calcified human AV leaflets and mice AV leaflets were analyzed using RT-qPCR. Frozen samples were partially thawed and approximately 10 mg full-thickness fragments from the AV leaflets were obtained. AV leaflet samples were minced and homogenized in QIAzol (Invitrogen). 100 ng of RNA was reverse transcribed into cDNA. RT-qPCR was performed with SYBR™ Green PCR Master Mix using a ViiA 7 Real-Time PCR System (Thermo Fisher Scientific), the primer sequences used are shown below. Results were analyzed using the ΔΔCt method, where a housekeeping gene (*GAPDH* for human, *Gapdh* for mouse) is used for normalization of the target gene's expression levels.

Cell Culture

Murine VICs

Valve interstitial cells (VICs) were isolated from AVs of 5-6 weeks old male and female mice. Leaflets from 2-3 mice of the same genotype were pooled together and digested in warm DMEM/F-12 medium containing 1.0 mg/ml collagenase (type I) at 37℃ for 30 min on the shaker setting at 700rpm. After endothelial cells were removed by vortexing, the leaflets were further digested with a fresh solution of 4.5 mg/ml collagenase medium at 37 ℃ for 2 hrs on the shaker setting at 1400rpm. After repeated vortexing and aspirations to break up the tissue mass, the suspension was spun at 1,000 rpm for 5 min to precipitate cells. Cells were re-suspended and cultured in DMEM/F-12 growth medium, supplemented with 100 U/ml penicillin, 100 µg/ml streptomycin and 20% fetal bovine serum in an incubator with 5% CO_2 at 37℃ for the first 2 passages and then switched to 10% fetal bovine serum. Cells at passages 8-12 were used for experiments.

Human VICs

Human VICs were purchased from Innoprot and Lonza. Cells at passage 4-6 were used in experiments. Human VICs were first incubated in the MSCGM® Mesenchymal Stem Cell Growth Medium until they reached 80% confluency and then cultured for 21 days under four defined conditions. Control medium consisted of DMEM supplemented with 5% FBS.

Osteogenic medium was prepared by supplementing the control medium with 50 µg/mL (0.28 mM) ascorbic acid, 100 nM dexamethasone, and 10 mM β-glycerophosphate. TGFβ1 medium contained the control medium supplemented with 10 ng/mL TGFβ1. Chondrogenic medium was prepared by adding 1 mM sodium pyruvate, 0.1 mM ascorbic acid-2-phosphate, 1% ITS (25 mg/mL insulin, 25 mg/mL transferrin, 25 ng/mL sodium selenite), 1.25 mg/mL human serum albumin, 500 ng/mL BMP-2, and 10 ng/mL TGFβ1 in control medium. Cells were cultured with regular medium changes, and samples were collected on days 3, 7 and 14 for qPCR analysis. Parallel cultures were stained with Alizarin Red at day 21 to assess calcium deposition.

Single-cell RNA-sequencing Analysis

Publicly available single-cell RNA sequencing (scRNA-seq) datasets of aortic valves (AVs) from hypercholesterolemic mice[21] were retrieved from the Gene Expression Omnibus (GEO) database (accession code GSE180278). The pre-processed and normalized gene expression matrices, as provided by the original authors, were utilized directly for downstream analysis without further modification or additional data wrangling. All computational analyses were performed using R software (4.2.3). To infer and study cell-cell communication patterns, the CellChat R package (version 1.5.0)[22] was used. The CellChatDB.mouse database was assigned as the reference for ligand-receptor interactions. Overexpressed genes and interactions were identified using standard parameters (identifyOverExpressedGenes and identifyOverExpressedInteractions). Cell-cell communication probabilities were then computed using the computeCommunProb function with default settings, filtering out interactions present in fewer than 10 cells. Finally, the inferred communication networks were aggregated and visualized using CellChat's built-in functions.

Statistical Analysis

Statistical information for individual experiments is provided in the figure legends. Continuous data are presented as mean with standard error of the mean (SEM) or median with range, as described in the figure legends. Categorical variables were summarized using proportions. Statistical analyses were performed using Student’s unpaired t-test for two-group comparisons, one-way analysis of variance (ANOVA) followed by Tukey’s post hoc test for comparisons among multiple groups with a single variable, and two-way ANOVA followed by Sidak’s multiple-comparisons test for comparisons involving multiple variables. The data were tested for normality using the Shapiro-Wilk test before Student unpaired t-tests and ANOVA tests. Statistical analyses were generated using GraphPad Prism version 7.0 for Mac, GraphPad Software, San Diego, California USA, www.graphpad.com. A p-value of <0.05 was considered significant. The false discovery rate (FDR) for proteomics data was calculated using the Benjamini-Hochberg correction.

SUPPLEMENTAL FIGURES

SUPPLEMENTAL FIGURE 1

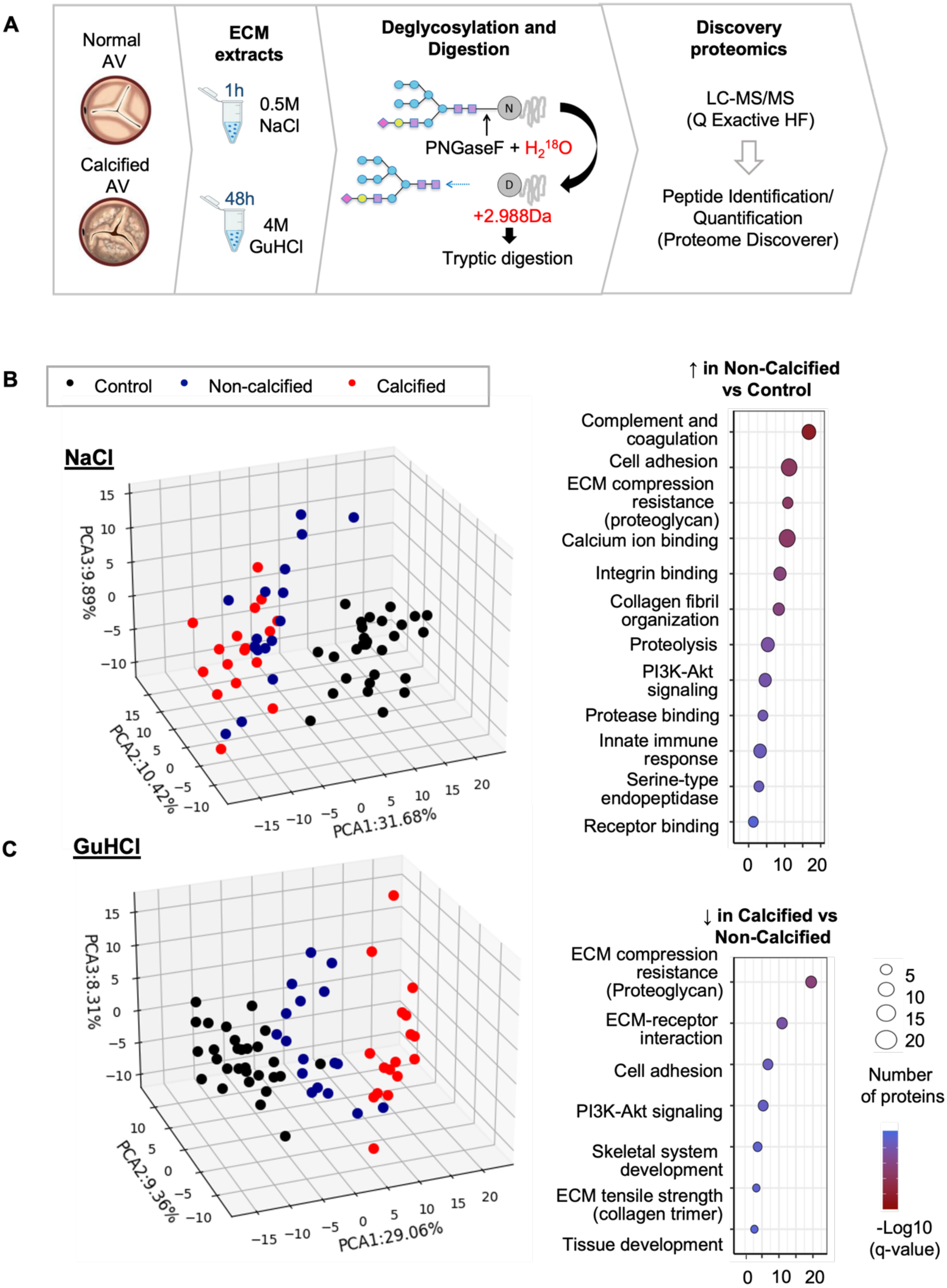

Figure S1. (A) The untargeted discovery proteomics workflow involves ECM protein extraction, deglycosylation, in-solution digestion, and subsequent liquid chromatography-tandem mass spectrometry analysis. Principal component analysis of the **(B)** NaCl and **(C)** GuHCl extracts reveals distinct clustering of control AVs, non-calcified and calcified areas of CAVD. Pathway enrichment analysis in (**B**) soluble and (**C**) core matrisome. ECM compression resistance (proteoglycans) emerged as significantly enriched pathways in both extracts.

SUPPLEMENTAL FIGURE 2

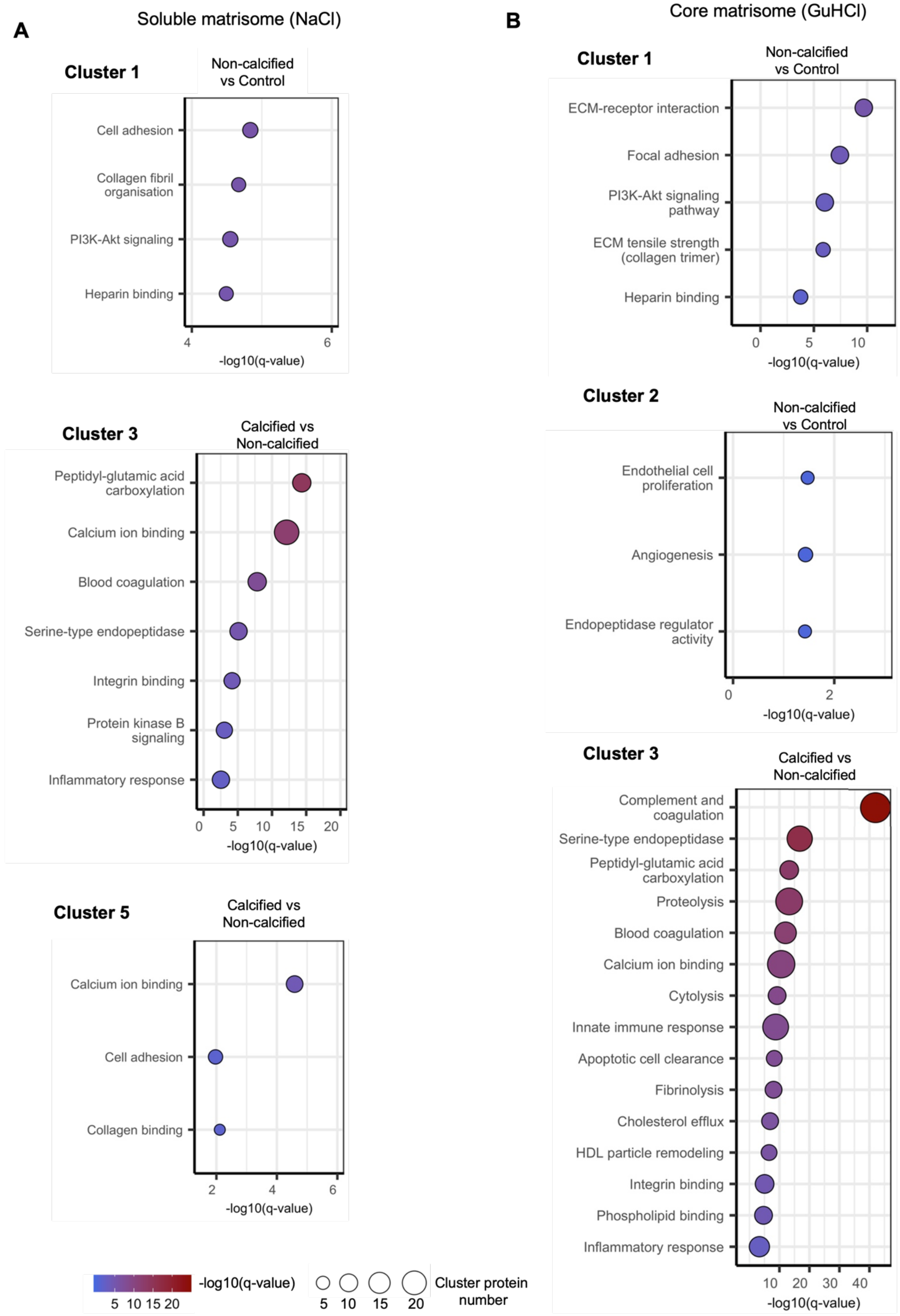

Figure S2. Pathway enrichment analysis in soluble and core matrisome. An overview of the top 15 pathways that are significantly enriched ($q<0.05$, q value is Benjamini-Hochberg corrected *p-value* from the enrichment analysis) in the selected clusters.

In the NaCl extract (soluble matrisome):

Cluster 1 shows upregulated pathways in CAVD non-calcified vs control.

Cluster 3 shows upregulated pathways in CAVD calcified vs CAVD non-calcified.

Cluster 4 shows downregulated pathways in CAVD calcified vs CAVD non-calcified.

Pathways downregulated in CAVD non-calcified vs control were non-significant.

In GuHCl extract (core matrisome):

Cluster 1 shows upregulated pathways in CAVD non-calcified vs control.

Cluster 2 shows downregulated pathways in CAVD non-calcified vs control.

Cluster 3 shows upregulated pathways in CAVD calcified vs non-calcified.

Cluster 4 shows downregulated pathways in CAVD calcified vs non-calcified.

The size of the circle indicates the number of proteins attributed to the pathway. Analysis was performed using DAVID Bioinformatics Resource.

SUPPLEMENTAL FIGURE 3

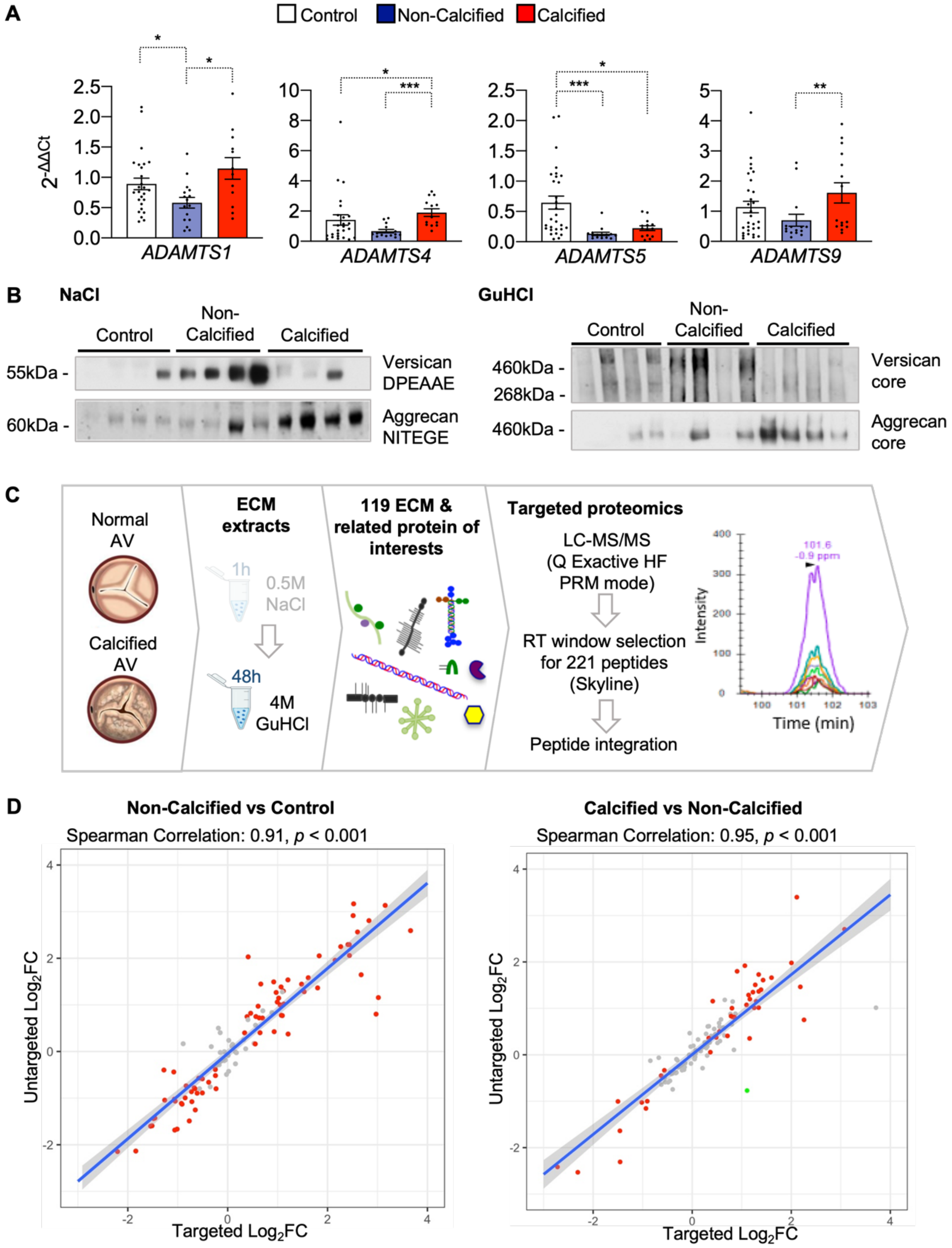

Figure S3. (A) Gene expression comparison of ADAMTS family members *ADAMTS1*, *ADAMTS4*, *ADAMTS5* and *ADAMTS9* among control, CAVD non-calcified and CAVD calcified groups are presented. Significance was calculated using the limma package for CAVD vs. controls and Student's paired t-test within CAVD groups (*p < 0.05, **p < 0.01). Data are shown as mean±SEM. **(B)** Immunoblot results stained for aggrecan, versican and corresponding neoepitopes among different groups in **(**left**)** soluble matrisome and **(**right**)** core matrisome. The human samples selected for immunoblots have comparable sex and age profiles, therefore sex and age correction were not applied to the subsequent densitometry analyses. **(C)** 210 peptides of 118 ECM proteins were used for targeted proteomics, using Skyline software and an optimized parallel reaction monitoring (PRM) method. A particular m/z was specified for individual peptides with specified fragments at specific retention times. **(D)** Strong correlation (R>0.9) of changes in non-calcified CAVD vs controls (left panel), and CAVD calcified vs non-calcified samples (right panel) between untargeted and targeted proteomics (GuHCl extracts). Red dots denote significantly changed proteins following the same trend.

SUPPLEMENTAL FIGURE 4

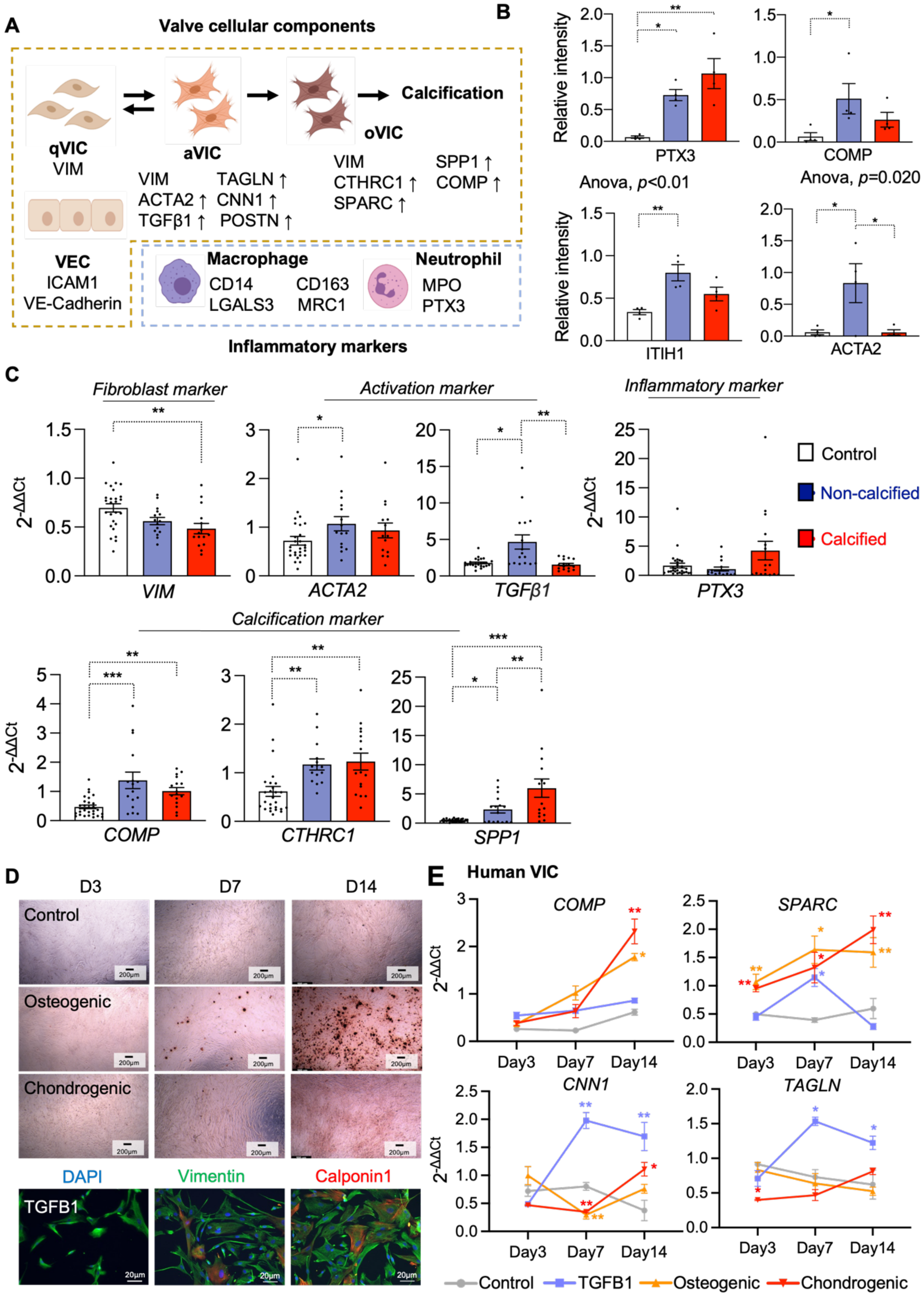

Figure S4. (A) Markers for key cell types in human AVs, adapted from previous literature.[43] VIC, valve interstitial cells; VEC, valve endothelial cells; qVICs, quiescent VICs; aVICs, activated myofibroblast-like VICs; oVICs, osteoblast-like VICs. ICAM1, intercellular adhesion molecule 1; VIM, vimentin; TAGLN, transgelin; ACTA2, smooth muscle cell actin; CNN1, calponin, CTHRC1, collagen triple helix repeat containing 1; SPP1, osteopontin; COMP, cartilage oligomeric matrix protein; SPARC, cysteine-rich acidic matrix-associated protein; CD14, cluster of differentiation 14; CD163, cluster of differentiation 163; LGALS3, galectin 3; MRC1, mannose receptor C-type 1; MPO, myeloperoxidase; PTX3, pentraxin 3. **(B)** Densitometry quantification of selected cell markers from immunoblots presented in **Figure 4B**. The human samples selected for immunoblots had comparable sex and age profiles, therefore sex and age correction were not applied to the subsequent densitometry analyses. Statistical significance was calculated using one-way ANOVA, * $p<0.05$, and ** $p<0.01$. **(C)** Relative gene expression of selected cell markers in human AVs. Significance was determined using the limma package (eBayes adjustment; $p<0.05$, Benjamini-Hochberg correction; fold changes (FC) >1.5) and Student's paired t-test for non-calcified vs. calcified regions in CAVD ($p<0.05$, Benjamini-Hochberg correction; FC > 1.5). * $p<0.05$, ** $p<0.01$, *** $p<0.001$. **(D)** Representative images of human VICs cultured under control, osteogenic, chondrogenic, or TGFβ1 conditions, showing time-dependent calcium deposition by Alizarin Red staining (top) and immunofluorescence staining for DAPI, vimentin, and calponin-1 (bottom). Osteogenic and chondrogenic medium induced calcium deposition over 14 days, while TGFβ1 promoted cytoskeletal remodeling and myofibroblast-like morphology. **(E)** Gene expression analysis of human VICs cultured in control, TGFβ1, osteogenic, or chondrogenic media. COMP, Cartilage oligomeric matrix protein; SPARC, osteonectin; CNN1, calponin-1; TAGLN, transgelin. Statistical significance across different time points was assessed using one-way ANOVA followed by Tukey's post hoc test, * $p<0.05$, and ** $p<0.01$. All data are shown as mean±SEM.

SUPPLEMENTAL FIGURE 5

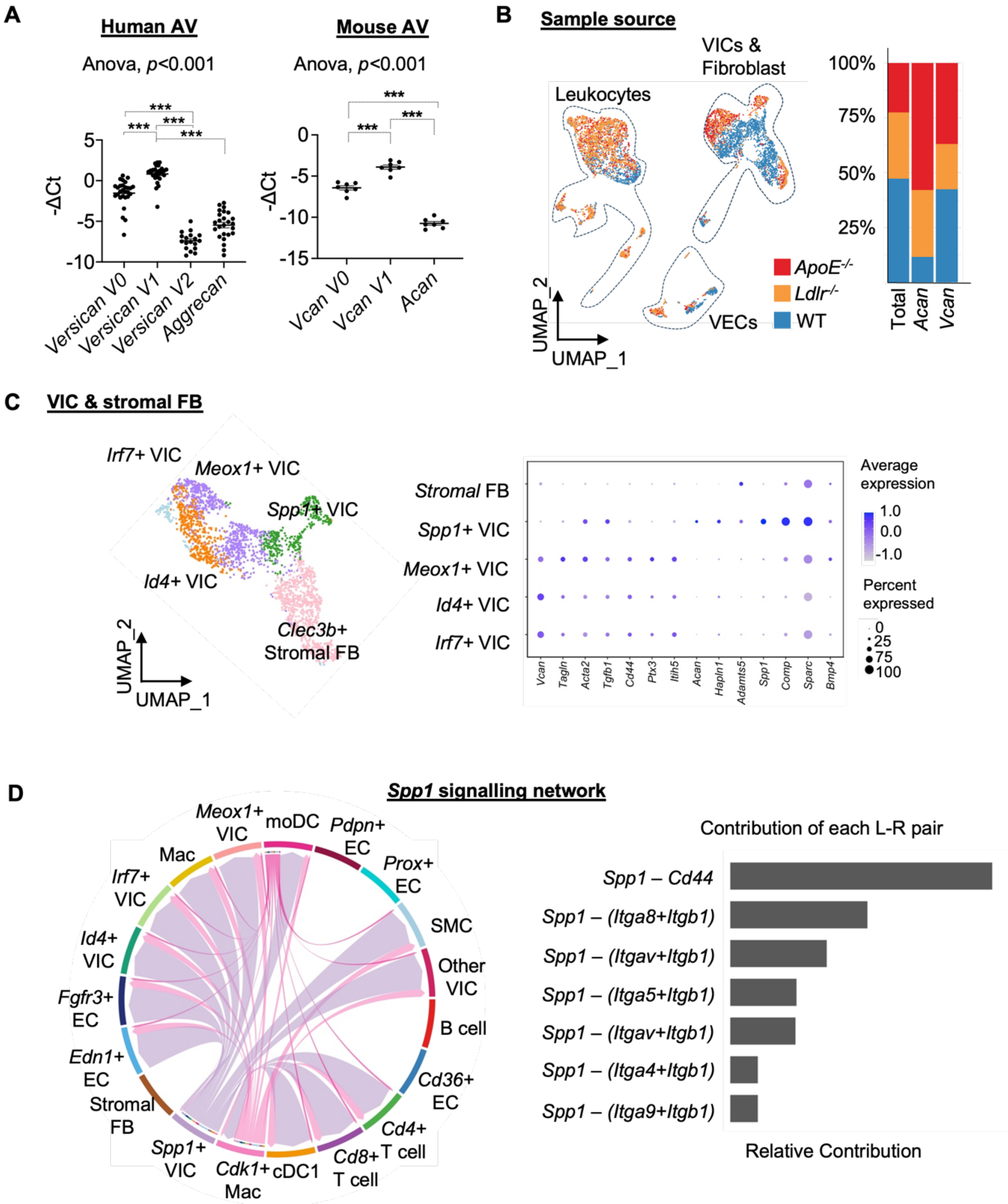

Figure S5. (A) Raw expression levels by qPCR of *VCAN* isoforms and *ACAN*. *Vcan V2* was not detected in mice. Statistical significance was calculated using one-way ANOVA (**p*<0.05, ****p*<0.001, mean±SEM). **(B)** UMAP plots showing the distribution of different cell populations derived from WT, *ApoE*$^{-/-}$, and *Ldlr*$^{-/-}$ mice, alongside stacked bar graphs illustrating genotype contributions to total, *Acan*$^{+}$, and *Vcan*$^{+}$ cell subsets. **(C)** ScRNA-seq data of murine AVs. Among the VIC subsets, *Spp1*+ VICs exhibited the lowest expression of versican (*Vcan*), but the highest expression of aggrecan (*Acan*), hyaluronan and proteoglycan link protein 1 (*Hapln1*) and cartilage oligomeric matrix protein (*Comp*). **(D)** Cell communication pattern mediated by the *Spp1* signaling pathway using the CellChat tool with a mouse scRNA-seq dataset (left panel).[20] Contribution of ligand-receptor interactions of the *Spp1* signaling pathway in cell-cell communications (right panel). CD44, hyaluronan receptor.

SUPPLEMENTAL FIGURE 6

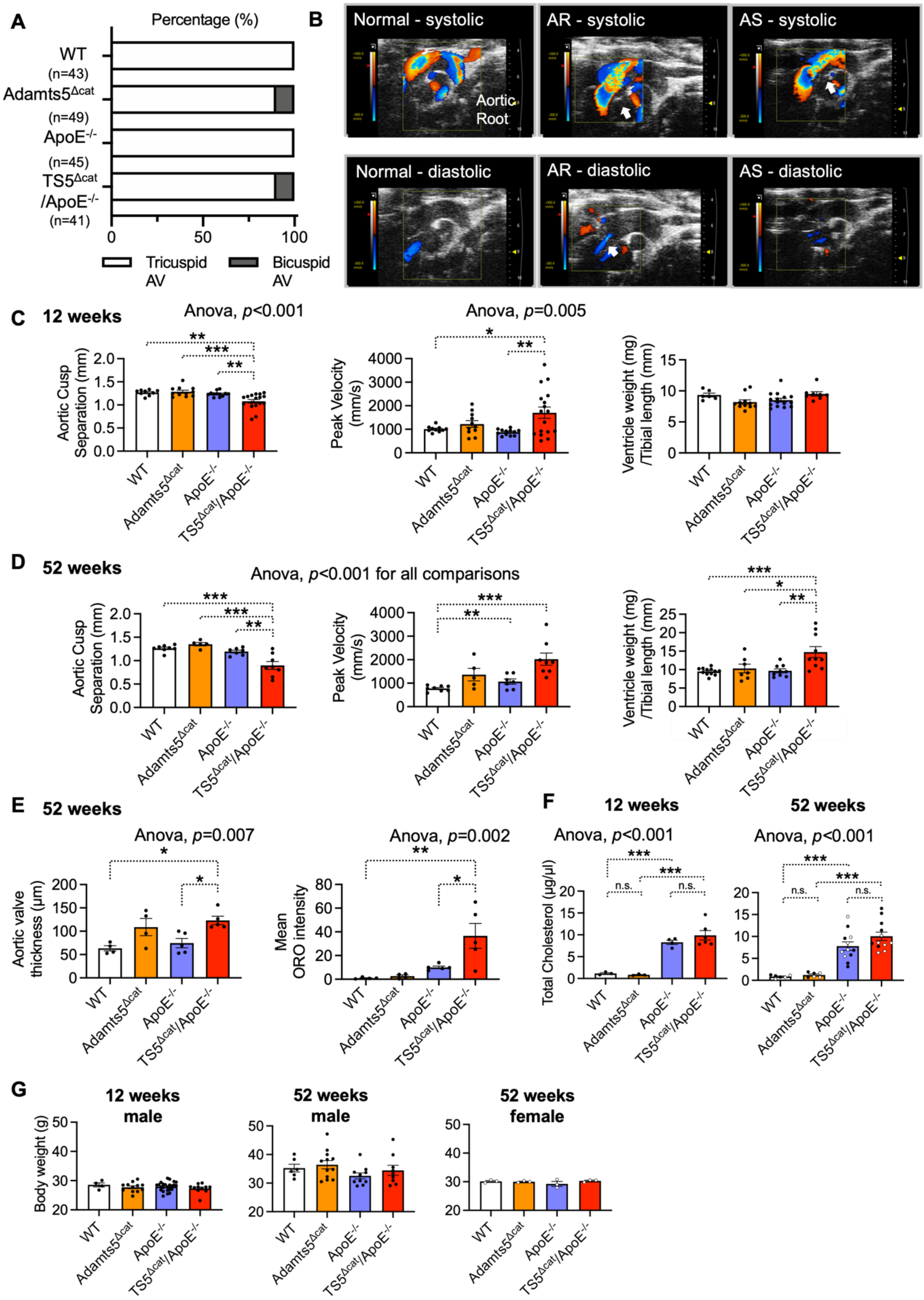

Figure S6. (A) Percentage of mice with different genotypes displaying bicuspid AVs. *Adamts5*$^{\Delta cat}$*/ApoE*$^{-/-}$ abbreviated as *TS5*$^{\Delta cat}$*/ApoE*$^{-/-}$. **(B)** Representative color Doppler images in aortic root section, revealing the presence of aortic regurgitation (AR) or aortic stenosis (AS). Normal color flow pattern (left) in wildtype mice. AR backflow (middle) during diastole and AS jet during systole (right). **(C)** Aortic cusp separation distance, mean transvalvular blood velocity, and ventricle weight in 12-week-old male mice (WT - *Adamts5*$^{+/+}$ littermates, n=7; *Adamts5*$^{\Delta cat}$, n=10; *ApoE*$^{-/-}$, n=11; *ADAMTS5*$^{\Delta cat}$*/ApoE*$^{-/-}$, n=17) and **(D)** 52-week-old male mice (WT, n=8; *Adamts5*$^{\Delta cat}$, n=5; *ApoE*$^{-/-}$ n=7; *Adamts5*$^{\Delta cat}$*/ApoE*$^{-/-}$ n=8); Statistical significance was calculated using one-way ANOVA, * denotes p<0.05 , ** p<0.01, *** p<0.001. **(E)** Aortic valve thickness quantification and Oil Red O staining intensity quantification of AV leaflets from mice with different genotypes. Representative image can be found in **Figure 5F**. **(F)** Serum total cholesterol levels in WT, Adamts5$^{\Delta cat}$, ApoE$^{-/-}$, and TS5$^{\Delta cat}$/ApoE$^{-/-}$ mice aged 12 and 52 weeks. Statistical significance was calculated using one-way ANOVA with Tukey's post hoc test (** denotes p<0.01). Comparisons between ApoE$^{-/-}$ and Adamts5$^{\Delta cat}$ are not shown. **(G)** Body weights of WT, Adamts5$^{\Delta cat}$, ApoE$^{-/-}$, and TS5$^{\Delta cat}$/ApoE$^{-/-}$ mice aged 12 and 52 weeks. Differences between groups were not statistically significant as tested by one-way ANOVA analysis. Black dots denote male mice, while white dots denote females. All data are shown as mean±SEM.

SUPPLEMENTAL FIGURE 7

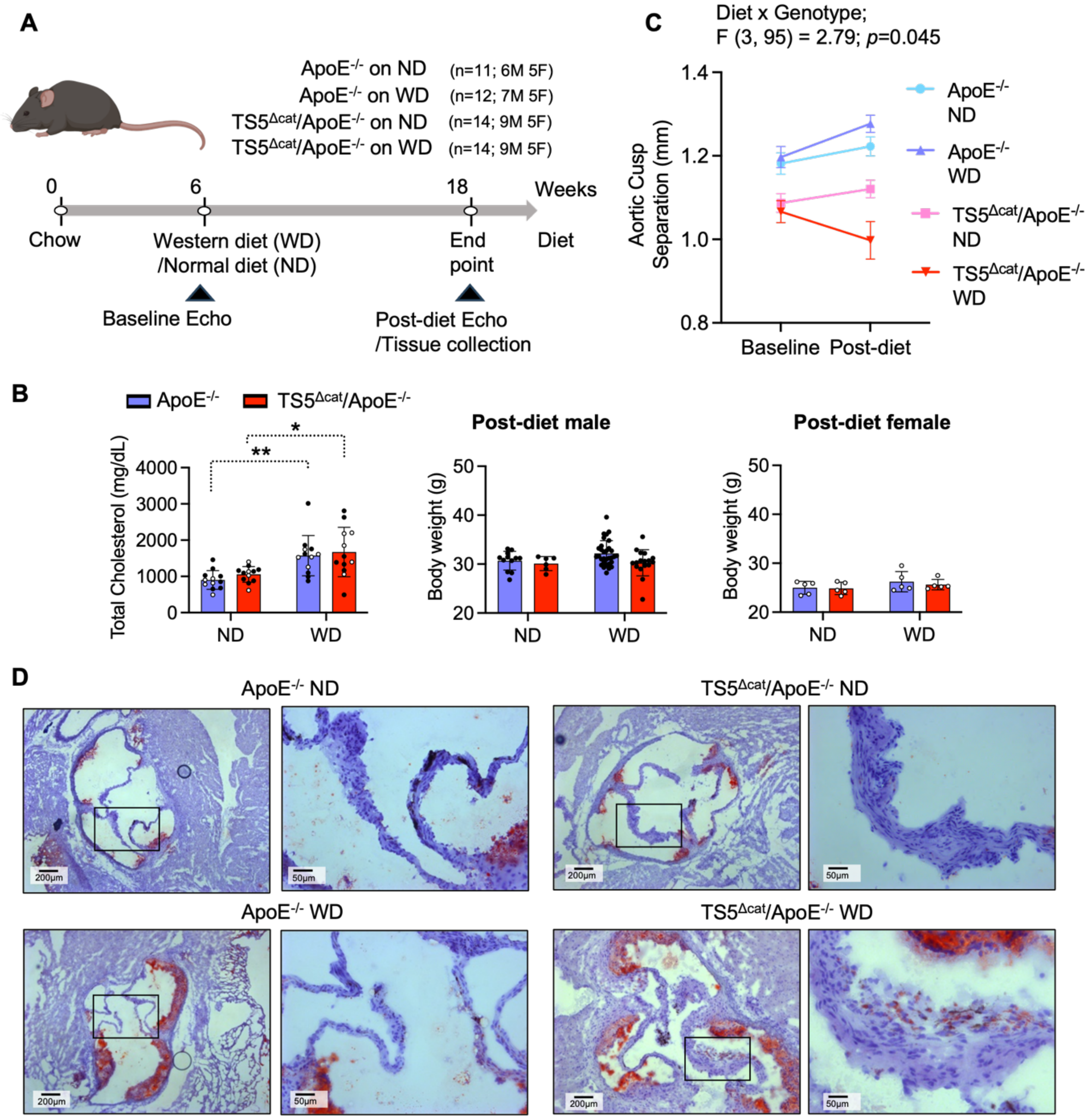

Figure S7. (A) Aortic valve (AV) function in *ApoE*$^{-/-}$ and *TS5*$^{\Delta cat}$*/ApoE*$^{-/-}$ mice at baseline and post diet was assessed by echocardiography. **(B)** (left) Post-diet serum total cholesterol levels in ApoE$^{-/-}$ on normal diet (ND, n=12; 7M, 5F) or Western diet (WD, n=12; 7M, 5F) and *TS5*$^{\Delta cat}$*/ApoE*$^{-/-}$ mice on ND (n=12; 8M, 4F) or WD (n=12; 7M, 5F). Black dots denote male mice, white dots denote females. Interaction effects of diet and genotype were evaluated using two-way ANOVA, followed by Sidak's post hoc pairwise comparison; only comparisons between groups differing by one variable are shown (* denotes $p<0.05$, ** denotes $p<0.01$). Data are shown as mean±SEM. (middle-right) Post-diet body weights of *ApoE*$^{-/-}$ mice on ND (n=17; 12M, 5F) or WD (n= 35; 30M, 5F), and *TS5*$^{\Delta cat}$*/ApoE*$^{-/-}$ on ND (n=11; 6M, 5F) or WD (n=22; 17M, 5F). Weight data from past experiments are used and combined. Differences between groups were not statistically significant as tested by two-way ANOVA analysis. **(C)** Aortic cusp separation distance of *ApoE*$^{-/-}$ and *TS5*$^{\Delta cat}$*/ApoE*$^{-/-}$ mice at post-diet level. Interaction effects of diet and genotype were evaluated using two-way ANOVA, followed by Sidak's post hoc pairwise comparison; only comparisons between groups differing by one variable are shown. (D) Representative Oil Red O-stained images of aortic roots from *ApoE*$^{-/-}$ and *TS5*$^{\Delta cat}$*/ApoE*$^{-/-}$ mice after 12 weeks of ND or WD feeding.

SUPPLEMENTAL FIGURE 8

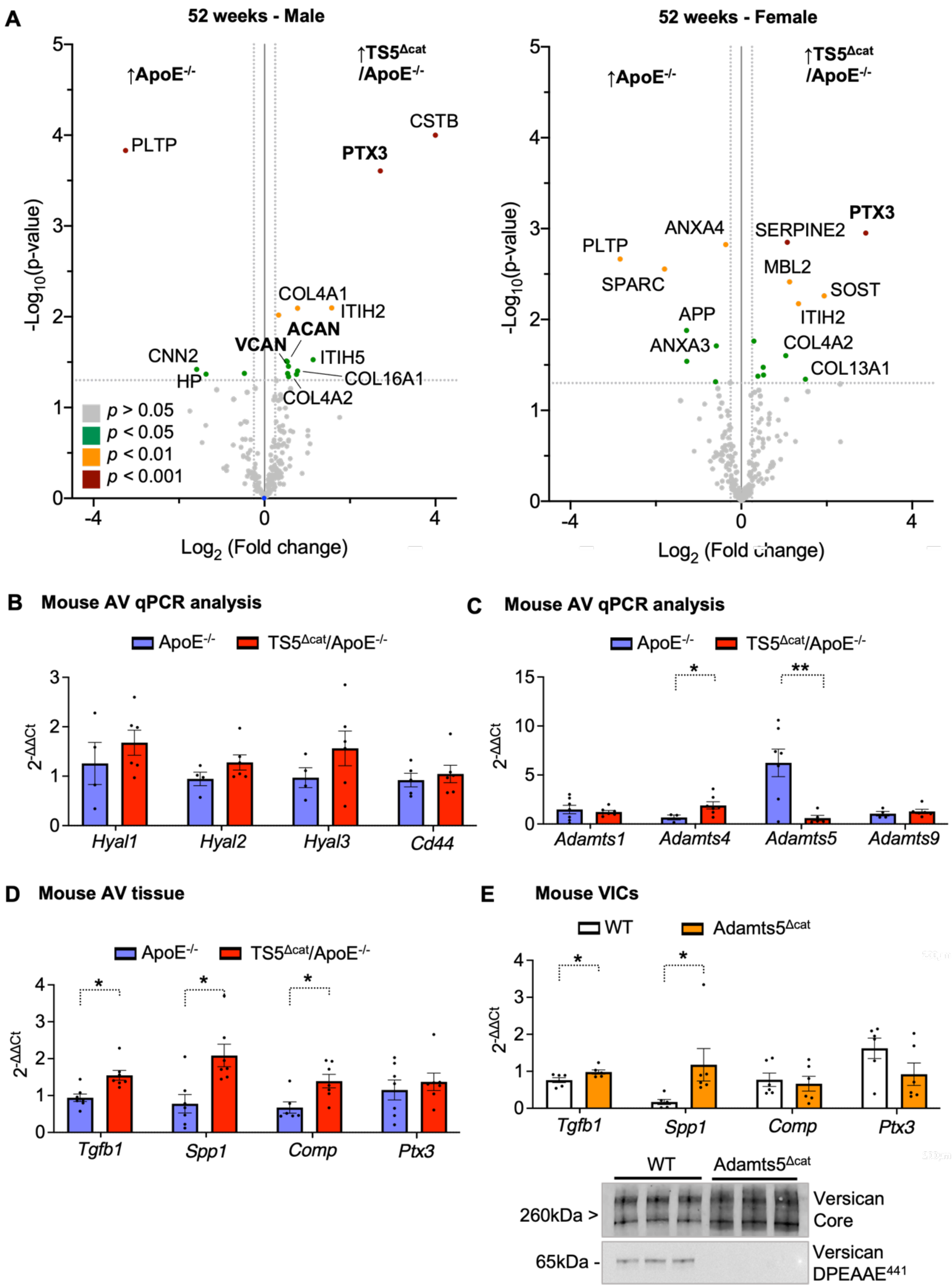

Figure S8. (A) Volcano plots showing ECM proteins dysregulated in aortic valve leaflets (AVs) in (left) male and (right) female 52-week-old *ApoE*$^{-/-}$ (n=7; 4M, 3F) and *TS5*$^{\Delta cat}$*/ApoE*$^{-/-}$ (n=7; 4M, 3F) mice. Comparisons between *ApoE*$^{-/-}$ vs *TS5*$^{\Delta cat}$*/ApoE*$^{-/-}$ mice are shown. Statistical significance was calculated using unpaired t-test, and proteins that reached nominal statistical significance (p<0.05) were labelled on the volcano plot. Different color dots represent changes with different ranges of p-values. **(B)** Relative gene expression of *Hyal1*, *Hyal2*, *Hyal3* and *Cd44* in AV tissue from *ApoE*$^{-/-}$ and *Adamts5*$^{\Delta cat}$*/ApoE*$^{-/-}$ mice. **(C)** Relative gene expression of *Adamts* family members with hyalectanase ability in AV tissue from *ApoE*$^{-/-}$ and *Adamts5*$^{\Delta cat}$*/ApoE*$^{-/-}$ mice. **(D-E)** Relative gene expression of *Tgfb1*, *Spp1*, *Comp* and *Ptx3* in AV tissue from **(D)** *ApoE*$^{-/-}$ and *Adamts5*$^{\Delta cat}$*/ApoE*$^{-/-}$ mice and VICs isolated from **(E)** wildtype (WT) and *Adamts5*$^{\Delta cat}$ mice. Statistical significance was calculated using a Student's unpaired t-test. * *p*<0.05. Data are means±SEM. *Adamts5*$^{\Delta cat}$*/ApoE*$^{-/-}$ abbreviated as *TS5*$^{\Delta cat}$*/ApoE*$^{-/-}$.

SUPPLEMENTAL FIGURE 9

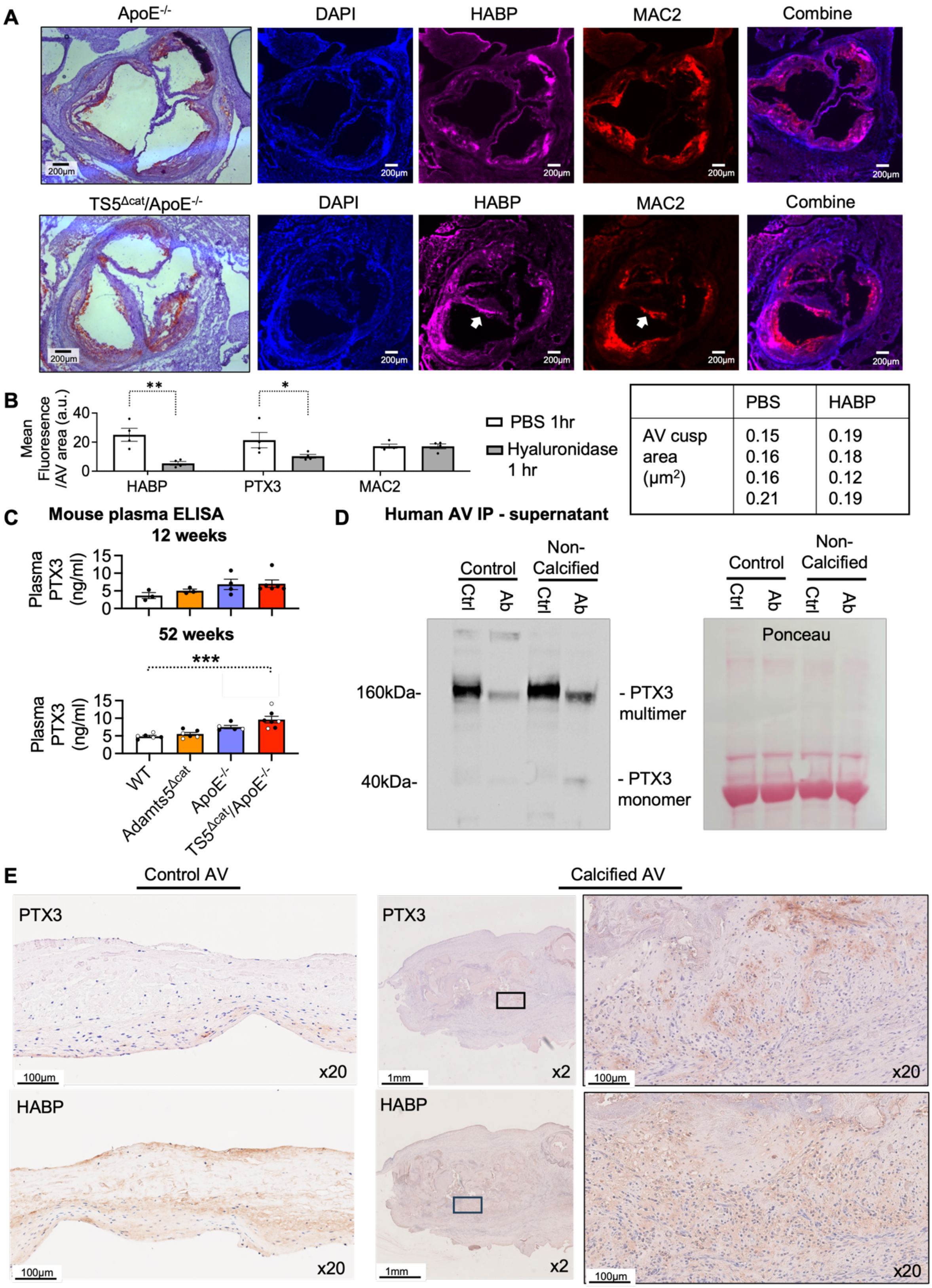

Figure S9. (A) Representative images showing Oil Red O staining and immunofluorescence staining for MAC2 and hyaluronan (using the HA-binding protein - HABP) in *ApoE*$^{-/-}$ and *TS5*$^{\Delta cat}$/*ApoE*$^{-/-}$ mice. Nuclei are counterstained with DAPI. White arrow indicates co-staining of MAC2 and HABP in lipid-rich areas on AV cusps from *TS5*$^{\Delta cat}$/*ApoE*$^{-/-}$ mice. Total AV cusp areas are listed for complementary information. **(B)** Quantification of immunofluorescence staining intensity of PTX3, hyaluronan and MAC2 in murine AVs with 1hr hyaluronidase (HAdase) or 1hr PBS treatment. Corresponding representative images can be found in **Figure 6D.** Statistical significance was calculated using paired t-test. * denotes $p<0.05$ and ** denotes $p<0.01$. **(C)** Plasma PTX3 levels measured by ELISA in 12- and 52-week-old mice**.** Statistical significance was calculated using one-way ANOVA, * denotes $p<0.05$. Data are mean±SEM. **(D)** A total of 100 µg of AV protein extracts from pooled human control and non-calcified AS samples was immunoprecipitated using an antibody against inter-α-trypsin inhibitor heavy chain 1 (ITIH1 ab, labelled as ab in the figure). The immunoblot uses the supernatant from the immunoprecipitation (IP), loaded under non-reducing conditions (without any reducing reagent to break the disulfide bonds). The corresponding IP eluate is shown in **Figure 6F**. **(E)** Immunoperoxidase images showing staining for pentraxin 3 (PTX3) and hyaluronan in human control and calcified AVs. All sections were counterstained with hematoxylin to visualize nuclei.

SUPPLEMENTAL FIGURE 10

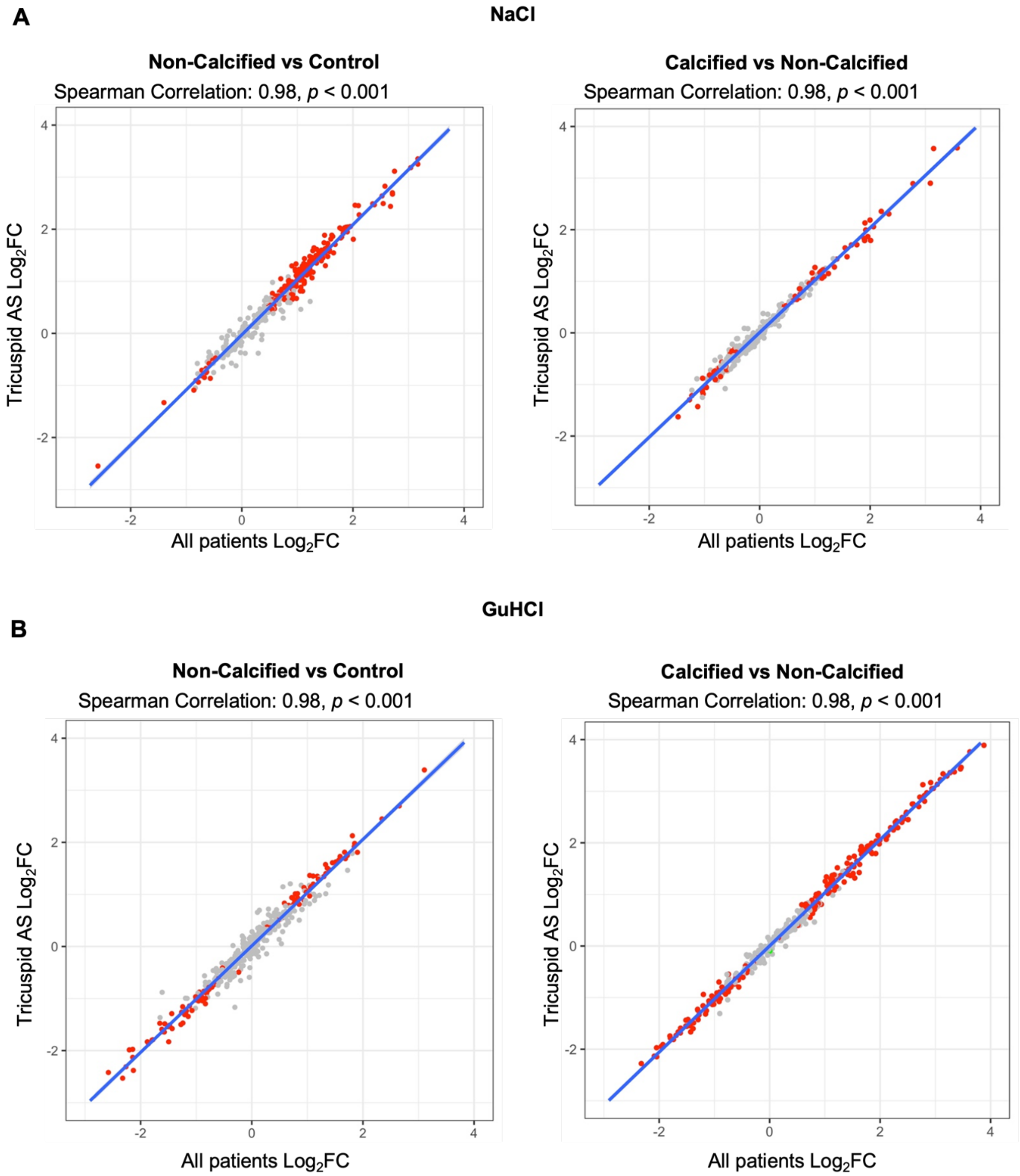

Figure S10. Strong correlation (R>0.98) was observed for protein abundance changes between all patients and tricuspid AV patients only, in both comparisons: non-calcified CAVD vs controls (left panel) and CAVD calcified vs non-calcified samples (right panel), for **(A)** NaCl and **(B)** GuHCl extracts. Red dots indicate significantly changed proteins following the same trend in both datasets. The high correlation coefficients (R > 0.98) between the full cohort and the TAV-only subset indicate that excluding the few bicuspid cases (n=2) has minimal impact on the overall differential expression patterns. This suggests that the bicuspid valve samples are largely consistent with the group-level variance and do not introduce subgroup-specific substantial deviation.

SUPPLEMENTAL TABLES

Supplemental Tables S1-11 are provided as datasets in Excel.

Supplemental Table S12. Major Resource Tables.

**Primary antibodies**				
**Reactivity**	**Host**	**Supplier**	**Product CAS**	**Dilution**
Anti-GAPDH	Rabbit	Cell Signalling	14C10	1:2000 for WB
Anti-DCN	Rabbit	Santa Cruz	sc-22753	1:100 for WB
Anti-ACAN neo	Rabbit	Thermo Scientific	PA1-1746	1:830 for WB
Anti-ACAN core	Rabbit	Sigma-Aldrich	AB1031	1:500 for WB<br>1:250 for peroxide staining
Anti-VCAN neo	Rabbit	Abcam	Ab19345	1:2000 for WB,<br>1:100 for IF
Anti-VCAN core	Rabbit	Abcam	AB270444	1:1000 for WB
Anti-VCAN core	Rabbit	EMD Millipore	AB1033	1:100 for peroxide staining
Anti-HAPLN1	Goat	Abcam	AF2608	1:500 for WB
Anti-VIM	Mouse	Abcam	ab8069	1:1000 for WB
Anti-PTX3	Rabbit	Abcam	ab125007	1:1000 for WB
Anti-PTX3	Rat	Abcam	ab90806	1:1000 for WB
Anti-COMP	Rat	Abcam	ab11056	1:100 for WB
Anti-ACTA2	Rabbit	Abcam	ab5694	1:1000 for WB
Anti-PTX3	Rabbit	Abcam	ab190838	1:100 for IF<br>1:75 for peroxide staining
Anti-PTX3	Rat	Abcam	ab90806	1:1000 for WB
Anti-ITIH1	Rabbit	EMD Millipore	ZRB1737	10ng ab : 100μg protein sample for IP
Anti-ITIH1	Rabbit	Abcam	ab110822	1:1000 for WB
Anti-MAC2	Rat	Biolegend	125401	1:100 for IF
Biotinylated Hyaluronan Binding Protein	Streptavidin	AMSBIO	AMS.HKD-BC41	1:1000 for WB<br>1:200 for IF<br>1:250 for peroxide staining
Rabbit IgG, Control Antibody	Rabbit	Vector Lab	I-1000-5	Match the primary antibody concentration
Rat IgG, Control Antibody	Rat	Abcam	ab37361	Match the primary antibody concentration

**Secondary antibodies**				
**Reactivity**	**Host**	**Supplier**	**Product CAS**	**Dilution**
HRP Anti-Rabbit IgG	Mouse	Jackson ImmunoResearch	211-032-171	1:5000

HRP Anti-Mouse IgG	Goat	Jackson ImmunoResearch	115-035-174	1:5000
HRP Anti-Goat IgG	Mouse	Jackson ImmunoResearch	205-032-176	1:5000
HRP Anti-Rat IgG	Goat	Jackson ImmunoResearch	112-005-003	1:5000
IRDye® 680RD Goat anti-Rabbit IgG (H + L)	Goat	LI-COR Biosciences	26-68071	1:15000
IRDye® 800CW Goat anti-Rabbit IgG (H + L)	Goat	LI-COR Biosciences	926-32211	1:15000
IRDye® 680RD Donkey anti-Goat IgG (H + L)	Donkey	LI-COR Biosciences	926-32214	1:15000
Goat anti-Rabbit IgG (H+L), Alexa Fluor 546	Goat	Thermo	A-11035	1:1000
Streptavidin conjugated Alexa Fluor 546	n/a	Life Technology	S11225	1:400
**Electrophoresis markers**				
Novex™ Sharp Pre-Stained Protein Standard		Thermo Scientific	LC5800	
HiMark™ Pre-stained Protein Standard		Thermo Scientific	LC5699	
Chameleon Duo Pre-stained Protein Ladder		LI-COR Biosciences	928-60000	
Precision Plus Protein™ Unstained Protein Standards		Bio-techne	1610363	

Materials

**Name**	**Vendor**	**Catalog #**
**1.Chemicals**		
Acetonitrile	Fisher Scientific	10001334
Acetonitrile, 80% (aq), with 0.1% Formic Acid	Fisher Scientific	15431423
Alizarin-Red Staining Solution	Merck Millipore	TMS-008-C
Agar	Sigma-Aldrich	A1296
Beta-glycerophosphate	Sigma-Aldrich	G9422
BMP2	Bio-techne	355-BEC-025/CF
Chloroform	Sigma-Aldrich	C2432
Cocktail of proteinase inhibitor	Sigma-Aldrich	P8340
Collagenase, Type I, powder	Gibco	17018029
Dexamethasone	Sigma-Aldrich	D4902
DMEM/F-12	Gibco	11574546
EDTA	Sigma-Aldrich	E5134
EGTA	Sigma-Aldrich	E3889
Ethanol	Fisher Scientific	BP2818-500
Foetal Bovine Serum (FBS)	Gibco	10500-064

Gelatin	Sigma	G1393
Glycine	Fisher Bioreagents	BP381-1
GoTaq Hot Start Polymerase	Promega	D6001
Guanidine hydrochloride	Sigma-Aldrich	G3272
GlutaMAX™ Supplement	Thermo Scientific	35050061
$H_2^{18}O$	Taiyo Nippon Sanso	FO3-0027
Hyaluronidase from bovine testes	Sigma-Aldrich	H3506
Insulin-Transferrin-Selenium (ITS-G) (100X)	Gibco	41400045
L-ascorbic acid	Sigma-Aldrich	A4544
Iodoacetamide	Sigma-Aldrich	A3221
Methanol	Fisher Chemical	M/4056/17
Micro BCATM Protein Assay Kit	Thermo Scientific	23235
MSCGM® Mesenchymal Stem Cell Growth Medium BulletKit®	Lonza	PT-3001
NuPAGE MOPS SDS Running Buffer (20X)	Life Technologies	NP0001-02
Oil Red O staining	Sigma-Aldrich	O9755
Pen-Strep	Gibco	15140-122
Phosphate-buffered saline (PBS)	10X Severn Biotech	Ltd 20-7400-10
PhosSTOP	Roche	4906837001
Ponceau	Sigma	P7170-1L
Qiazol Lysis Reagent	Qiagen	79306
Sodium Acetate	Sigma-Aldrich	S2889
Sodium Azide	Sigma-Aldrich	S2002
Sodium Chloride	Sigma-Aldrich	S9888
Sodium Dodecyl Sulfate, SDS	Genomic Solution	80-0175
Sodium Pyruvate	Sigma-Aldrich	P5280
Super Script VILO MasterMix	Invitrogen	11755050
SYBR™ Green PCR Master Mix	Applied Biosystems	4309155
TGFbeta1	Bio-techne	240-B-002/CF
Triethylammonium Bicarbonate (TEAB)	Sigma-Aldrich	T7408
Trifluoroacetic Acid (TFA)	Thermo Scientific	85183
Thiourea	Supelco	107979
Trypsin-EDTA (0.05%)	Gibco	25300-054
Tris-hydrochloride (Tris-HCl)	Sigma-Aldrich	T3253
Triton X-100	Sigma-Aldrich	X100
Trizma base	Sigma-Aldrich	T1503
Tween-20	Sigma-Aldrich	P7949

Urea	Fisher Scientific	11337898
Water, with 0.1% Formic Acid	Fisher Scientific	10188164
**2. Enzymes**		
α2-3,6,8,9-neuraminidase (Sialidase)	EMD Millipore	362280 (KP0012)
β1,4-galactosidase	EMD Millipore	362280 (KP0004)
β-N-acetylglucosaminidase	EMD Millipore	362280 (KP0013)
Chondroitinase ABC	Sigma-Aldrich	C3667
Endo-α-N-acetylgalactosaminidase (O-glycosidase)	EMD Millipore	362280 (KP0011)
Heparinase II	Sigma-Aldrich	H6512
Keratanase	Sigma-Aldrich	G6920
PNGaseF (N-Glycosidase)	EMD Millipore	362280 (KP0001)
Trypsin, MS Grade	Thermo Scientific	90057
**3. Reagent Kits**		
Avidin/Biotin Blocking Kit	Vector Lab	SP-2001
Cholesterol Assay Kit	Abcam	ab65390
Dynabeads™ Protein G for Immunoprecipitation	Thermo Scientific	10003D
Fractionation Kit	Thermo Scientific	84868
High-Capacity RNA-to-cDNA™ Kit	Applied Biosystems	4368814
Normal Horse Serum Blocking Solution	Vector Lab	S-2000-20
Mouse Pentraxin 3/TSG-14 ELISA Kit	Bio-techne	MPTX30
Micro BCATM Protein Assay Kit	Thermo Scientific	23235
NuPAGE 4-12% Bis-Tris gels	Thermo Scientific	NP0336BOX
Pierce Bradford Protein Assay Kit	Thermo Scientific	23200
Pierce™ Colorimetric Protease Assay Kit	Fisher Scientific	23263
TMTpro™ 16plex Label Reagent Set	Thermo Scientific	A44520
VECTASTAIN® Elite® ABC-HRP Kit, Peroxidase, R.T.U. (Universal)	Vector Lab	PK-7200

Primers

1. Human

**Target**	**FWD**	**RVS**
ACAN	TCGAGGACAGCGAGGCC	TCGAGGGTGTAGCGTGTAGAGA
ACTA2	CCGGGACTAAGACGGGAATC	TTGTCACACACCAAGGCAGT
ADAMTS1	GCACTGCAAGGCGTAGGAC	AAGCATGGTTTCCACATAGCG
ADAMTS4	CAAGGTCCCATGTGCAACGT	CATCTGCCACCACCAGTGTCT
ADAMTS5	TGTCCTGCCAGCGGATGT	ACGGAATTACTGTACAGCCTACA
ADAMTS9	CAATACCAACTCCGAGCACA	TGGGGTTTGTTTTGTTCCTC
COMP	GGAGATGCTTGTGACAGCGATC	TGAGTCCTCCTGGGCACTGTTA

CTHRC1	CAATGGCATTCCGGGTACAC	GTACACTCCGCAATTTTCCCA
GAPDH	GGCAAATTCCATGGCACCG	AGCATCGCCCCACTTGATTT
HAPLN1	TTGAGAGCATCCGAACTCCTG	GGGCCATTTTCTGCTTGGATG
POSTN	CAGCAAACCACCTTCACGGATC	TTAAGGAGGCGCTGAACCATGC
PTX3	GGGACAAGCTCTTCATCATGCT	GTCGTCCGTGGCTTGCA
SPARC	GCGGAAAATCCCTGCCAGAA	GGCAGGAAGAGTCGAAGGTC
SPP1	ACTCGAACGACTCTGATGATGT	GTCAGGTCTGCGAAACTTCTTA
TAGLN	AAACCCACCCTCTCAGTCAG	GACCCTTGTTGGCCATGTCT
TGFB1	TACCTGAACCCGTGTTGCTCTC	GTTGCTGAGGTATCGCCAGGAA
VCAN v0	CCAGCAAGCACAAAATTTCA	TTGGATGACCAATTACACTCAAA
VCAN v1	TGAGAACCAGACAGGCTTCC	TTGGATGACCAATTACACTCAAA
VCAN v2	CCAGCAAGCACAAAATTTCA	GTTAAGGCACGGGTTCATTTT
VIM	TGTCCAAATCGATGTGGATGTTTC	TTGTACCATTCTTCTGCCTCCTG
**2. Murine**		
**Target**	**FWD**	**RVS**
Acan	GATCCAGAACCTTCGCTCCA	TGCTCGATCAAAGTCCAGGG
Adamts1	ATGGACACGGGGAATGTTTGA	GTGCTTGGATTCCTCTCCGAA
Adamts4	TTGTGGAAGATGATGGGCTCC	CCCCCTGCCCATTCAAGTTA
Adamts5	TGCCCACCCAATGGTAAATCTT	CAGGACACCTGCATATTTGGGA
Adamts9	TGGTCAGAGTGCTTGGTCAC	GCCTCAGGGTCACACATTCT
Gapdh	AGGTCGGTGTGAACGGATTTG	GGGGTCGTTGATGGCAACA
Cd44	CGGAACCACAGCCTCCTTTCAA	TGCCATCCGTTCTGAAACCACG
Hyal1	GGCTTCCCTGACTGCTACAA	ACCCTAGCTGGTCGTTCTGA
Hyal2	TGAGCCAGGTGGACCTTATC	CTTGAAGCGTCTTCCGAGTC
Hyal3	CTTCCGTGTAGCCCTTTTGGAG	CTCCAATAGTCTGCATCAGGTCG
Hapln1	TACTGCTGTGGTGGCATTGGAG	GGTCAAAGGAAGCAATCACTGCG
Has1	GGGTCAGCTTCTTGAGCAGT	ACCACTGATGCAGGACACAC
Has2	CCCTGGGATTAAAGTCGTGA	TTGTCCCTGCCCATAACTTC
Has3	CGGGTGAAGGAGAGACAGAG	GCATGATGCATGAGAAGGTG
Ptx3	CCTGCGATCCTGCTTTGTG	GGTGGGATGAAGTCCATTGTC
Comp	GTGCCCAACTTTGACCAGAGTG	ACAGGCATCACCCACAAAGTCG
Spp1	CTGGCTGAATTCTGAGGGACT	TTCTGTGGCGCAAGGAGATT
Tgfb1	TGACGTCACTGGAGTTGTACGG	GGTTCATGTCATGGATGGTGC
Vcan V0	AAGACAGGTCGATTGAGTGATATG	CGGGTAAAATTTCAGCAAACA
Vcan V1	GAGGTCTACTTGGGGTGAGAAC	TCACTCAATCGACGTTTAAAGCA
Vcan V2	AGAGAAAACAAGACAGGACCTGAT	CAGGTGCACACATAGGAAGTCT
