# Supplementary material for: Aortic Stenosis Hyalectan Remodeling Revealed by Proteomics and Glycoproteomics
Source: Arterioscler Thromb Vasc Biol. 2026 Jul 16;46(9):e323867. doi: 10.1161/ATVBAHA.126.323867 (PMC13506200; doi:10.1161/ATVBAHA.126.323867)

Negative control for Figure S4D

Mouse IgG | Rabbit IgG | Combined with DAPI

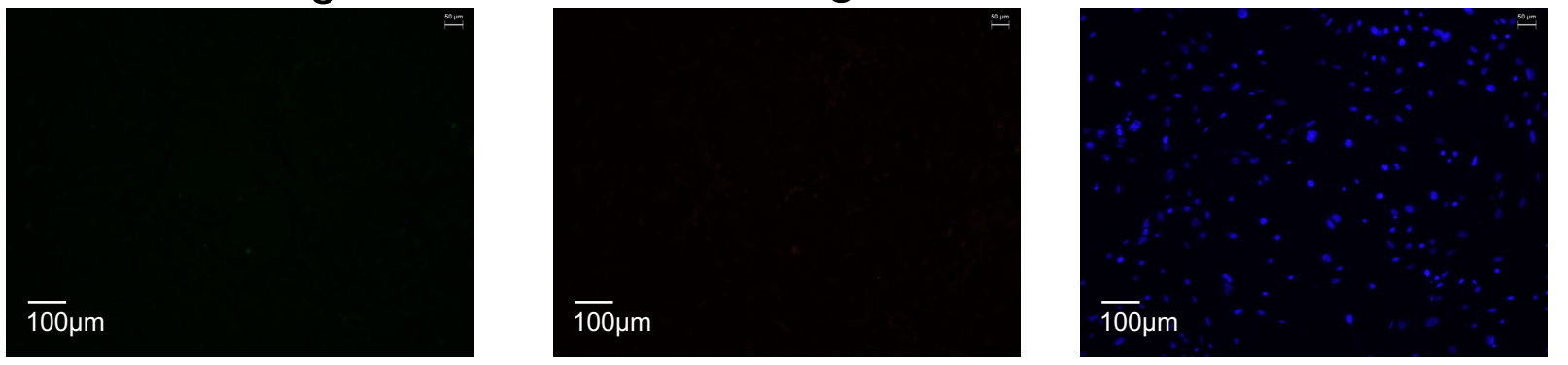

Mouse IgG (Vimentin control)
Rabbit IgG (Calponin1 control) were used as primary ab

All ORO staining images used in quantification in Figure S6D

Adamts5$^{+/+}$

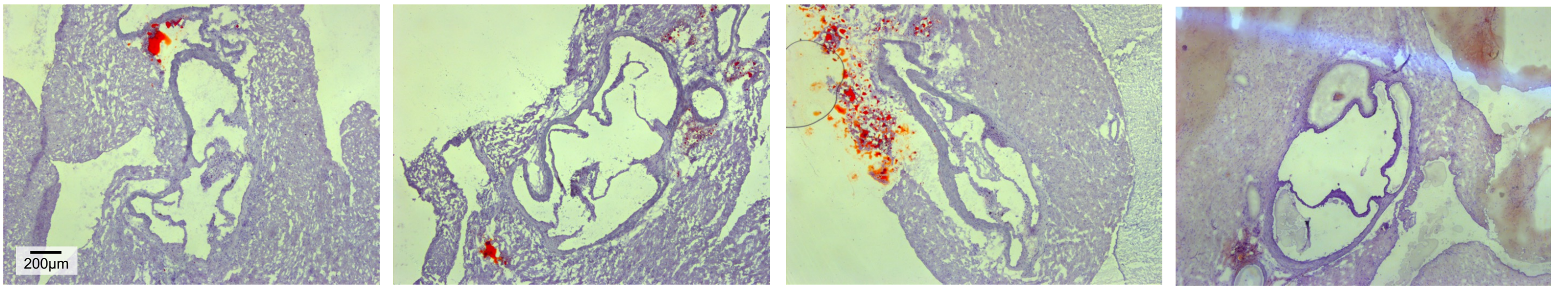

Adamts5$^{\Delta cat}$

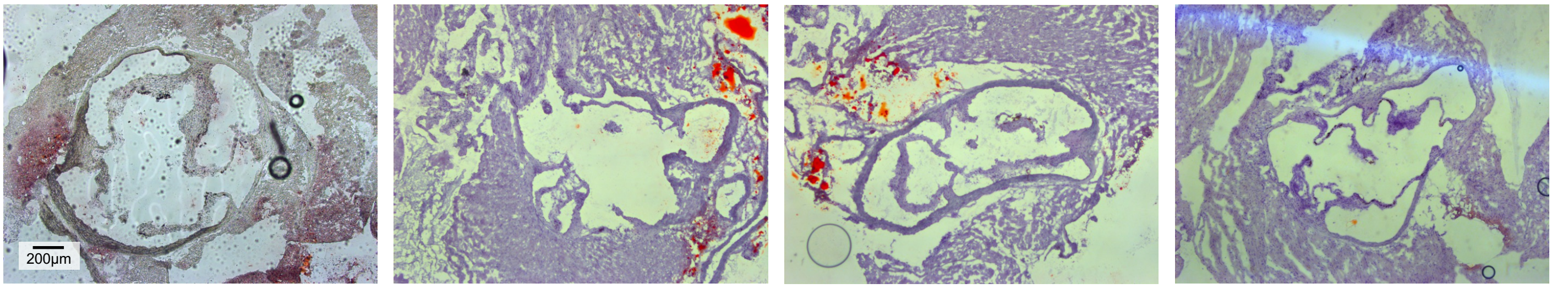

ApoE$^{-/-}$

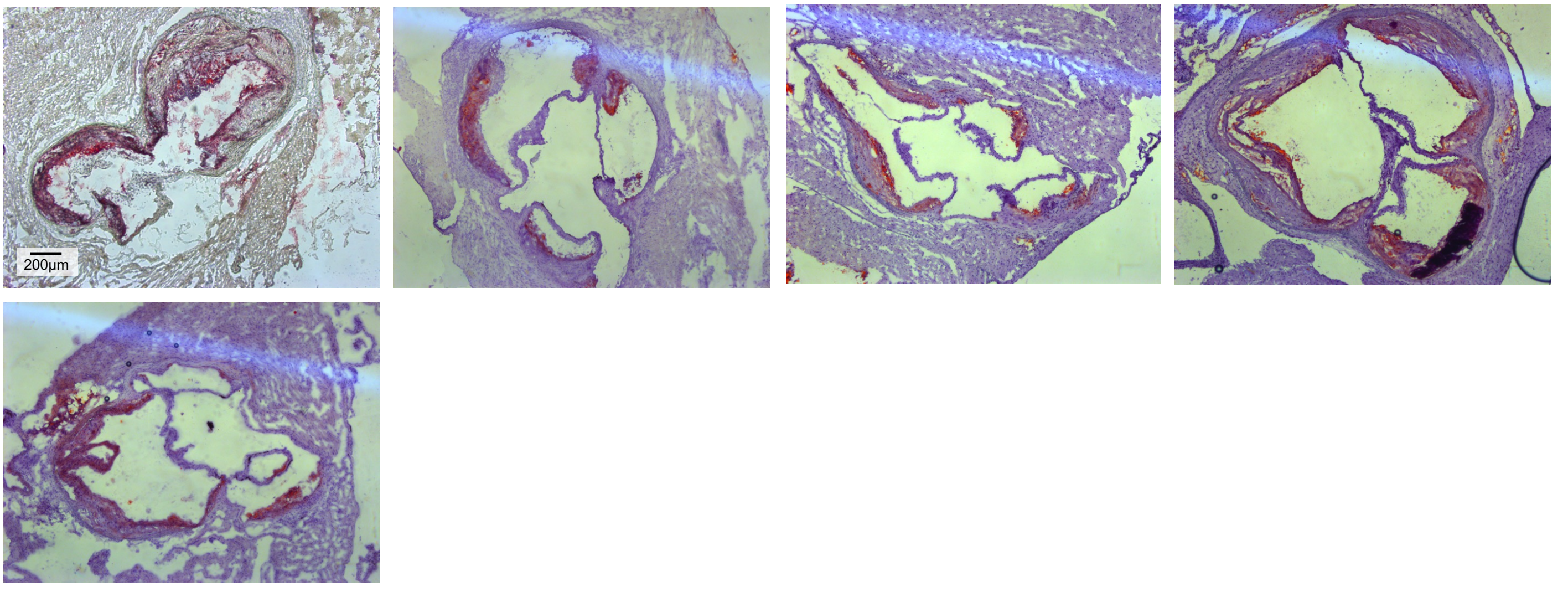

DKO

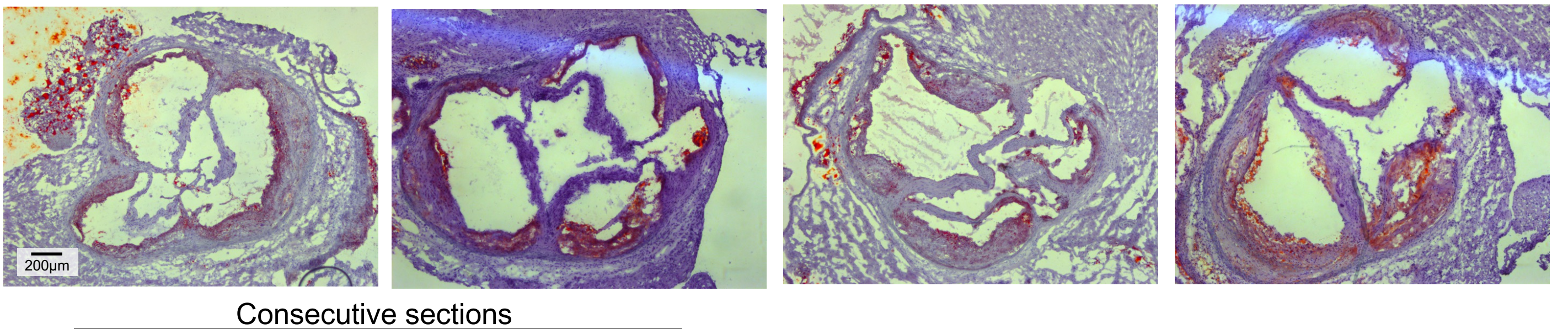

Consecutive sections

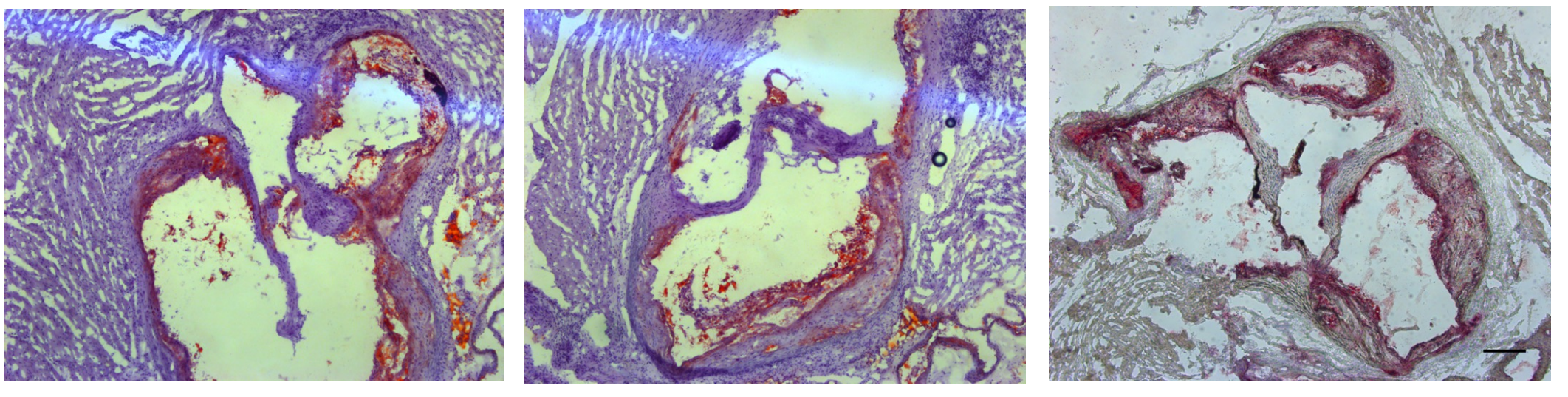

All Immunofluorescence staining images used in quantification in Figure S9B

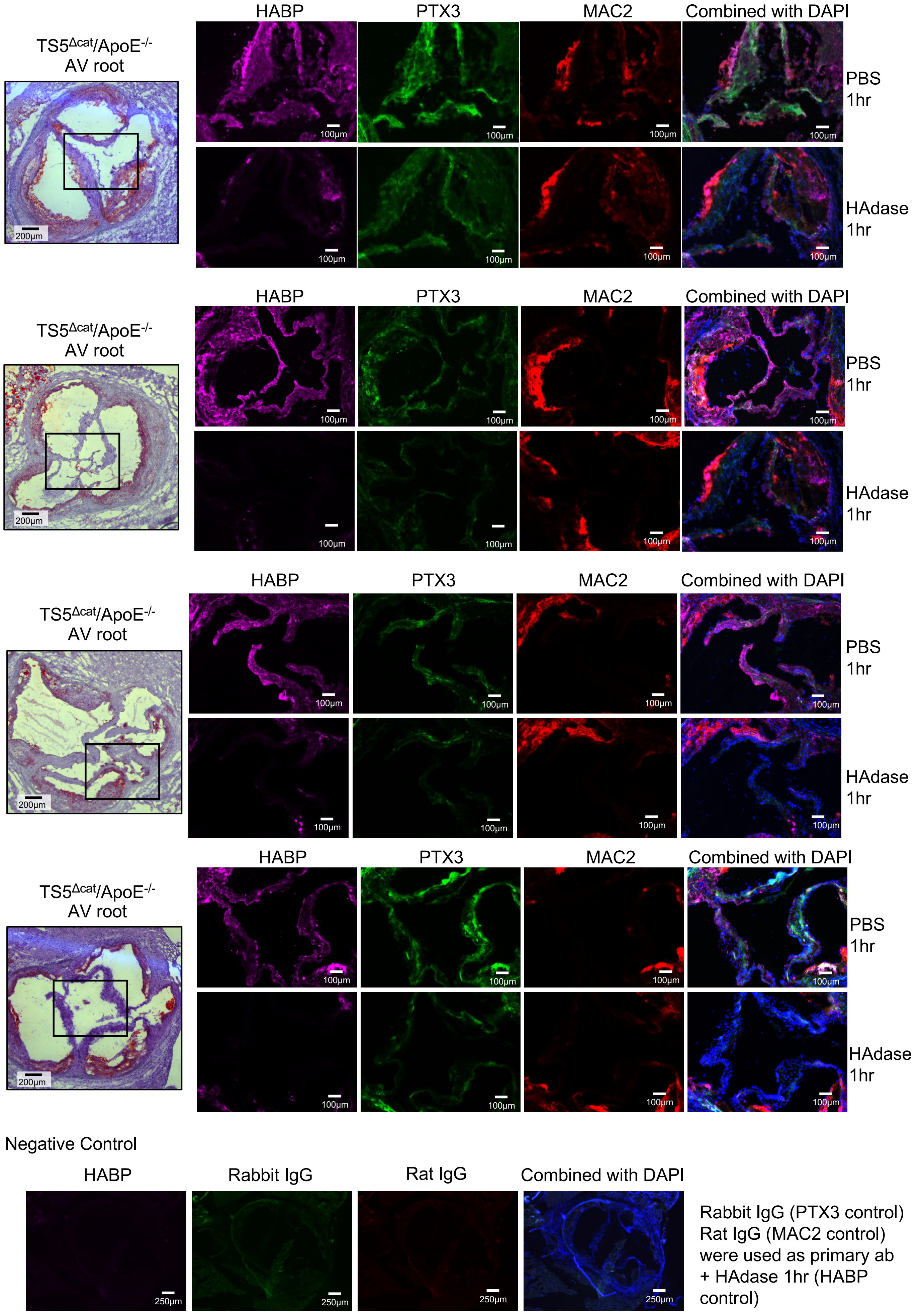

Supplement: Supplementary file 2 [file atv-46-e323867-s002.pdf]
