# Supplementary material for: Aortic Stenosis Hyalectan Remodeling Revealed by Proteomics and Glycoproteomics
Source: Arterioscler Thromb Vasc Biol. 2026 Jul 16;46(9):e323867. doi: 10.1161/ATVBAHA.126.323867 (PMC13506200; doi:10.1161/ATVBAHA.126.323867)

Full unedited Western blot for Figure 1B

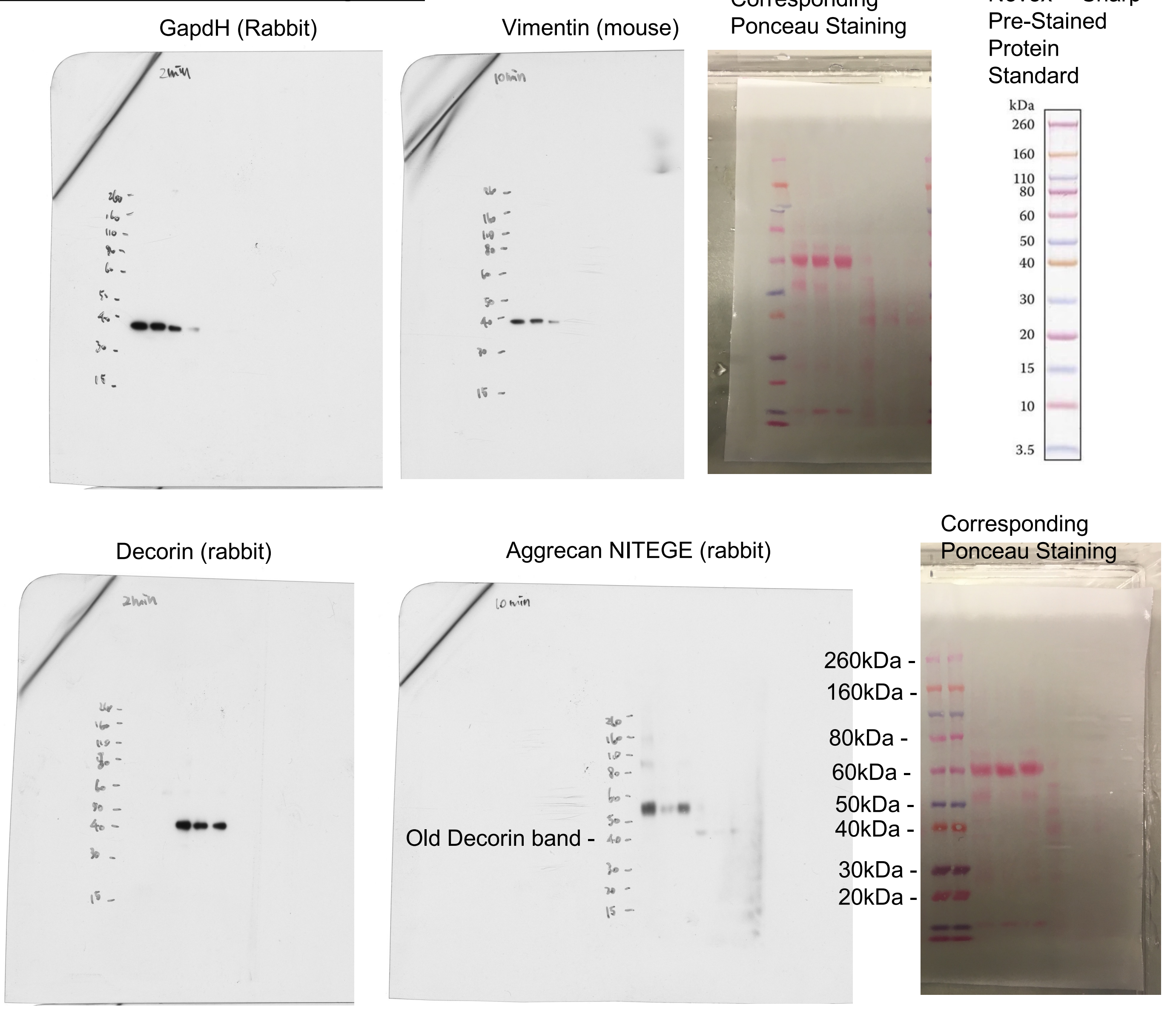

The post-ECL membrane was incubated in 0.1% sodium azide for 15 to 30 minutes to permanently inactivate the previous HRP, then wash thoroughly before directly applying new primary antibody usually from a different host species.

Full unedited Western blot for Figure 1B

For VCAN core and ACAN core, due to its high molecular weight, it was overrun

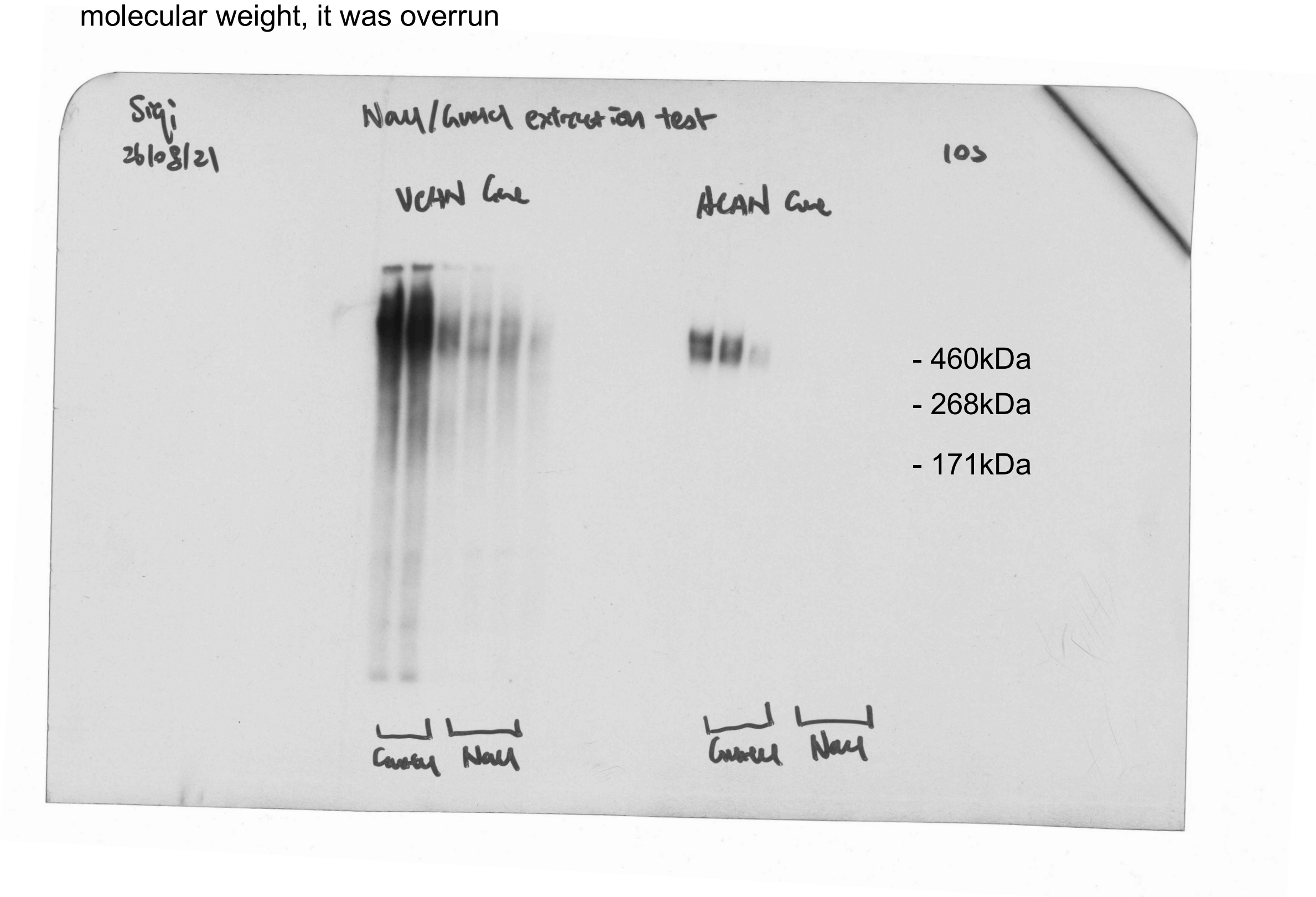

Corresponding Ponceau Staining

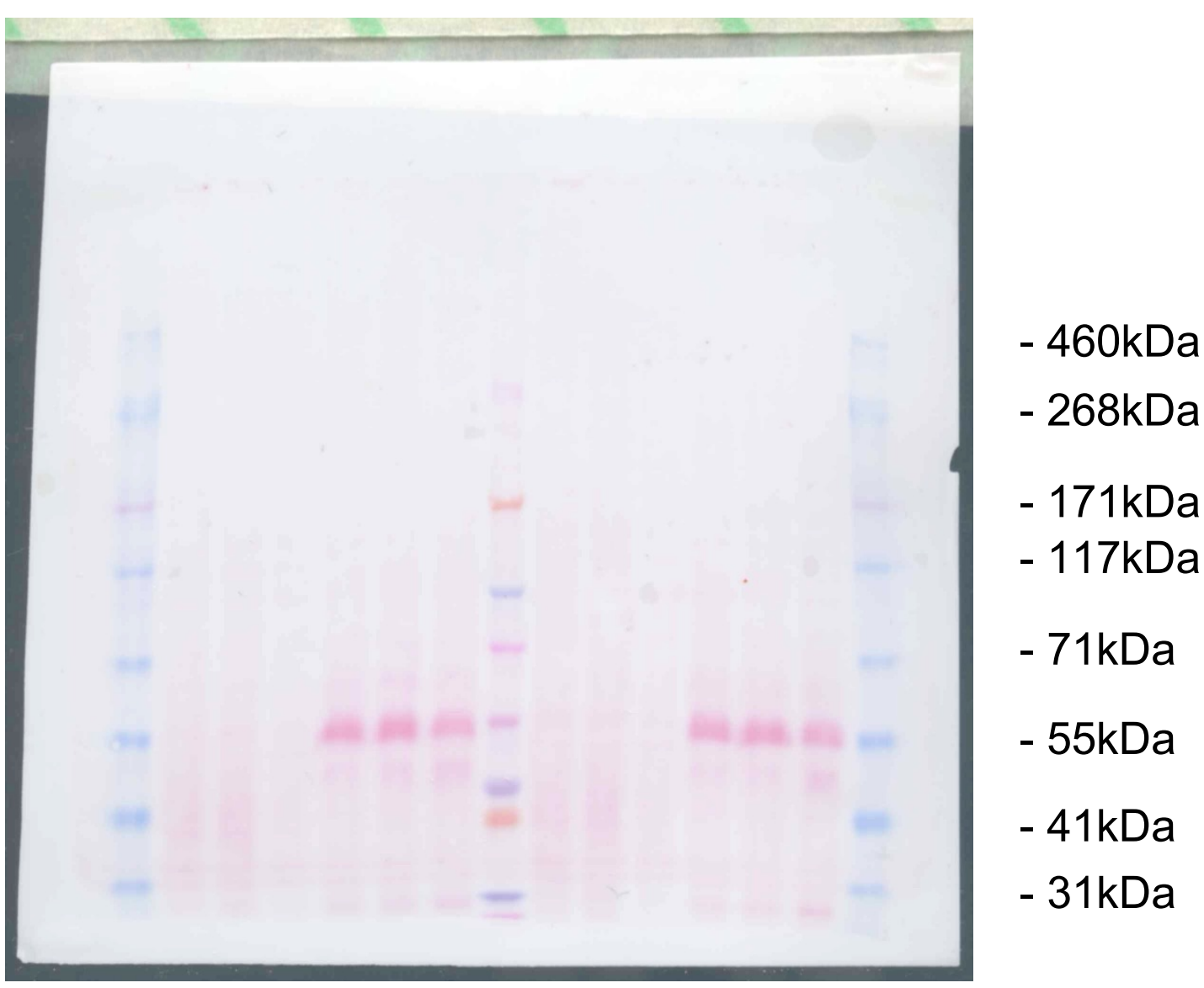

HiMark™ Pre-stained Protein Standard

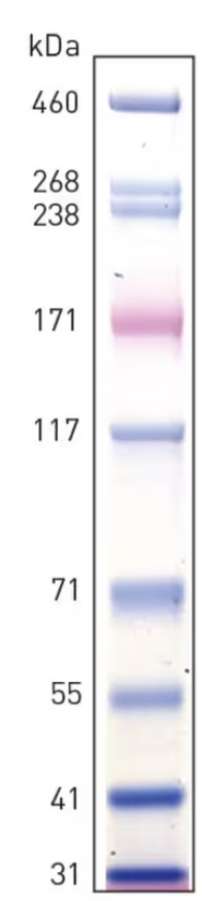

Full unedited Western blot for Figure 4B

Vimentin (mouse)

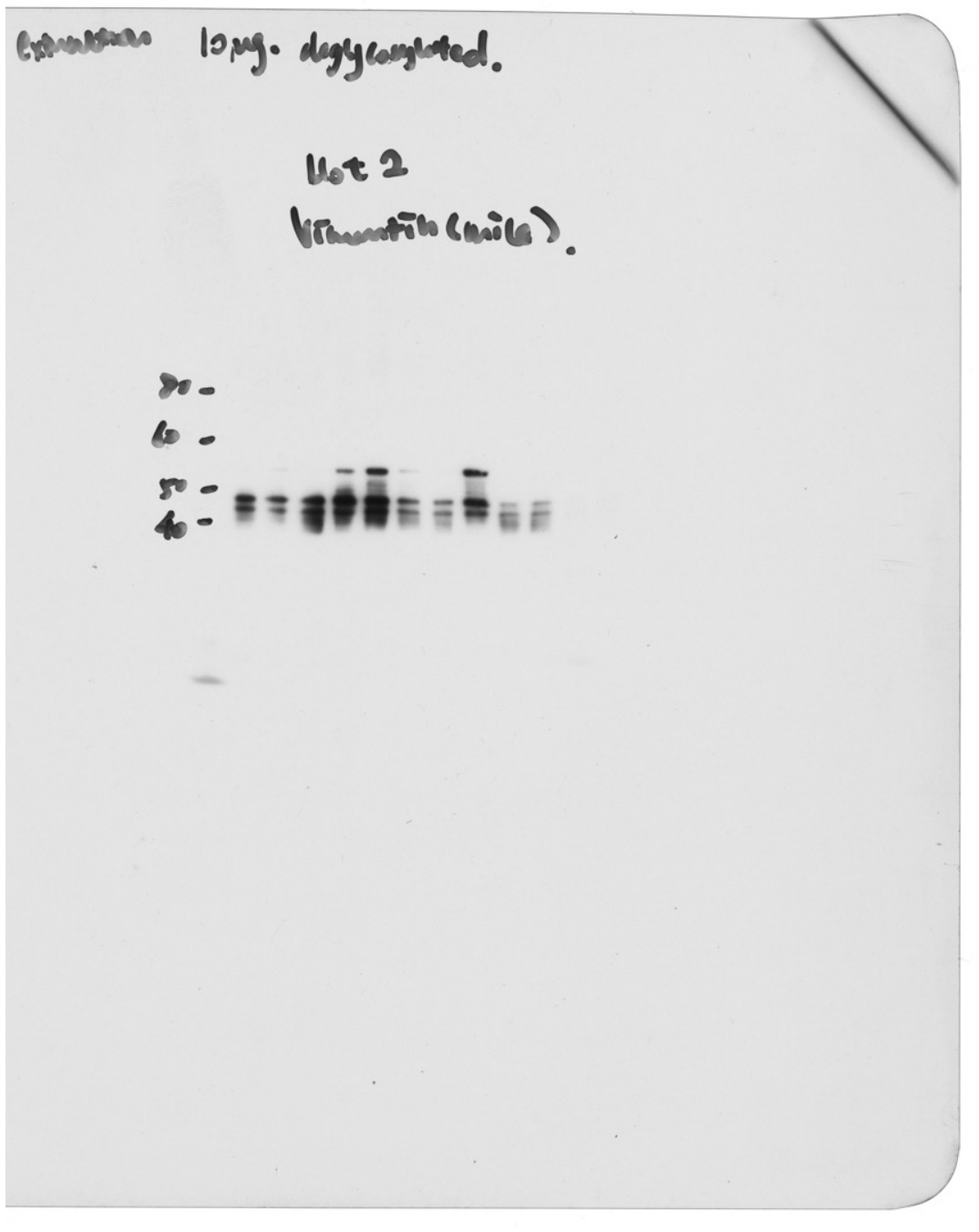

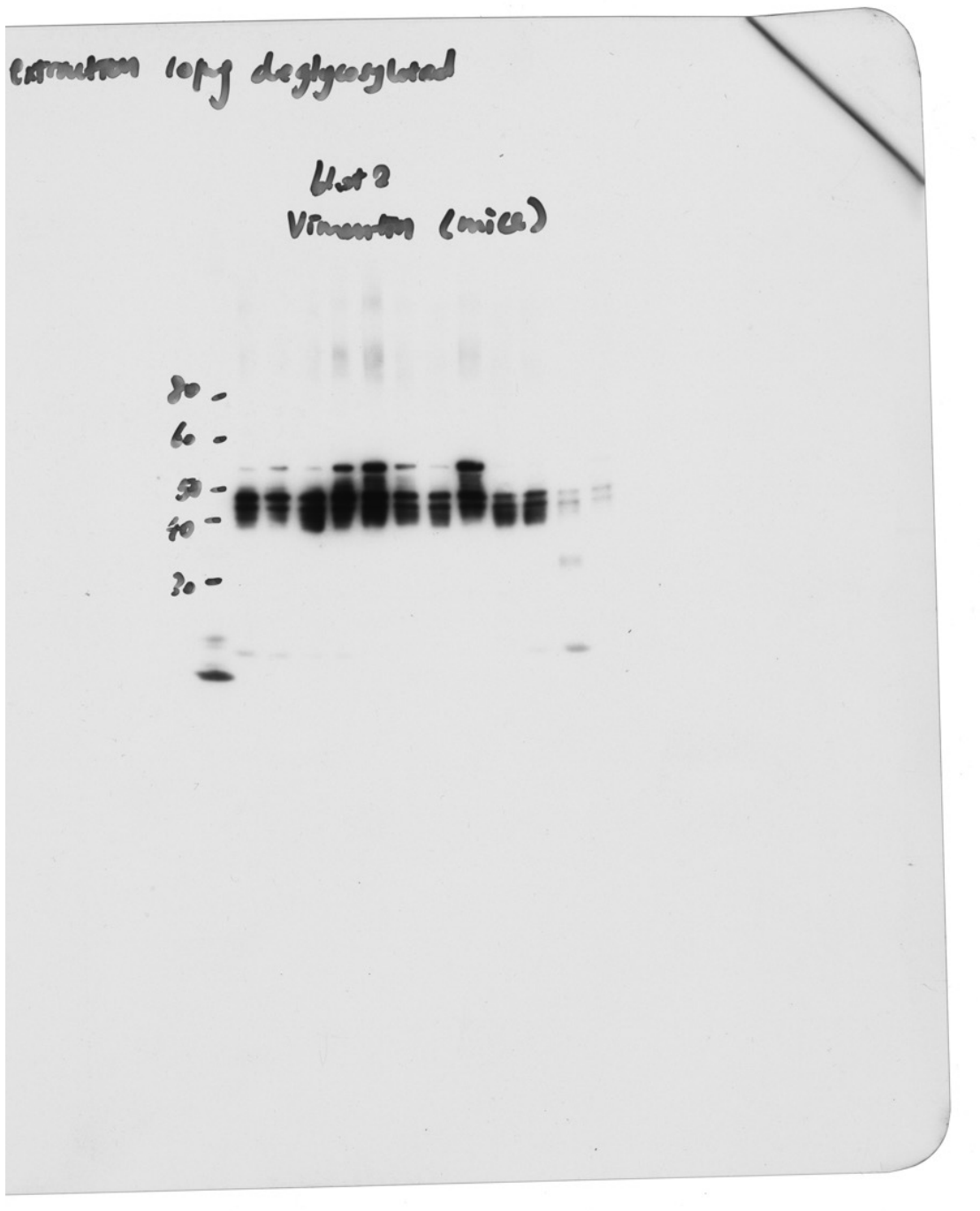

ACTA2 (rabbit)

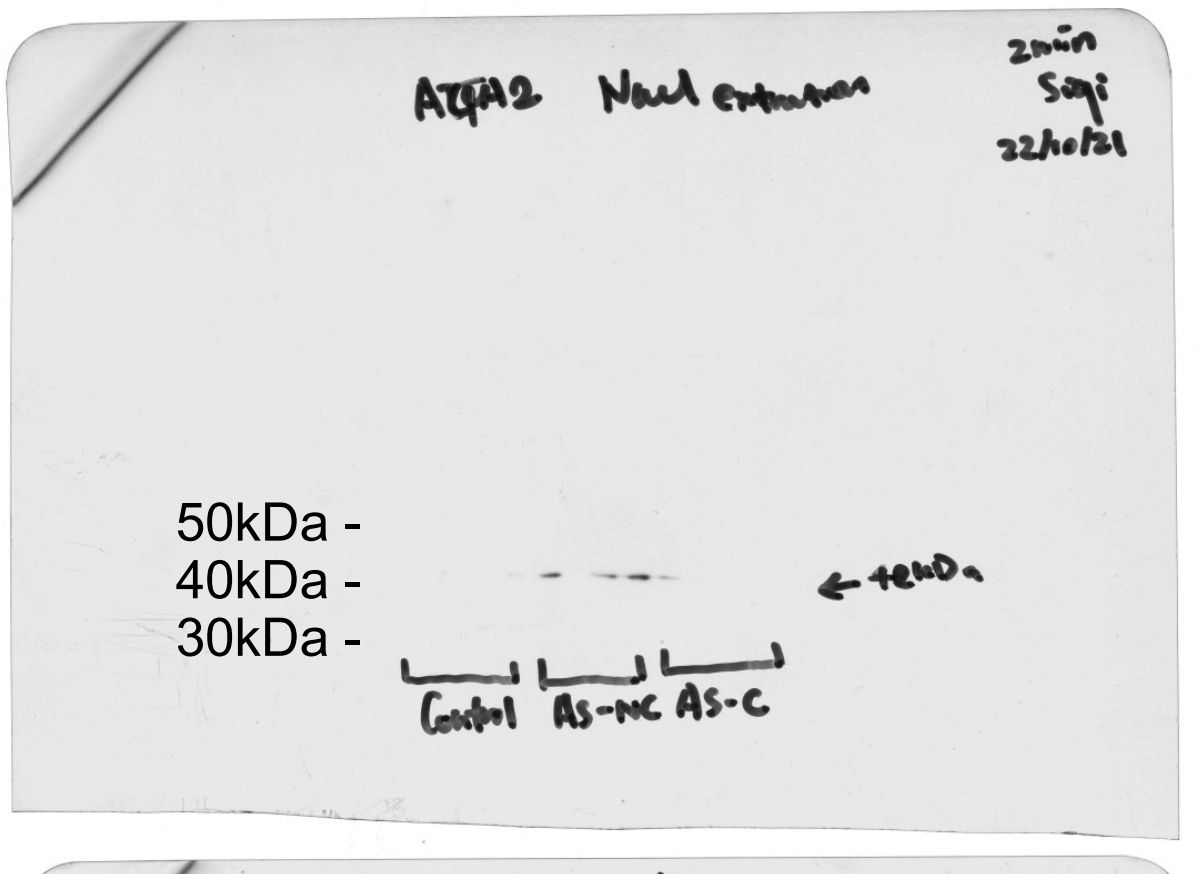

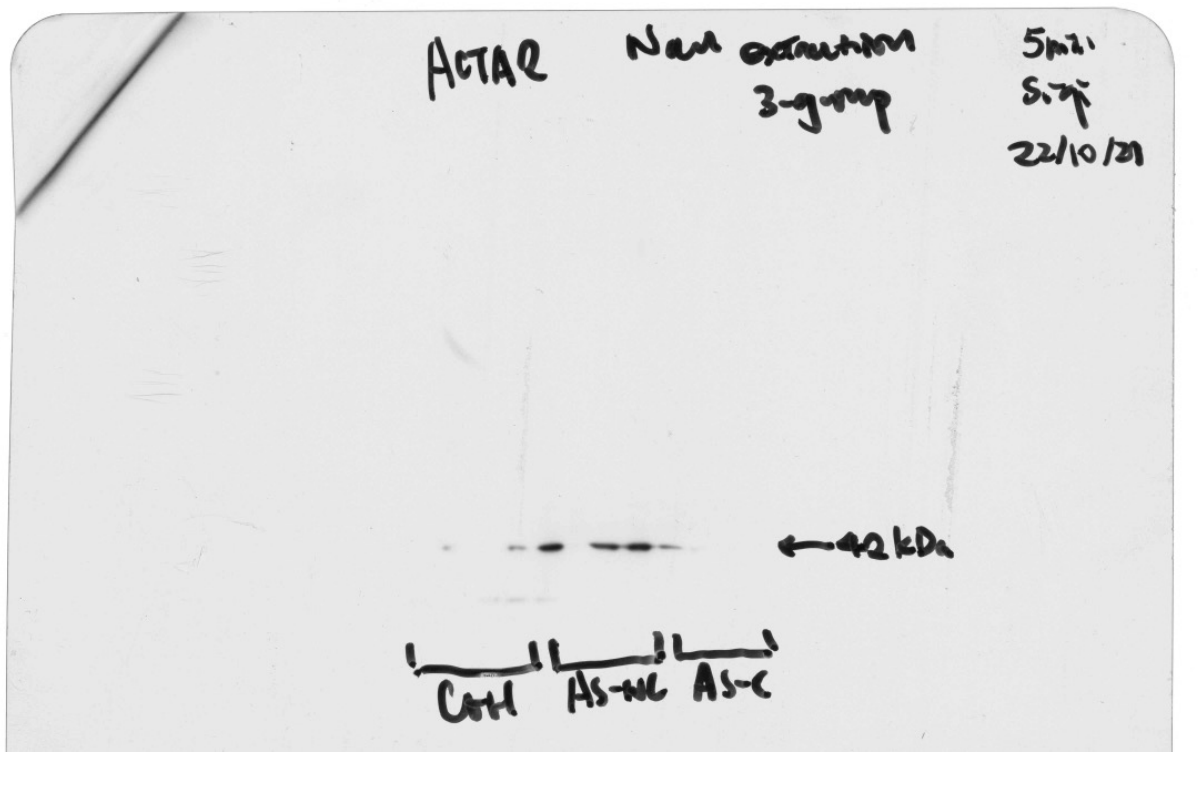

Novex™ Sharp Pre-Stained Protein Standard

Corresponding Ponceau Staining

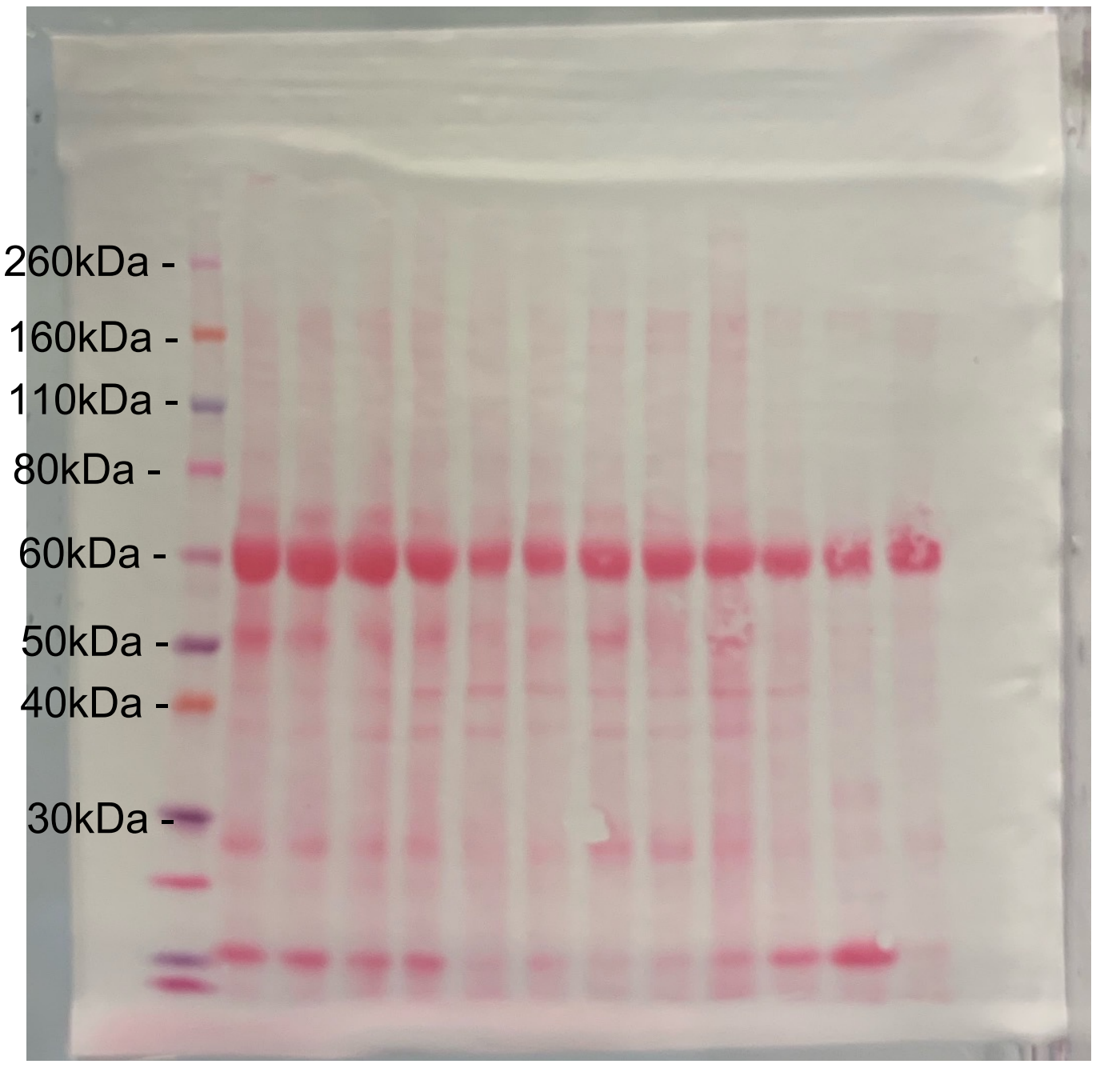

The post-ECL membrane was incubated in 0.1% sodium azide for 15 to 30 minutes to permanently inactivate the previous HRP, then wash thoroughly before directly applying new primary antibody from a different host species.

Full unedited Western blot for Figure 4B

COMP (rat)

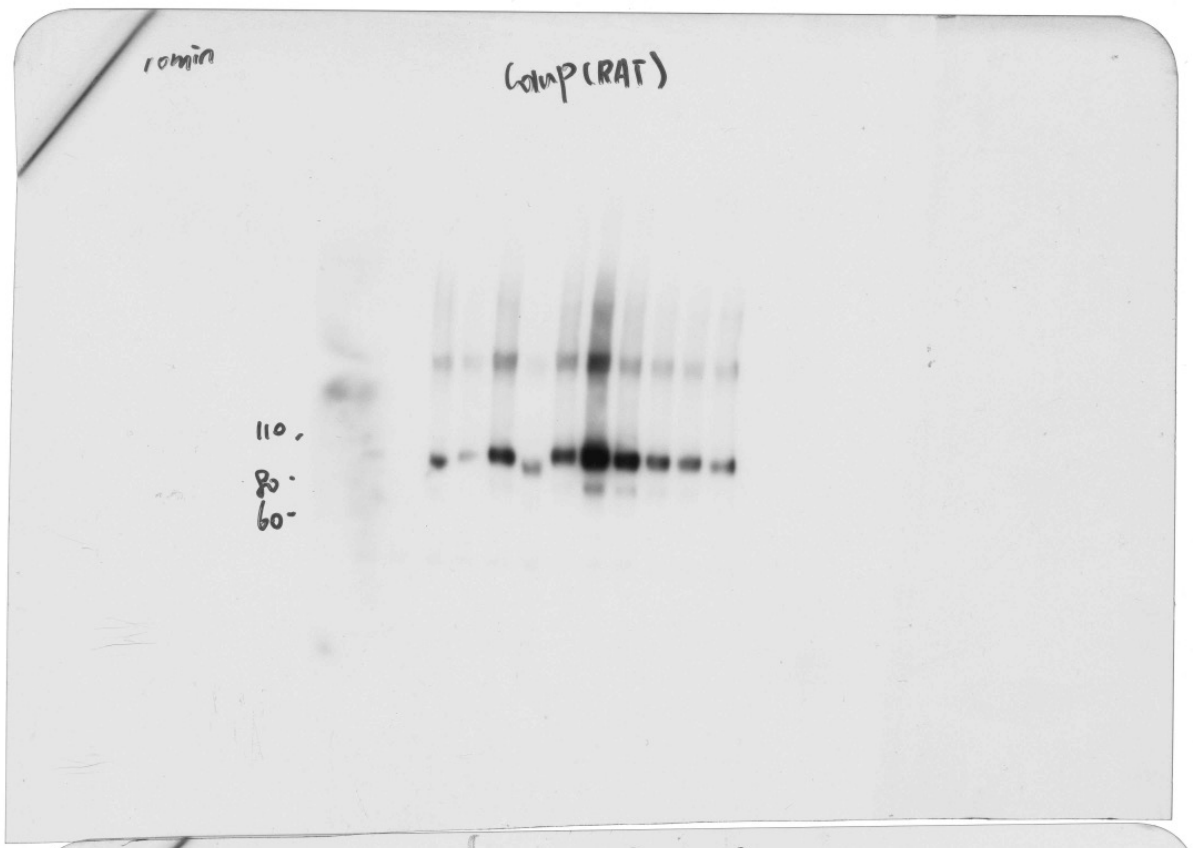

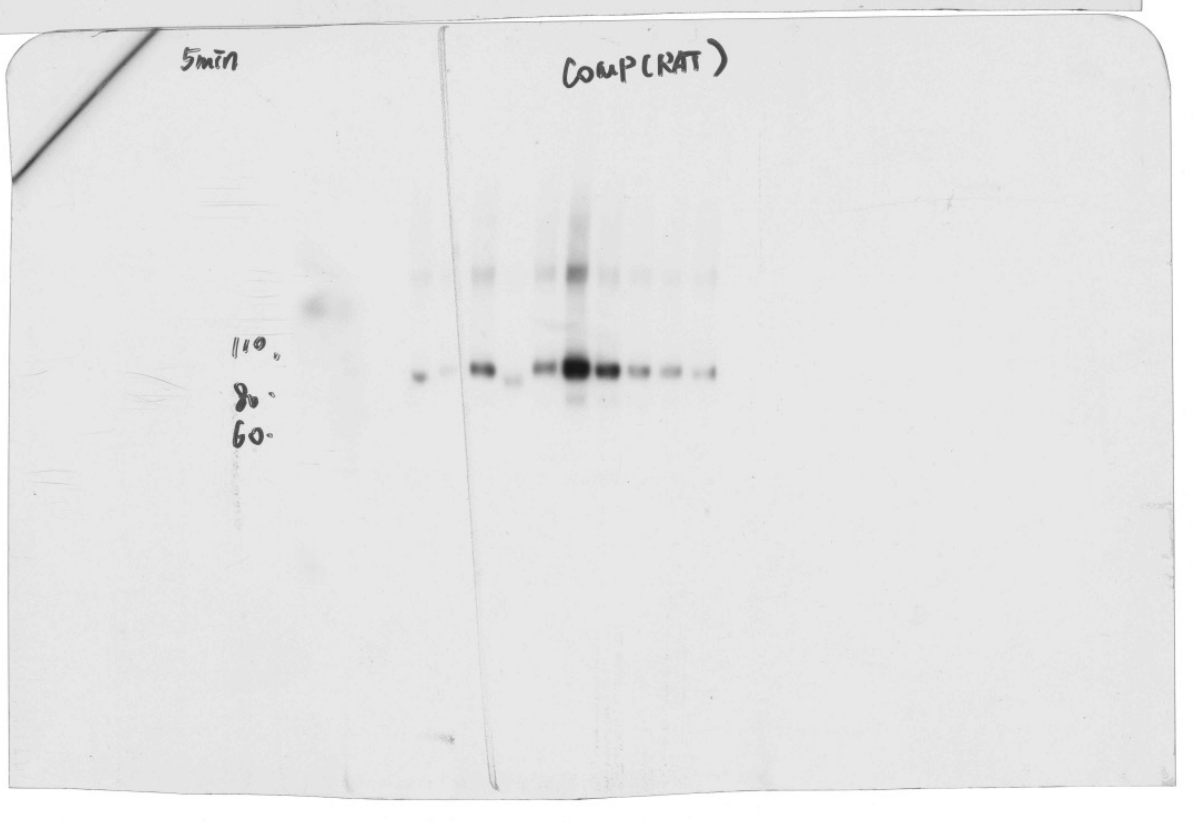

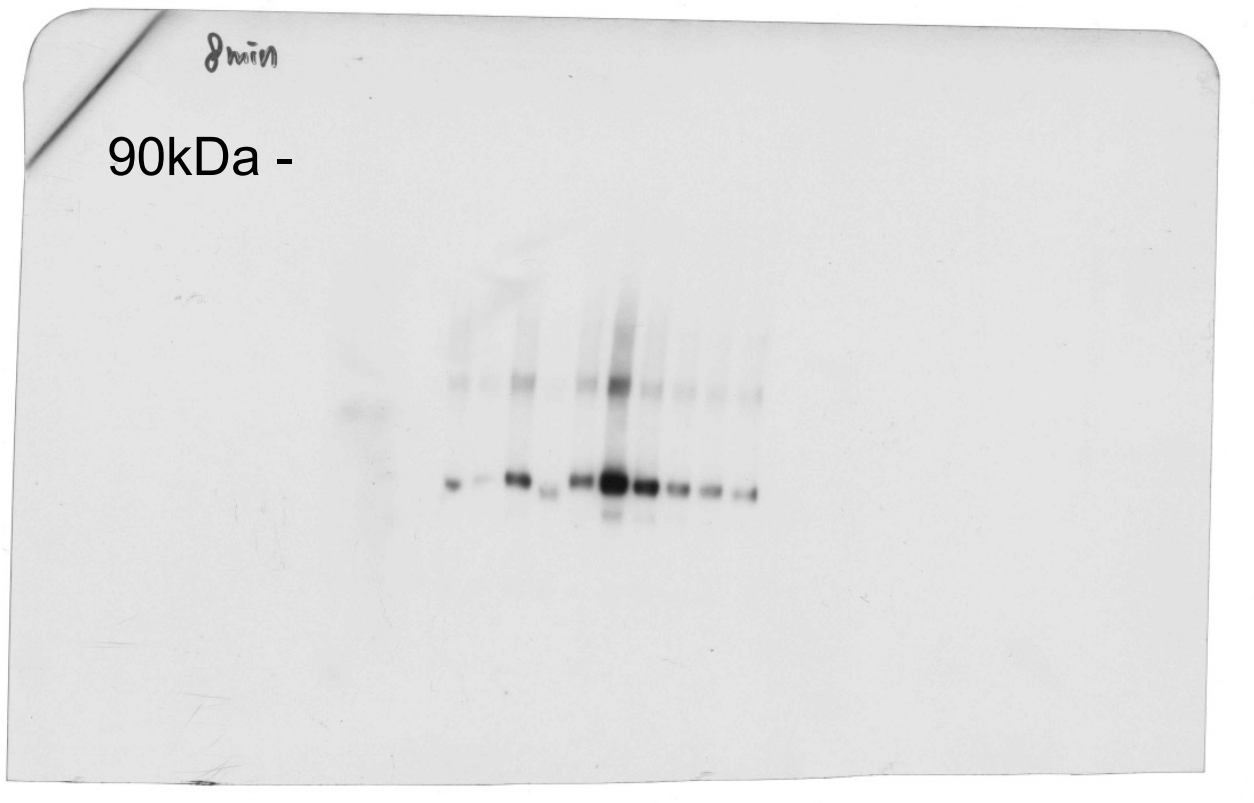

The post-ECL membrane was incubated in 0.1% sodium azide for 15 to 30 minutes to permanently inactivate the previous HRP, then wash thoroughly before directly applying new primary antibody from a different host species.

Novex™ Sharp Pre-Stained Protein Standard

Corresponding Ponceau Staining

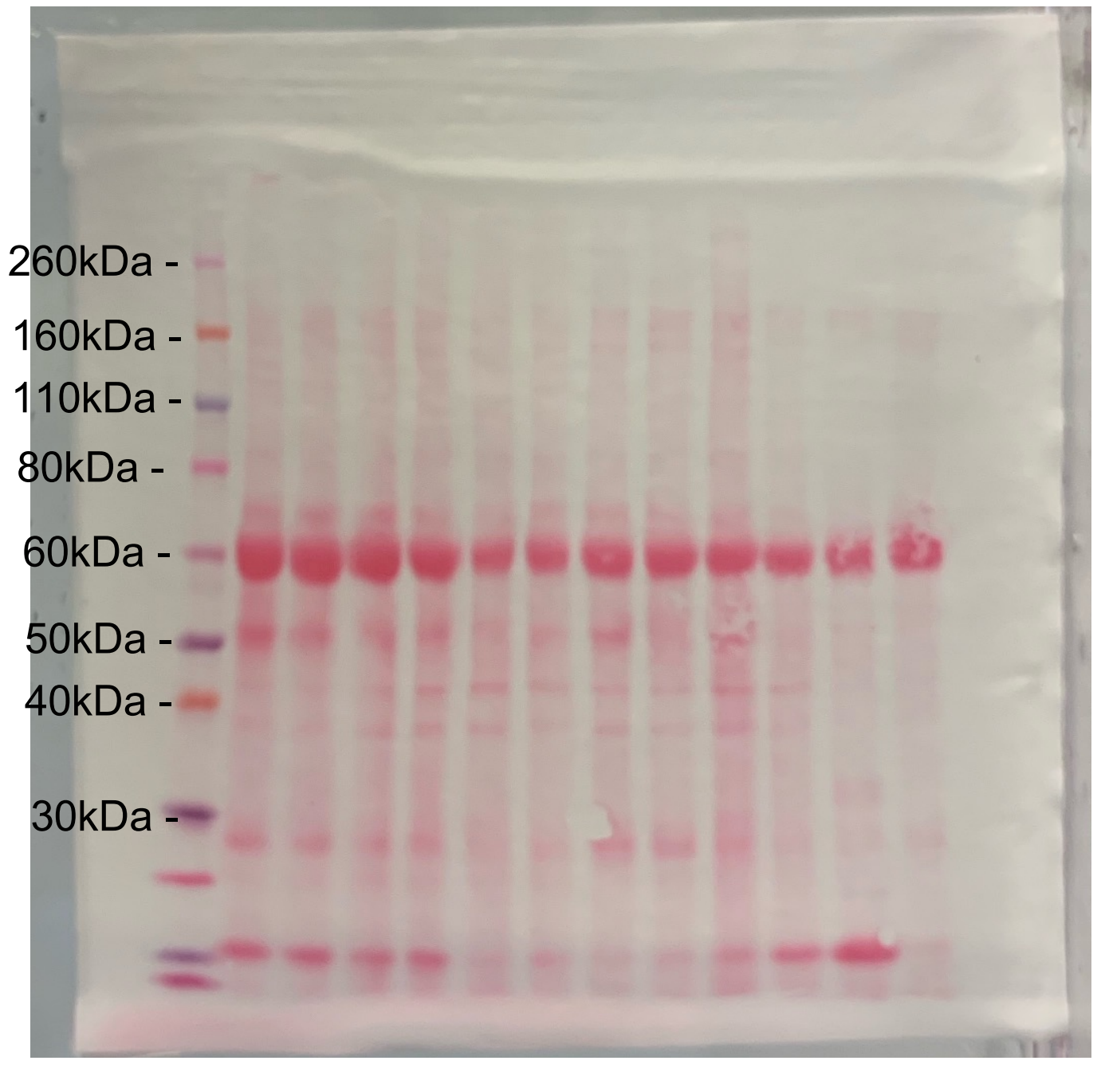

Full unedited Western blot for Figure 4B

ITIH1 (rabbit, channel 1)

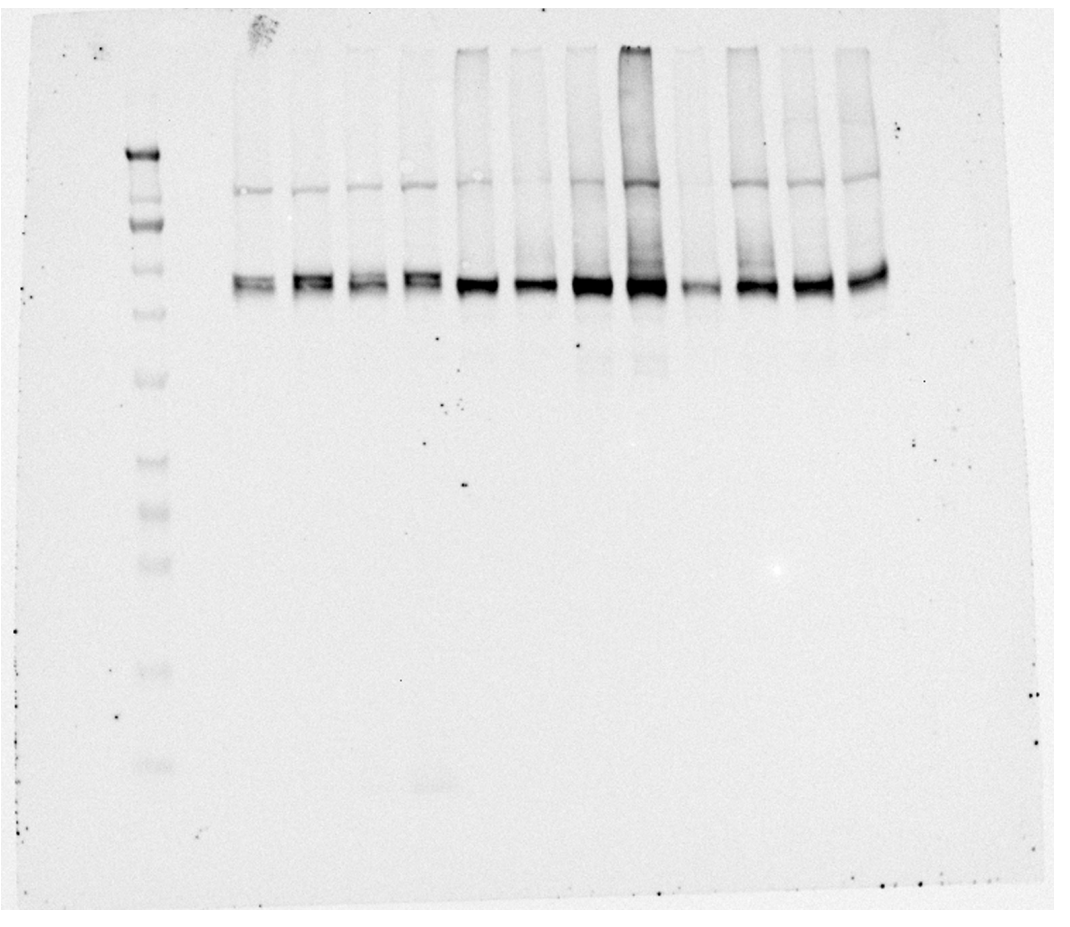

PTX3 (rat, channel 2)

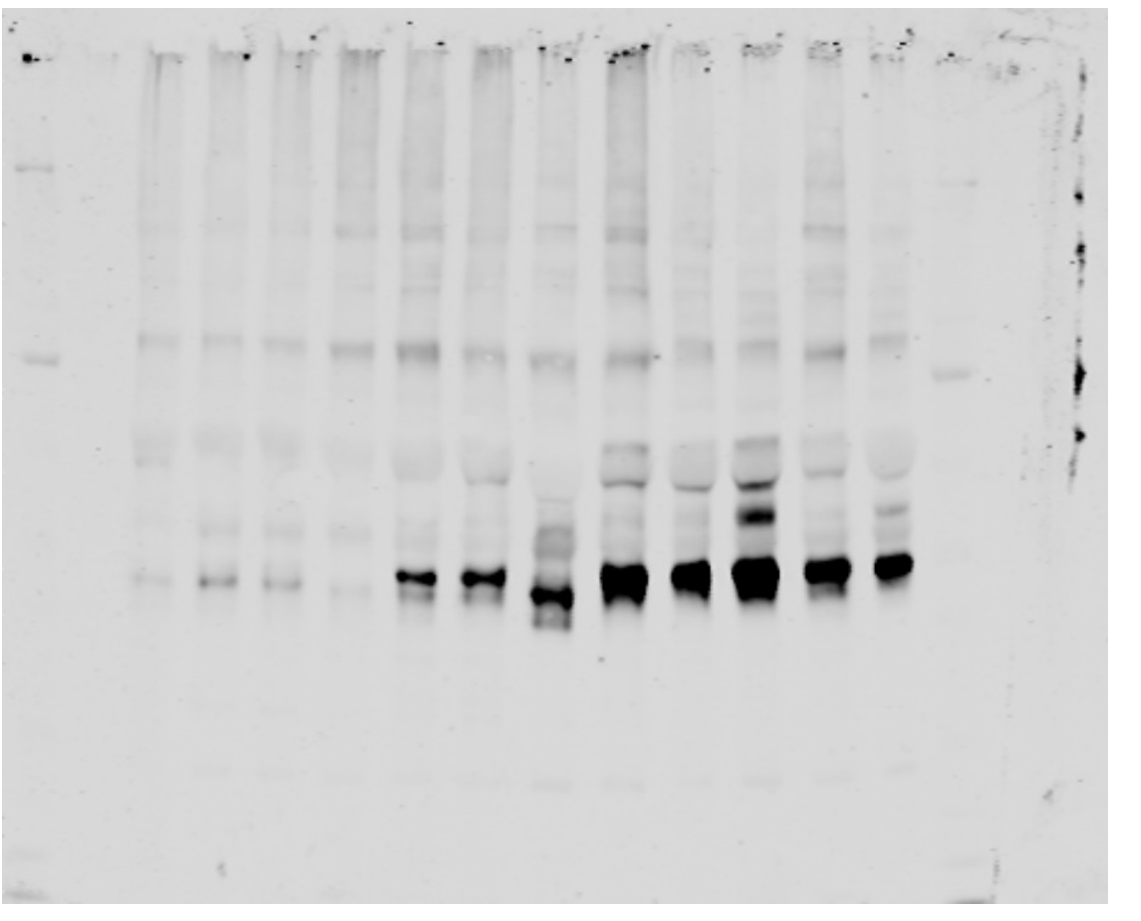

Corresponding Ponceau Staining

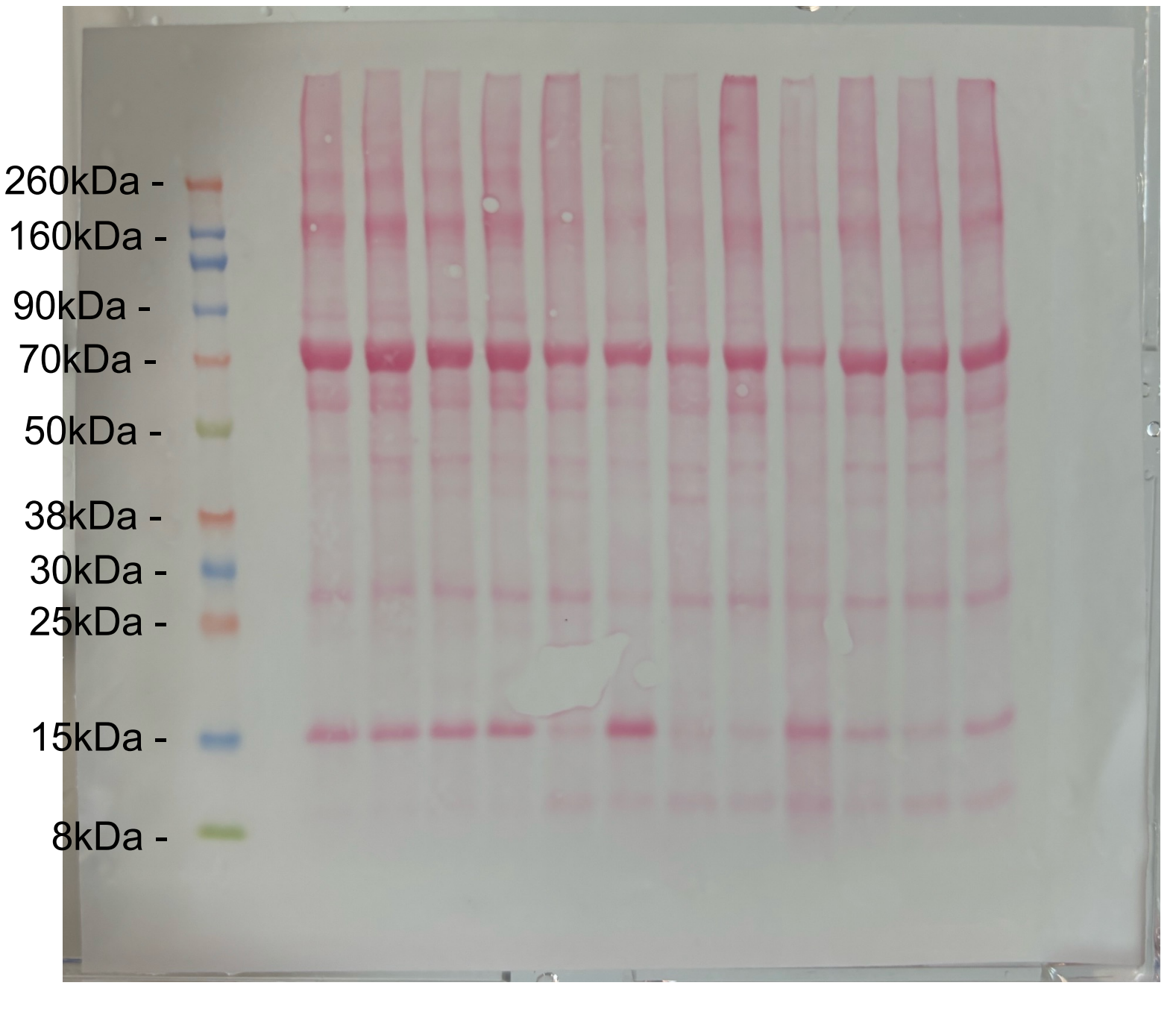

Chameleon Duo Pre-stained Protein Ladder

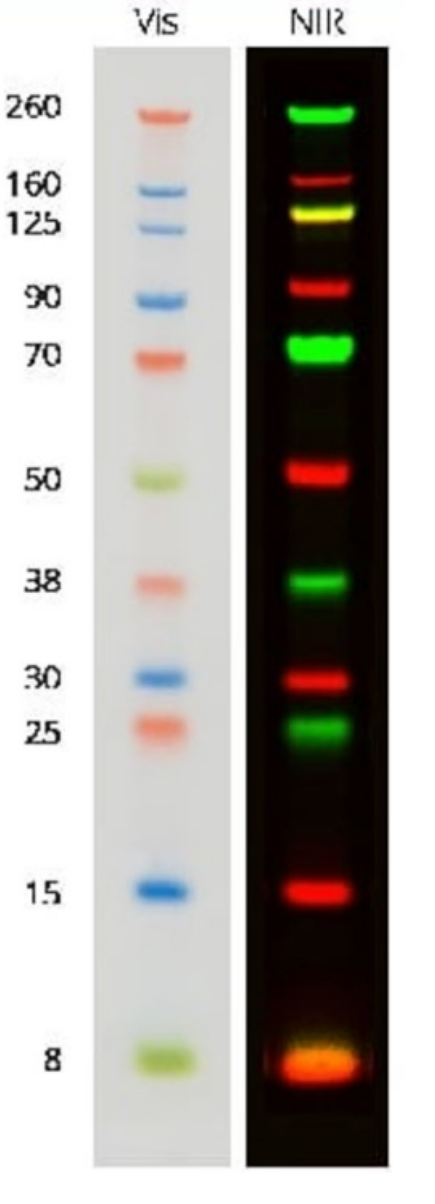

Full Western blot for Figure 5A and 6B

First set of 6 samples contribute to Figure 6B and second set of 6 samples contribute to Figure 5A

HAPLN1 (mouse)

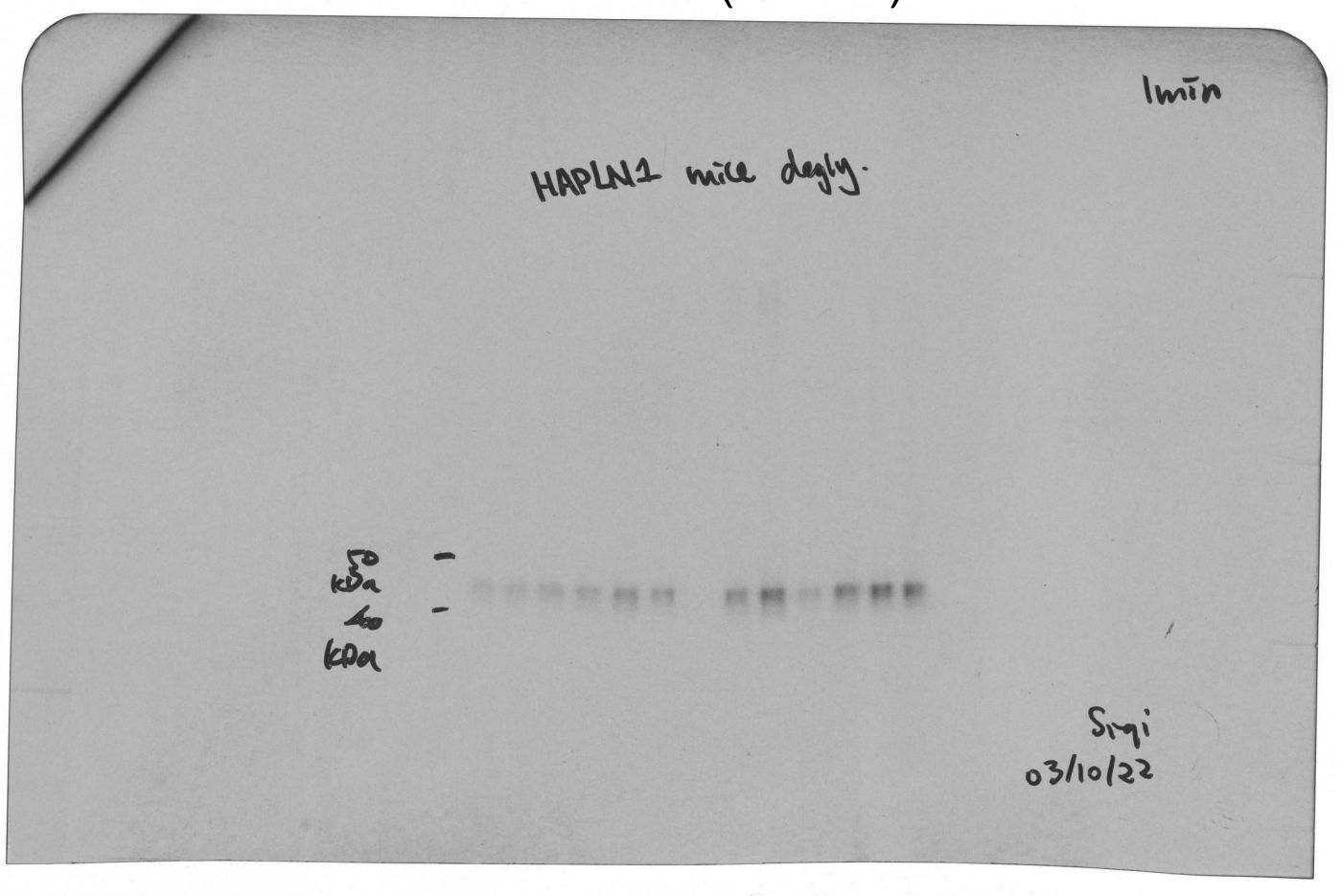

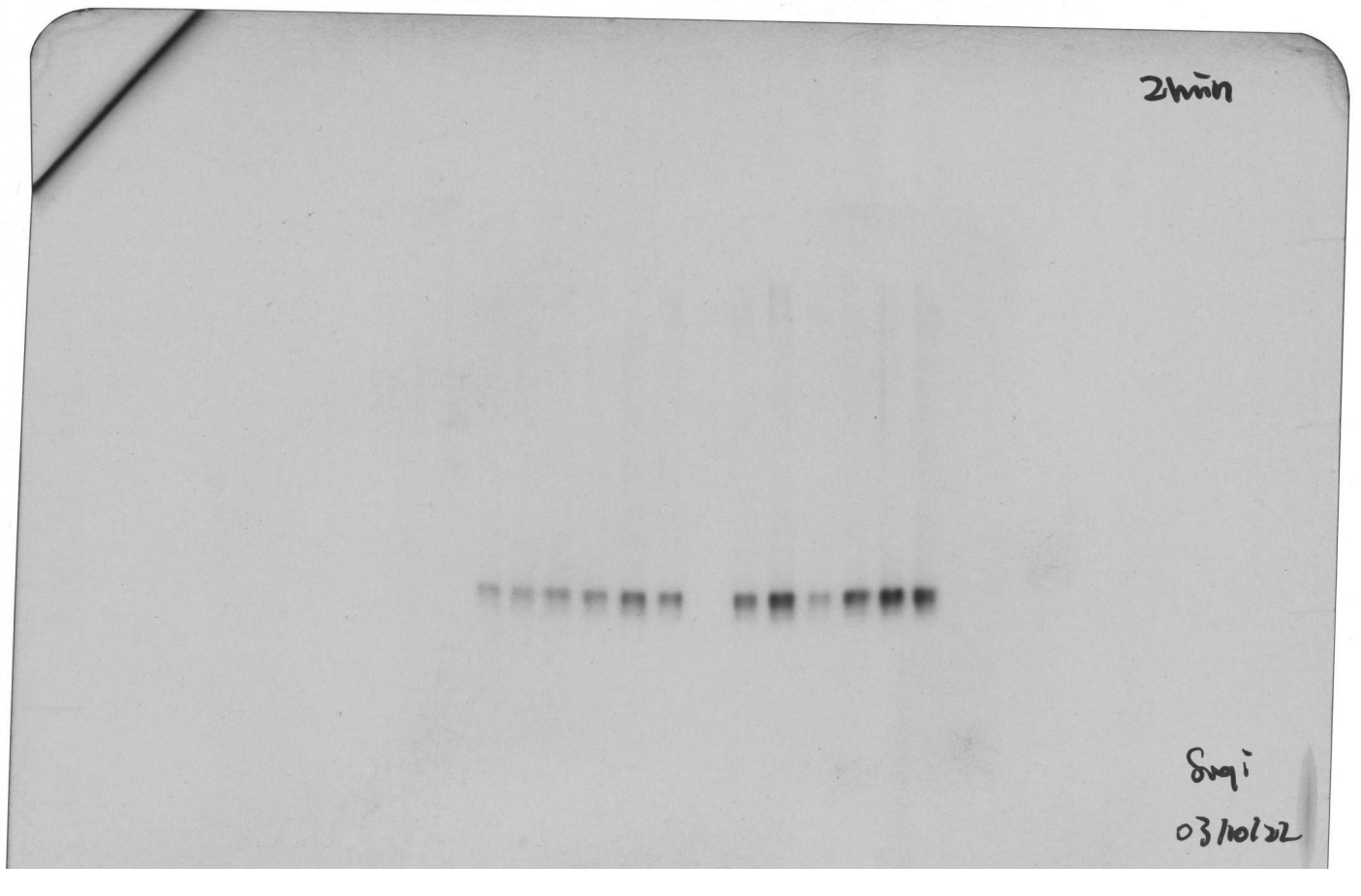

Versican
DPEAAE (rabbit)

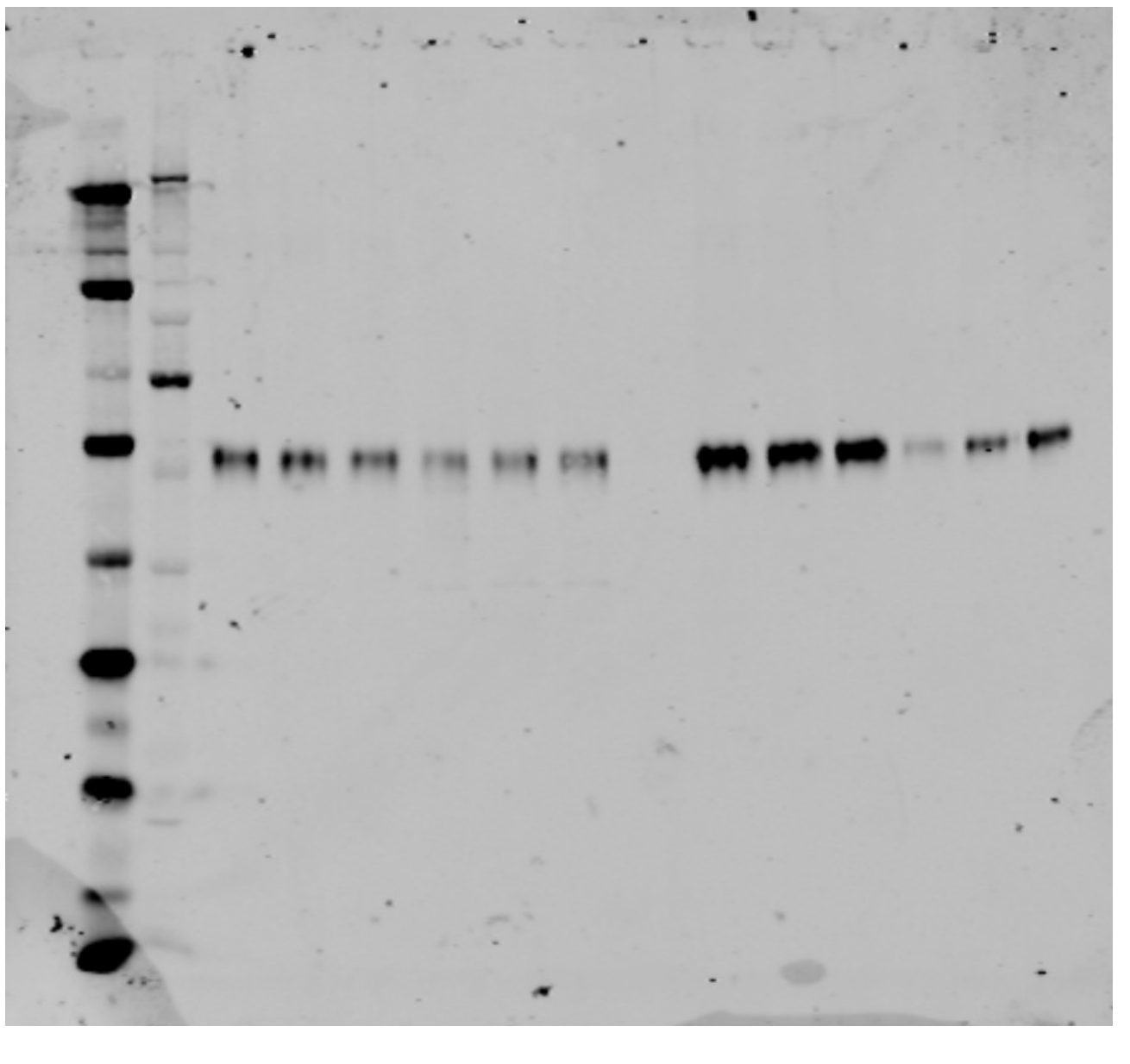

First lane marker Chameleon Duo Pre-stained Protein Ladder
Second lane marker is Bio-Rad Precision Plus Protein (unstained).

Aggrecan
NITEGE (rabbit)

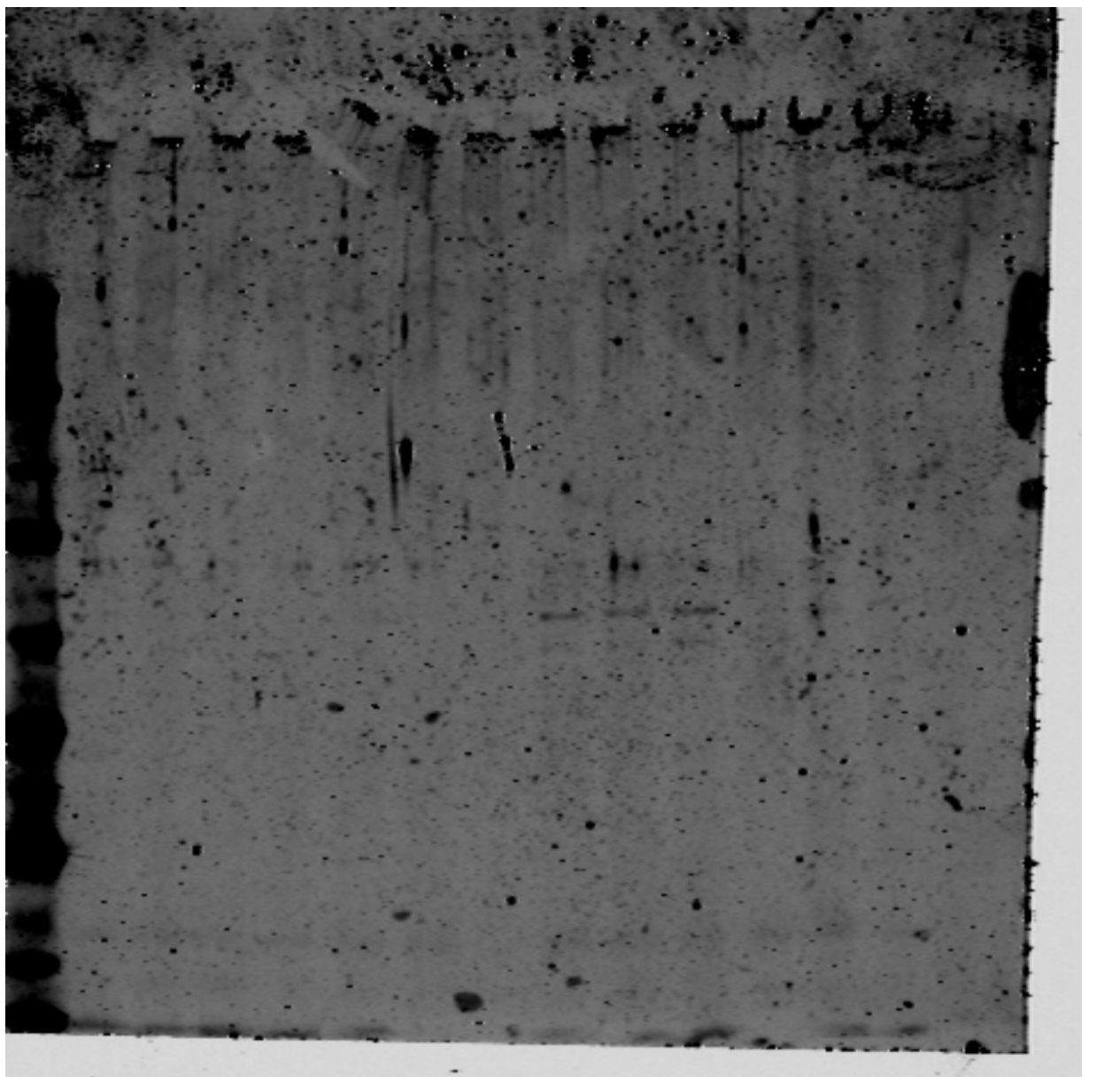

PTX3 (rabbit)

Previous VCAN band -

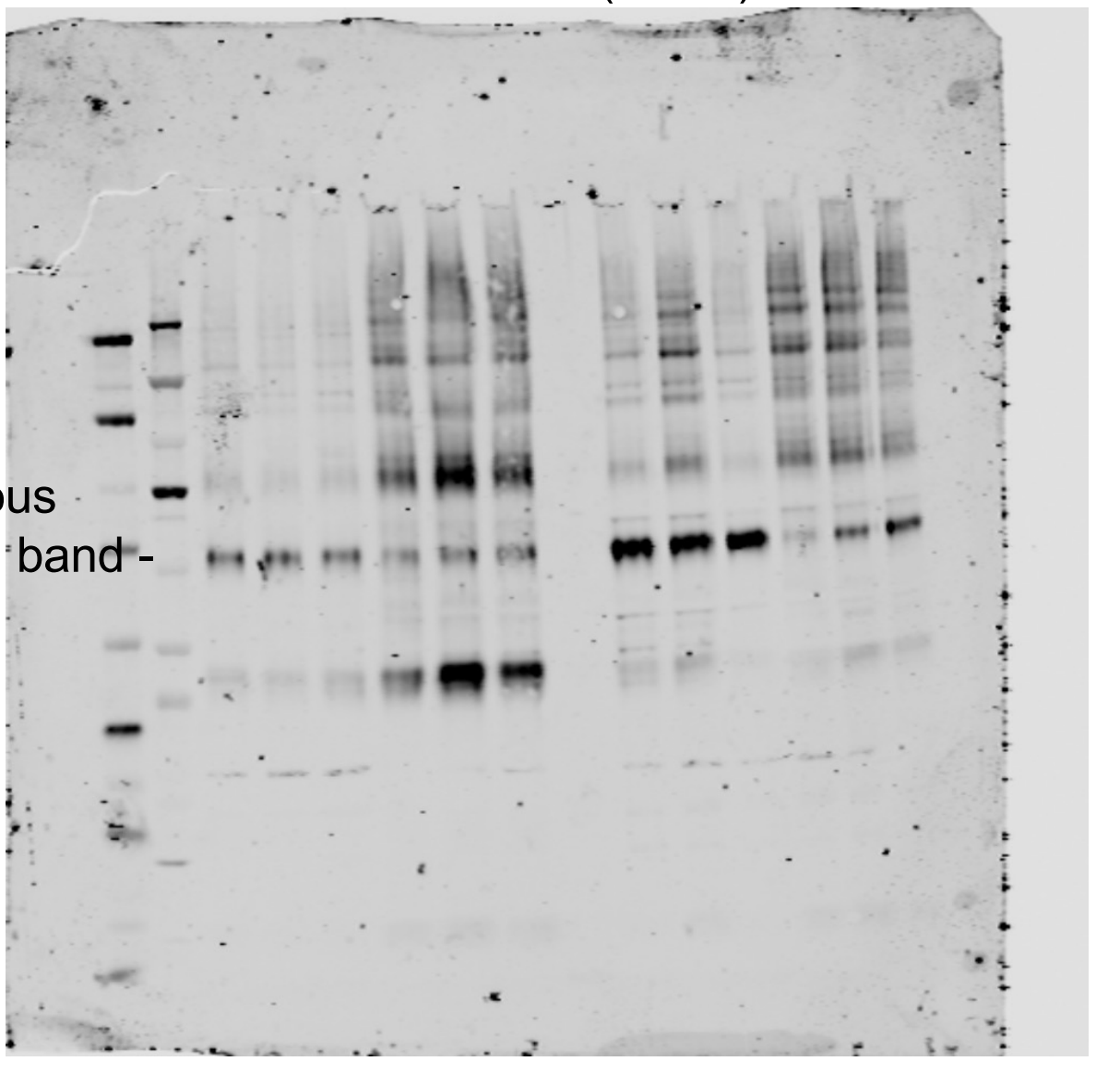

Corresponding
Ponceau Staining

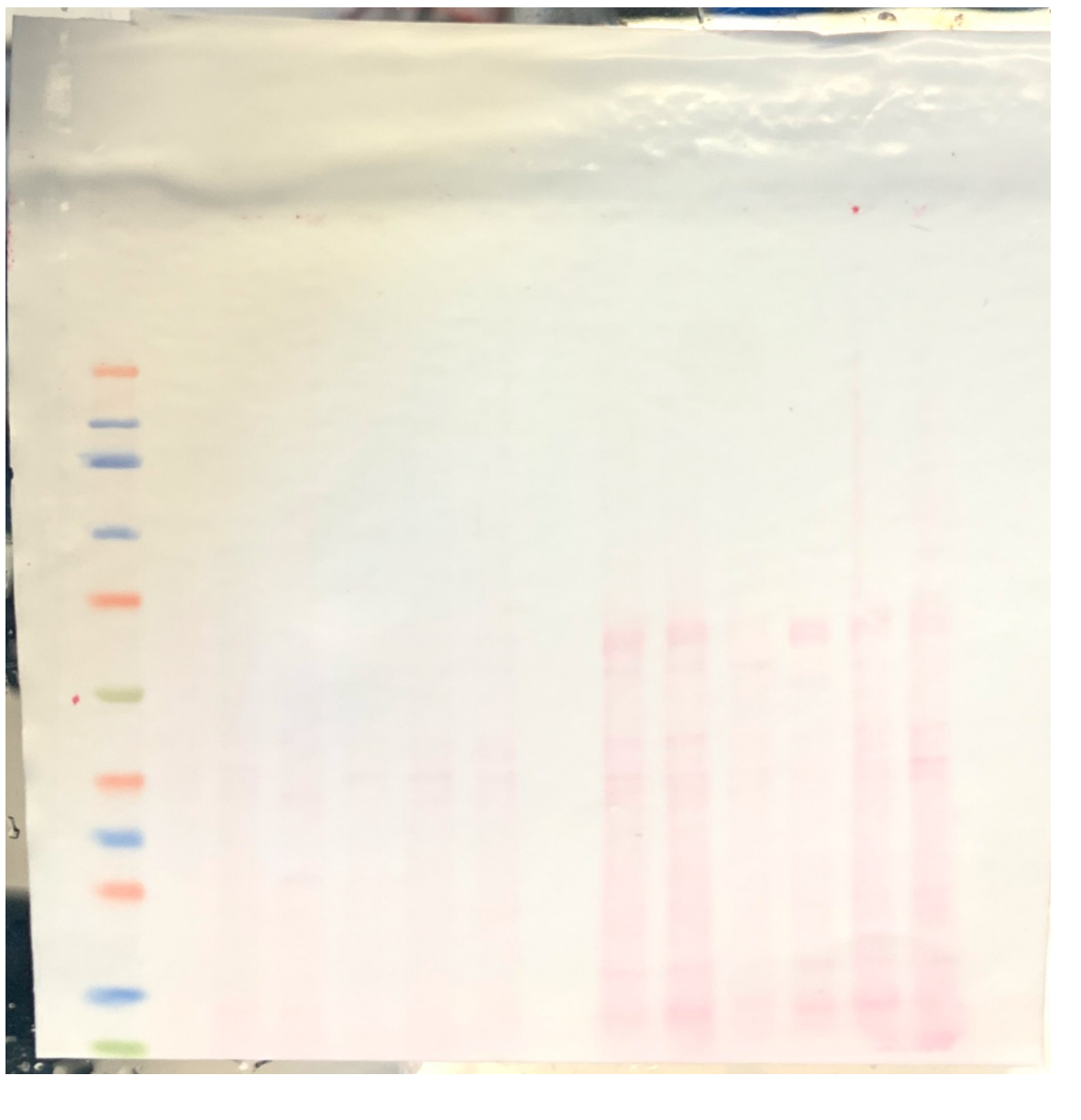

Full unedited Western blot for Figure 6F

Pull down extraction - reduced

ITIH1 (rabbit, channel 1)

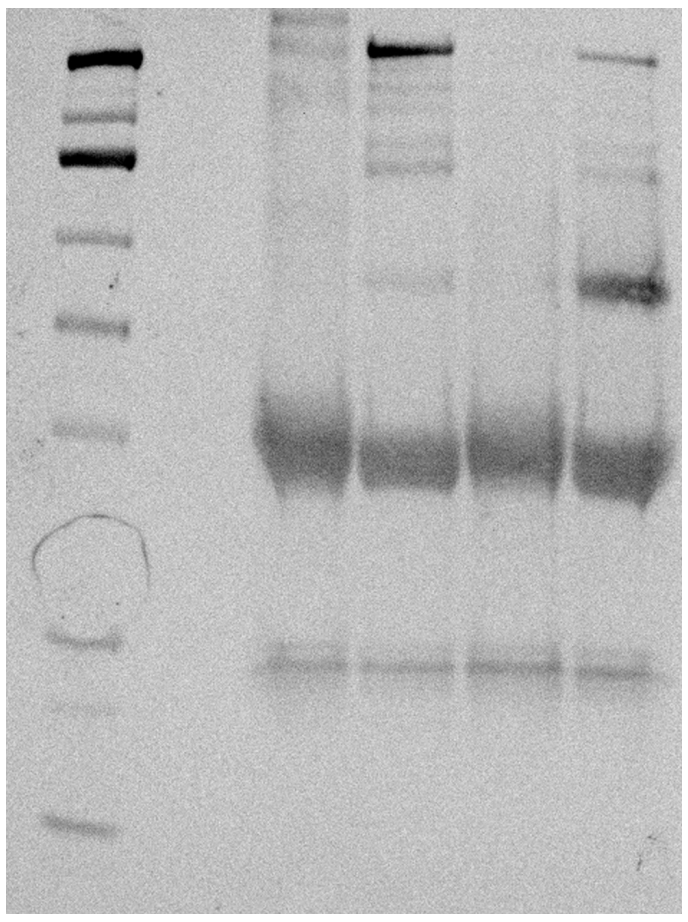

PTX3 (rat, channel 2)

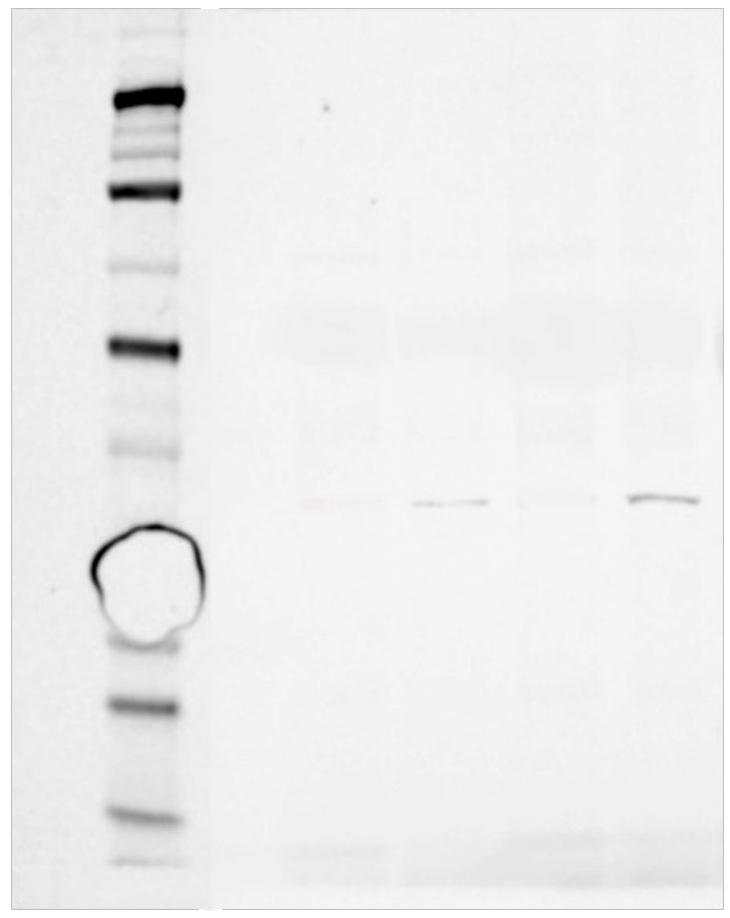

HABP (streptavidin, channel 3)

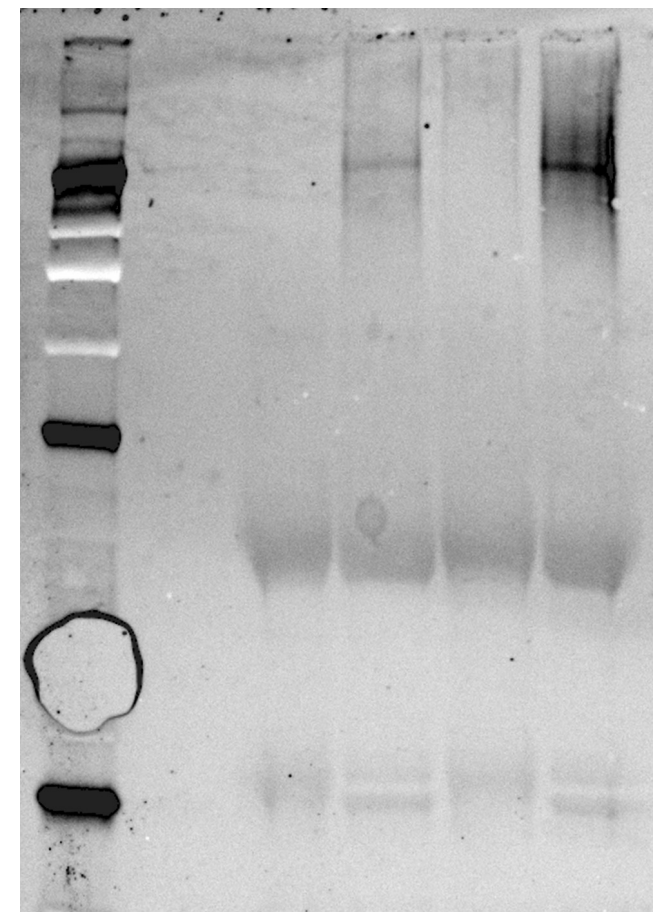

Supernatant - reduced

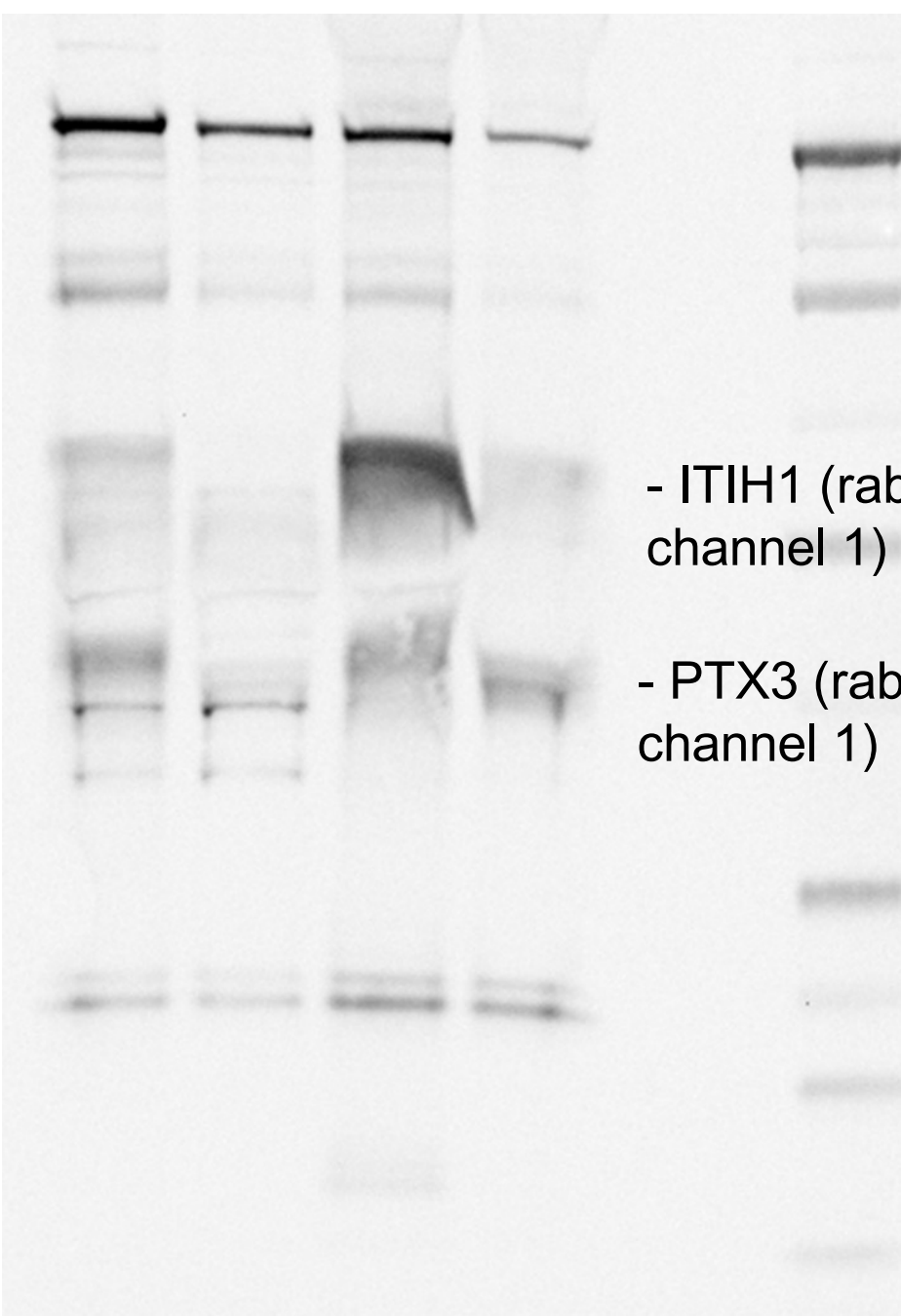

- ITIH1 (rabbit, channel 1)
- PTX3 (rabbit, channel 1)

HABP (streptavidin, channel 2)

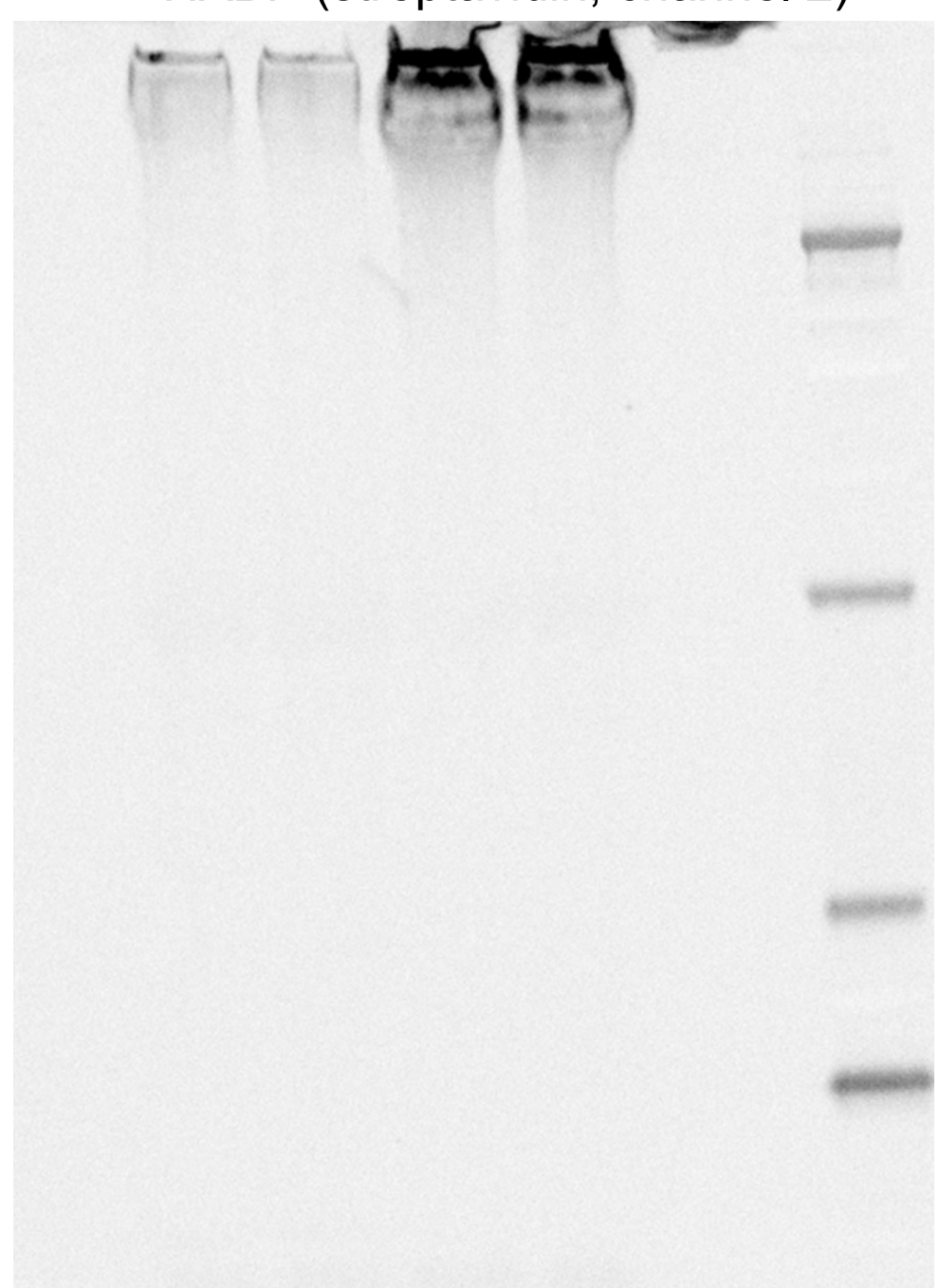

Chameleon Duo Pre-stained Protein Ladder

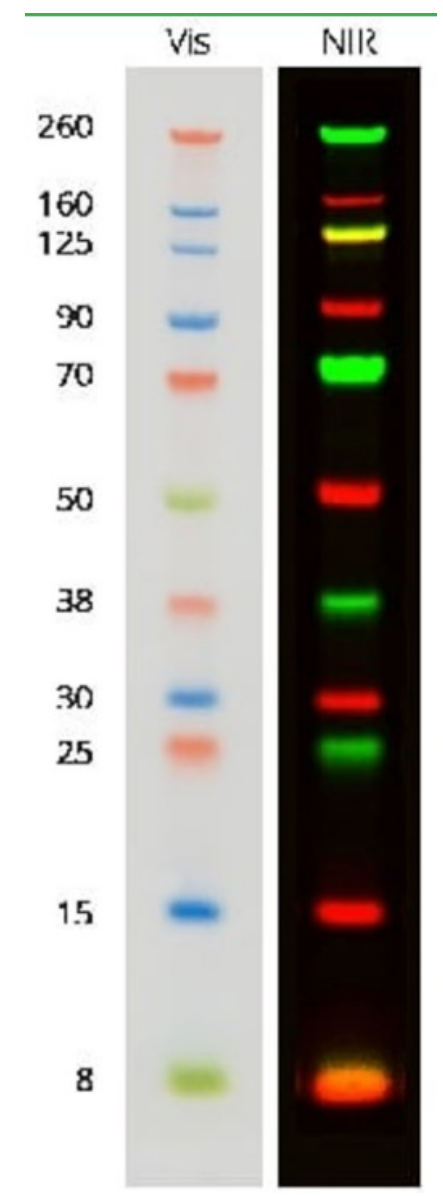

Corresponding Ponceau Staining – Pull down extraction

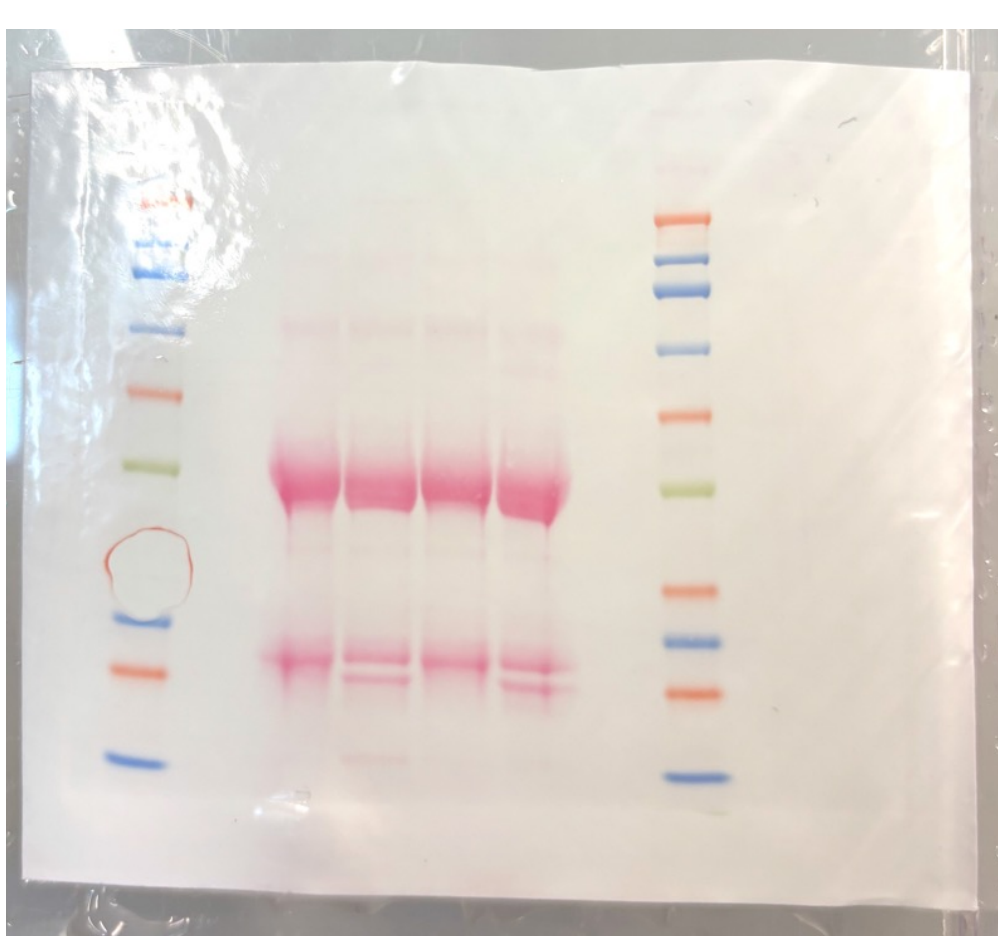

Corresponding Ponceau Staining – Supernatant extraction

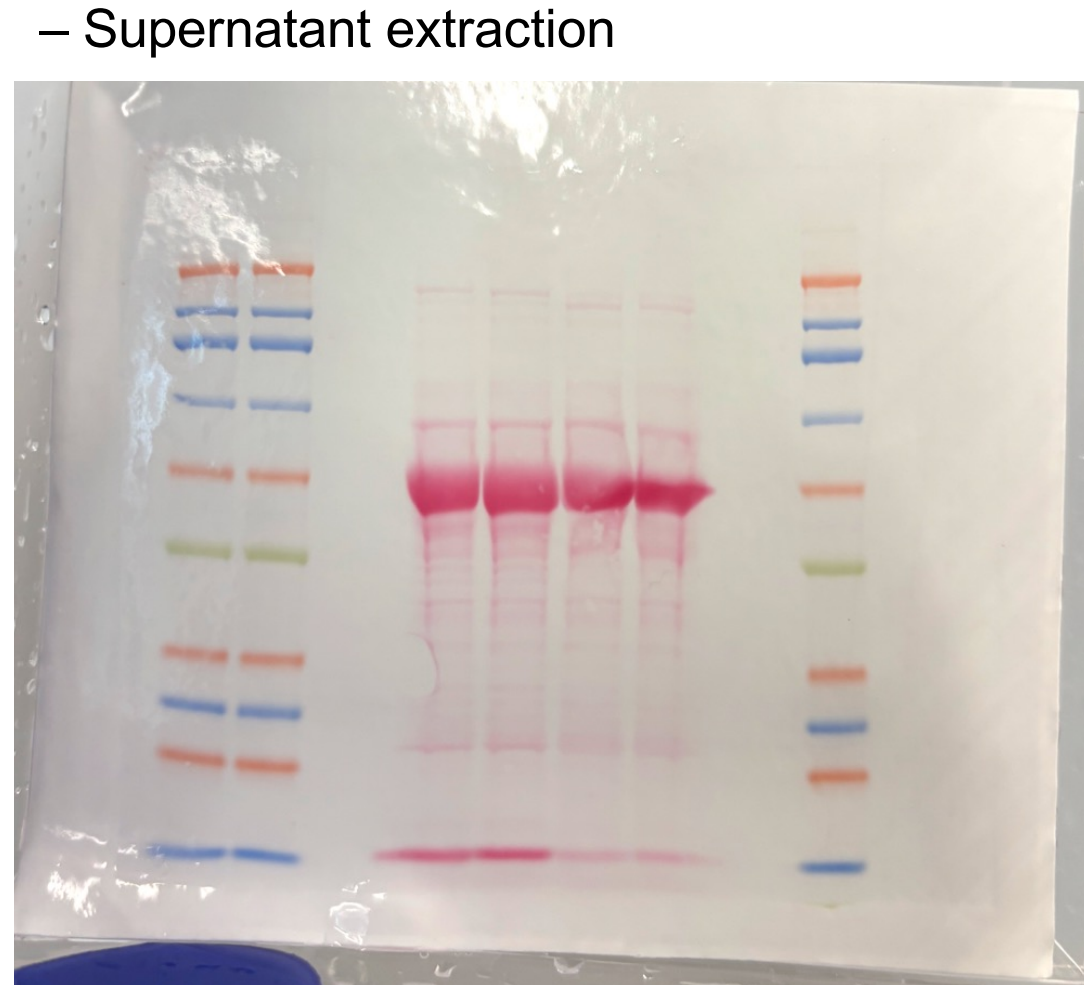

Full Western blot for Figure S3B

Novex™ Sharp Pre-Stained Protein Standard

Versican DPEAAE

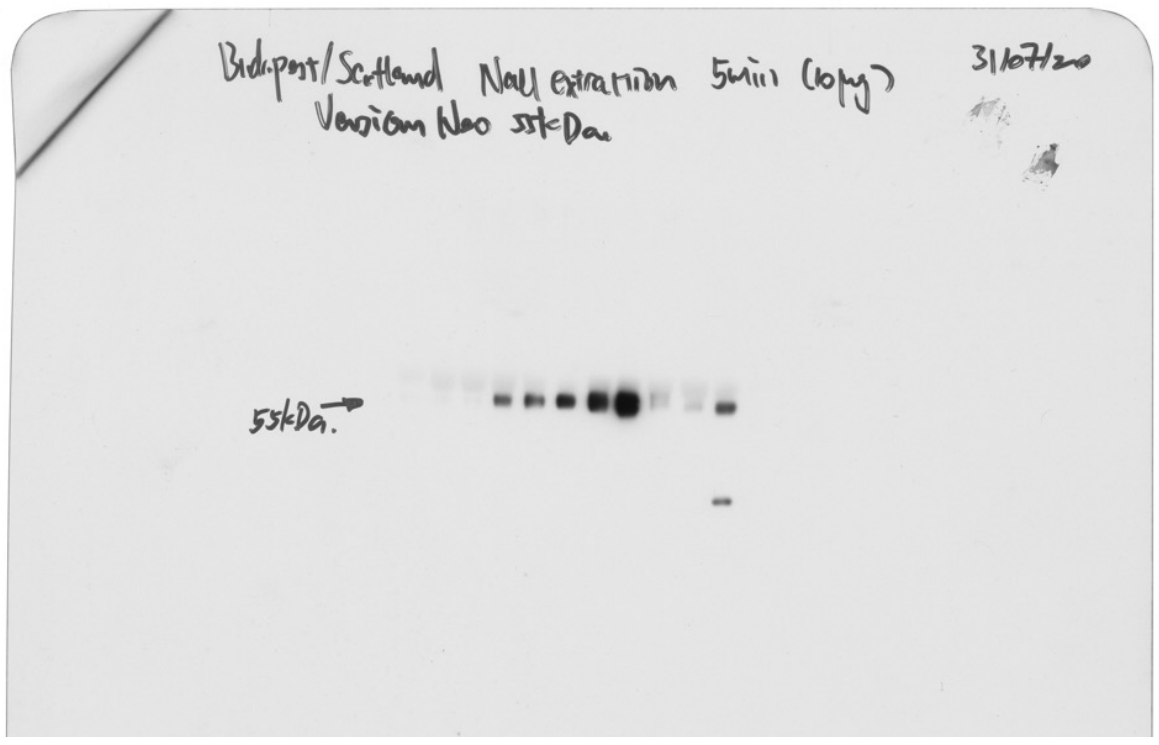

Corresponding Ponceau Staining

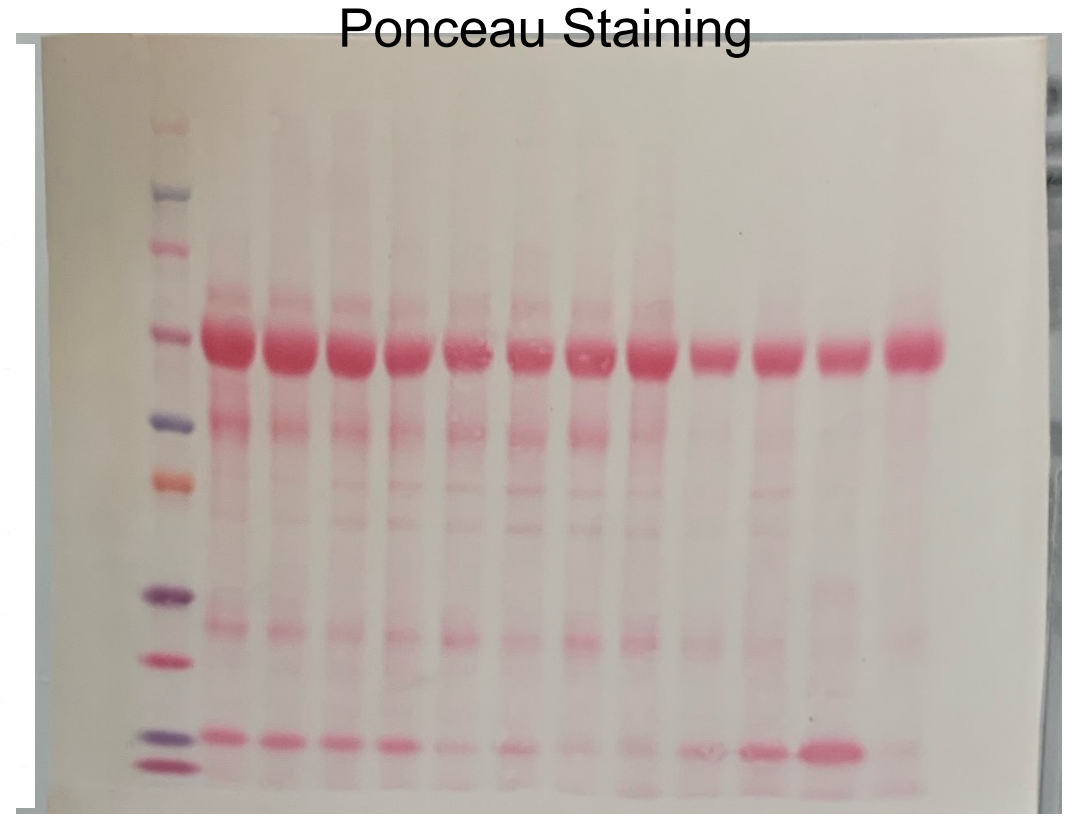

Chameleon Duo Pre-stained Protein Ladder

Aggrecan NITEGE

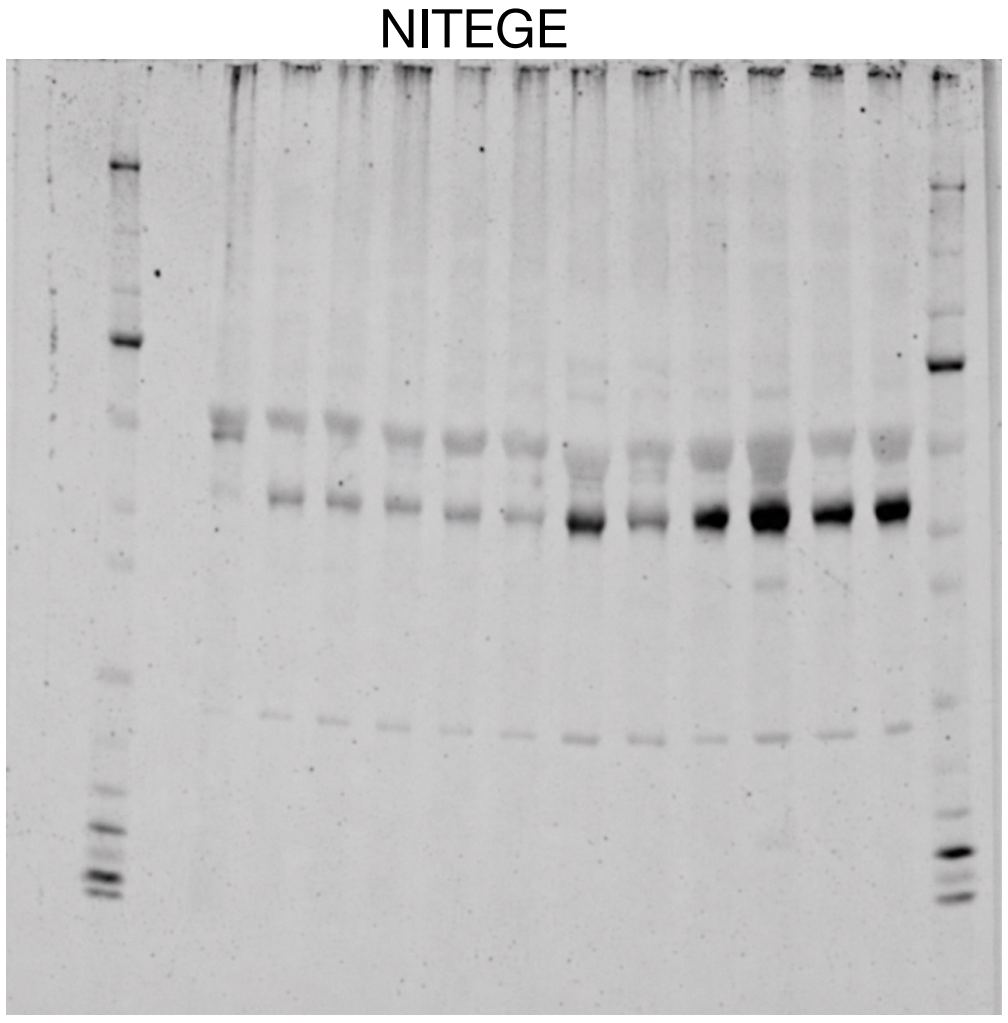

Corresponding Ponceau Staining

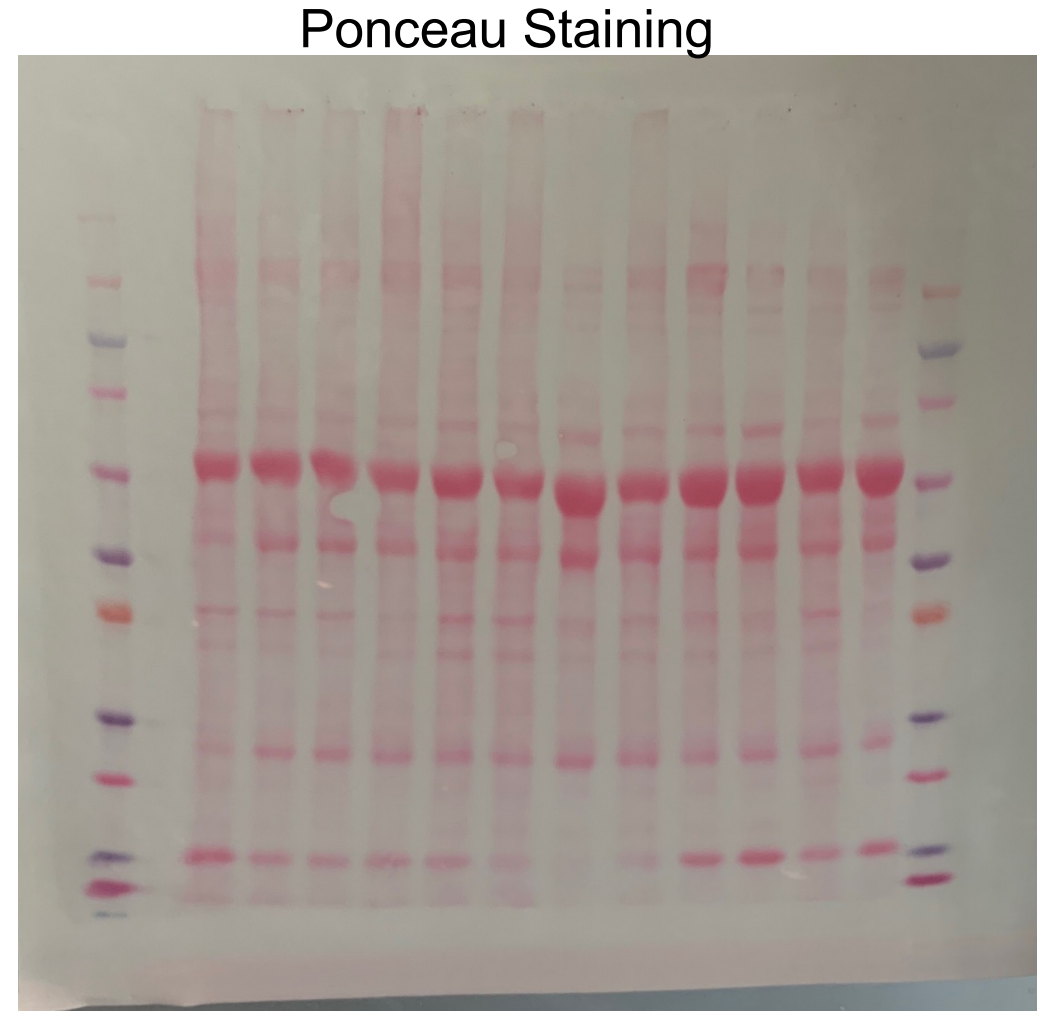

For VCAN core and ACAN core, due to its high molecular weight, it was overrun and HiMark™ Pre-stained Protein Standard was used

Aggrecan core

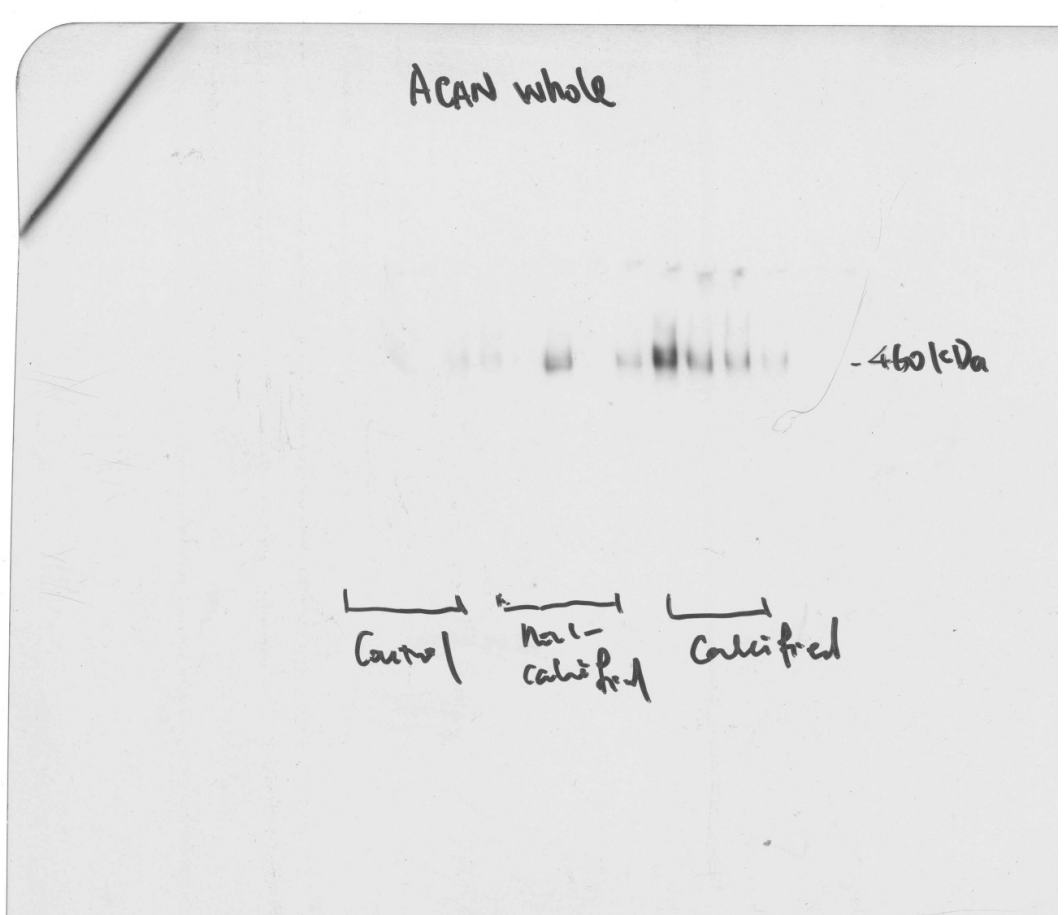

Corresponding Ponceau Staining

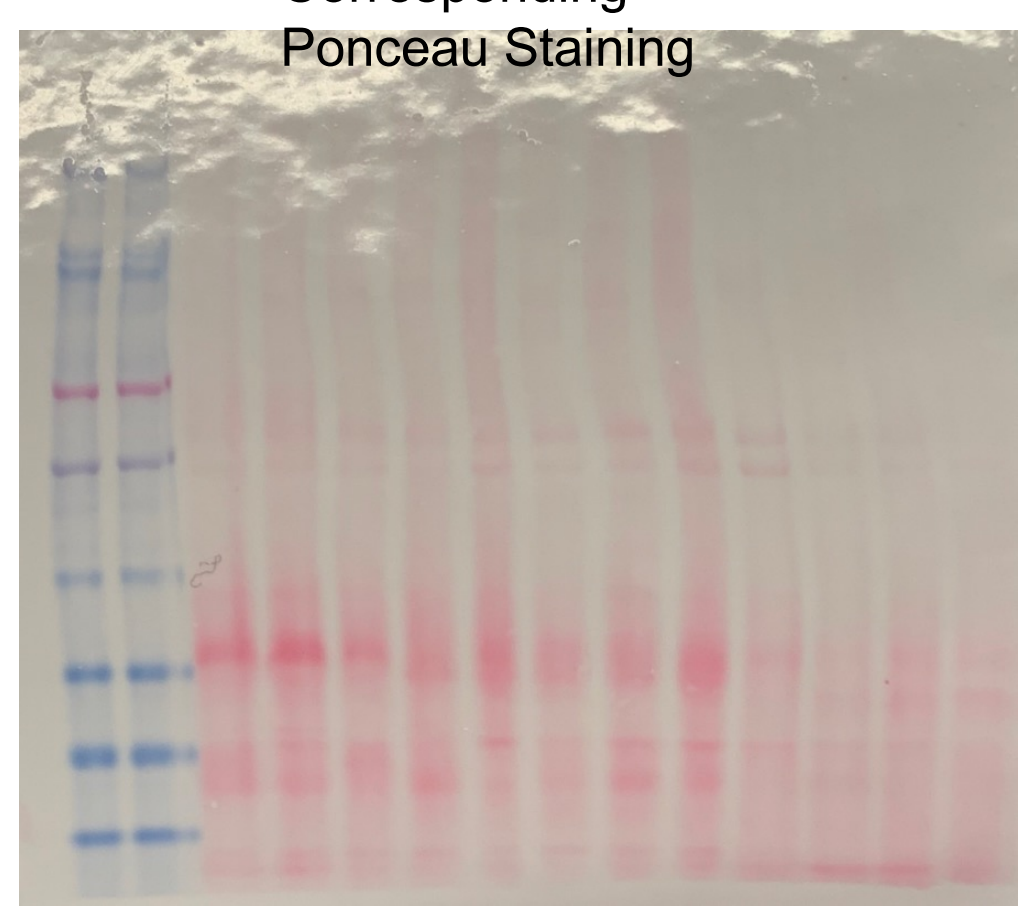

Versican core

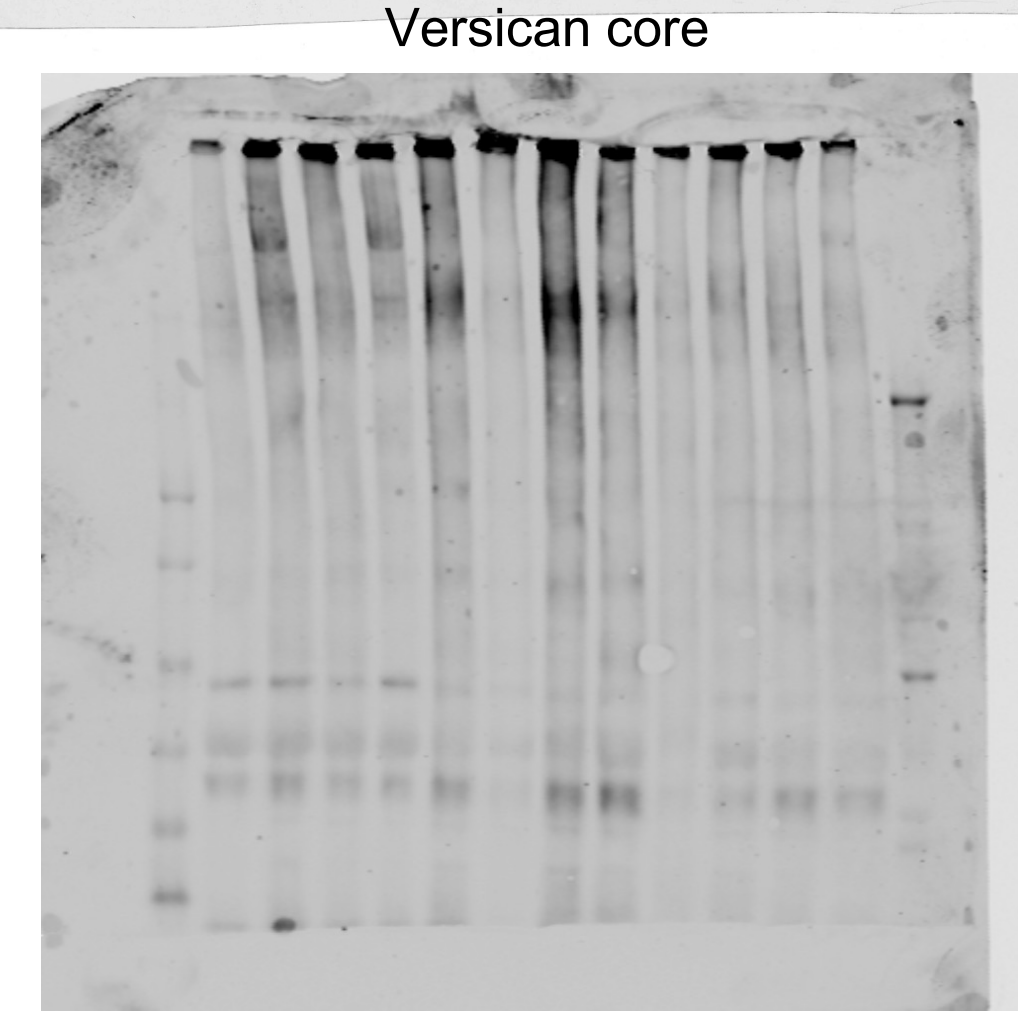

Corresponding Ponceau Staining

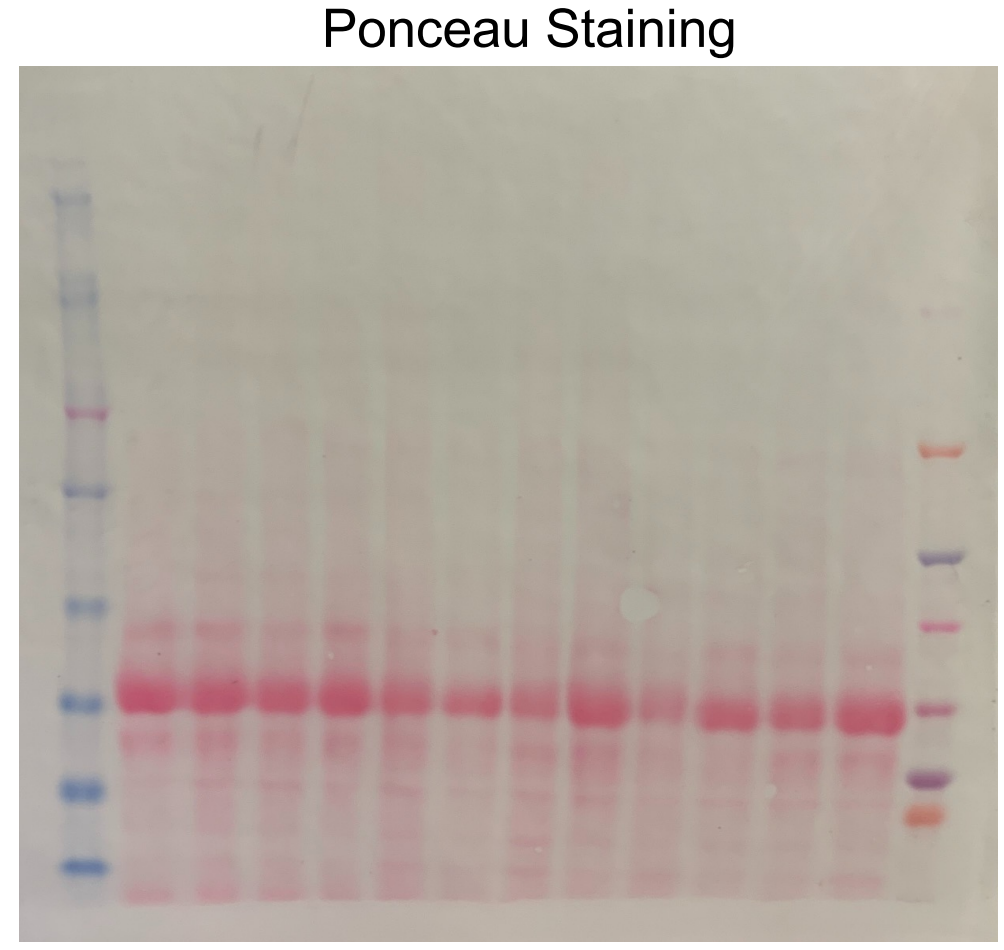

Full unedited Western blot for Figure S8E

Versican DPEAAE

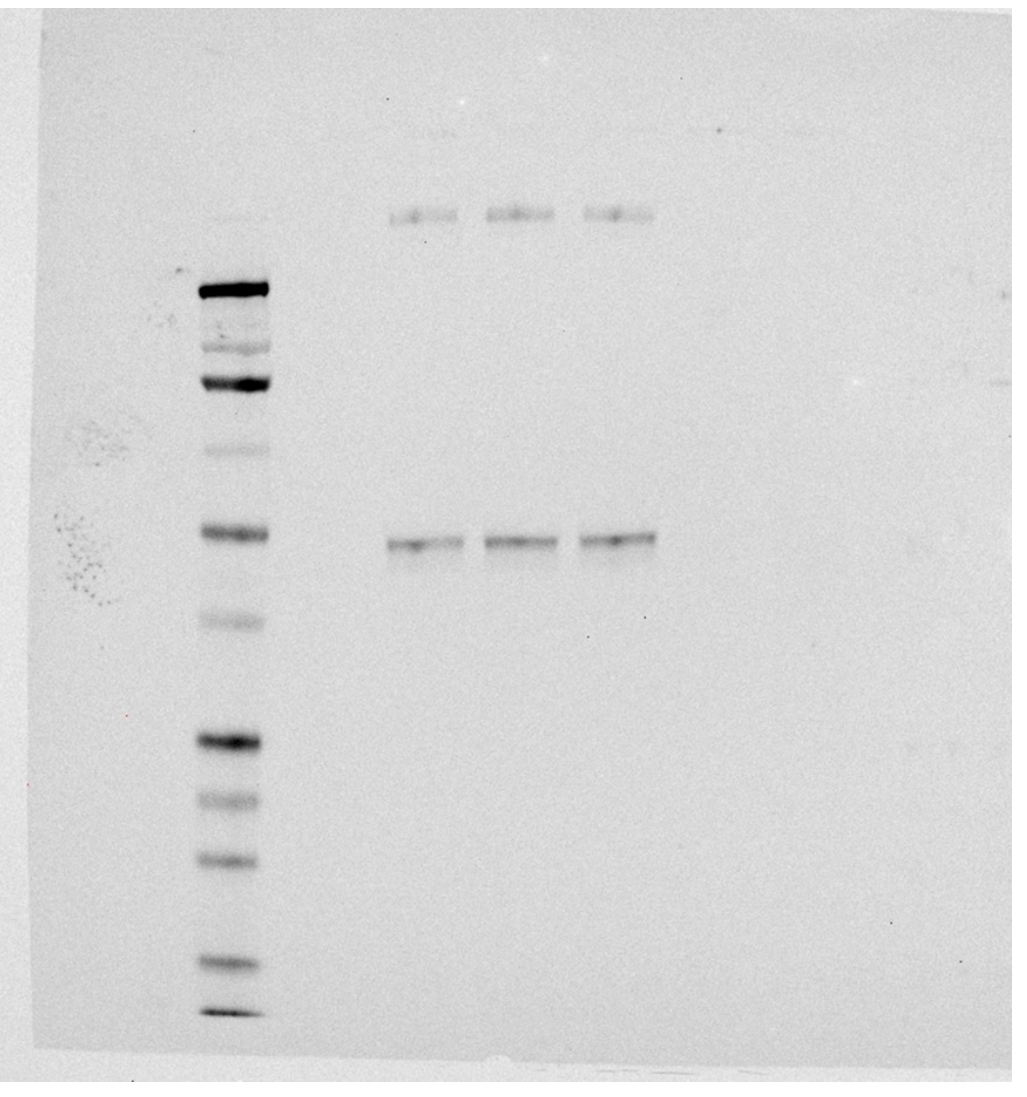

Versican Core

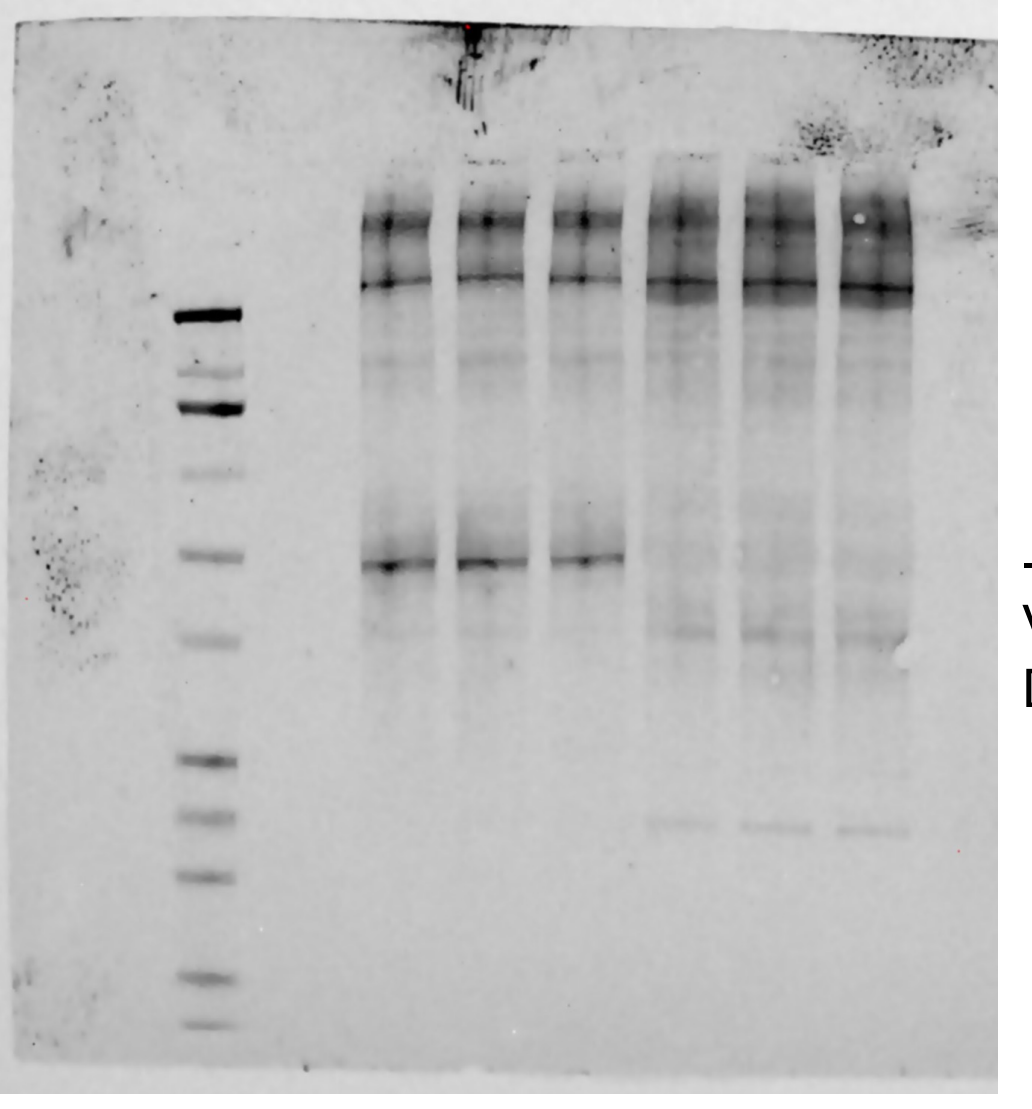

- Previous Versican DPEAAE band

Corresponding Ponceau Staining

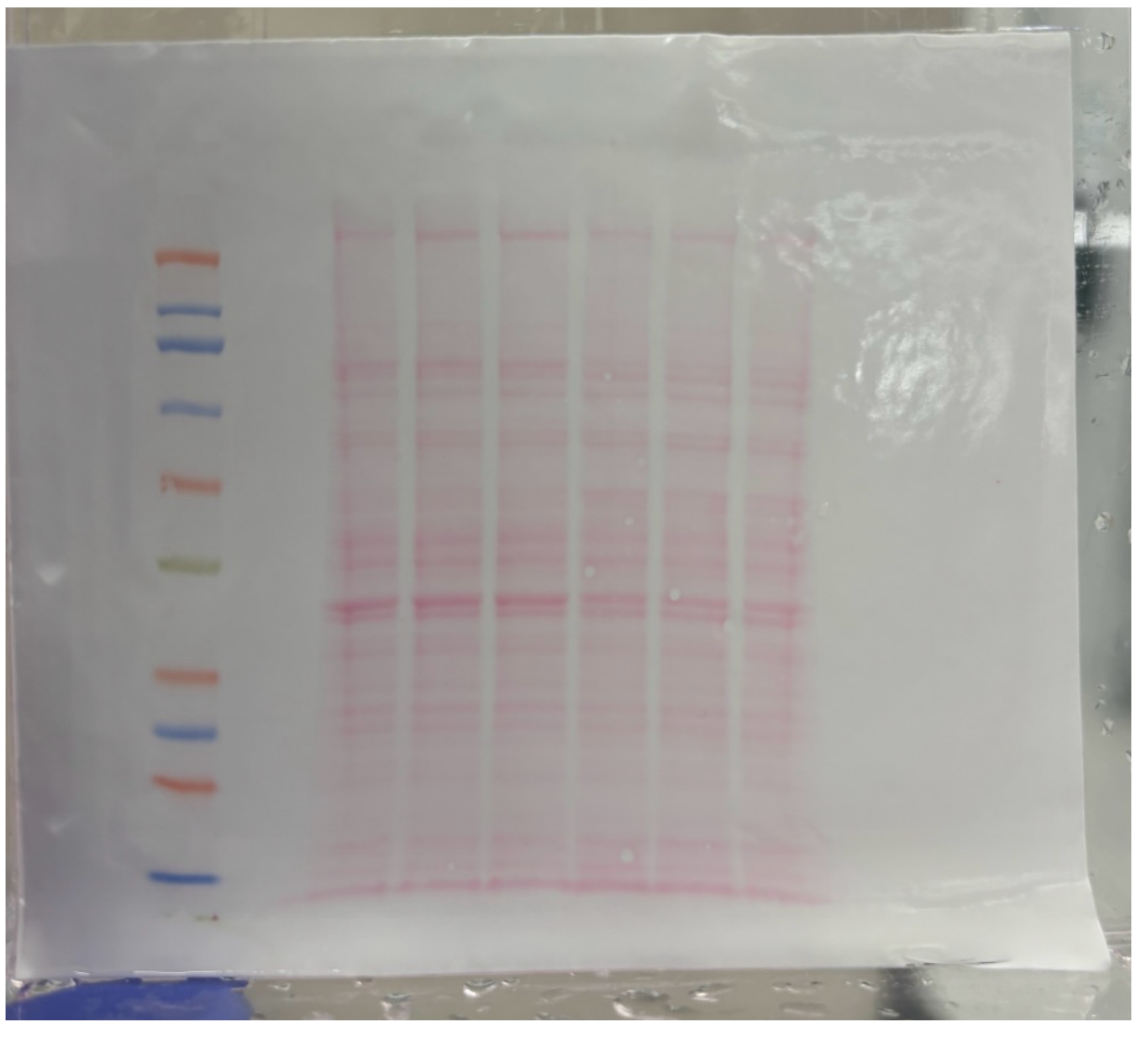

Chameleon Duo Pre-stained Protein Ladder

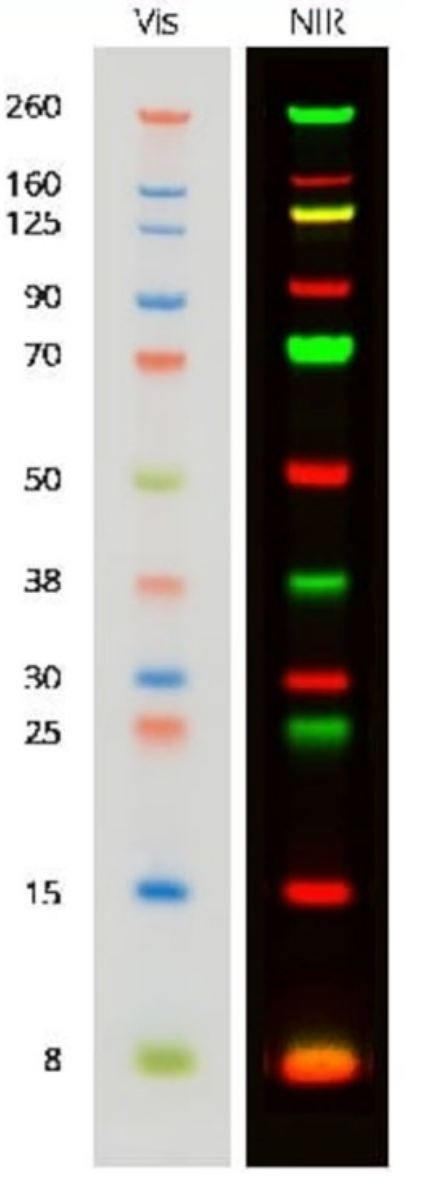

Full unedited Western blot for Figure S9D

Supernatant – nonreduced

PTX3

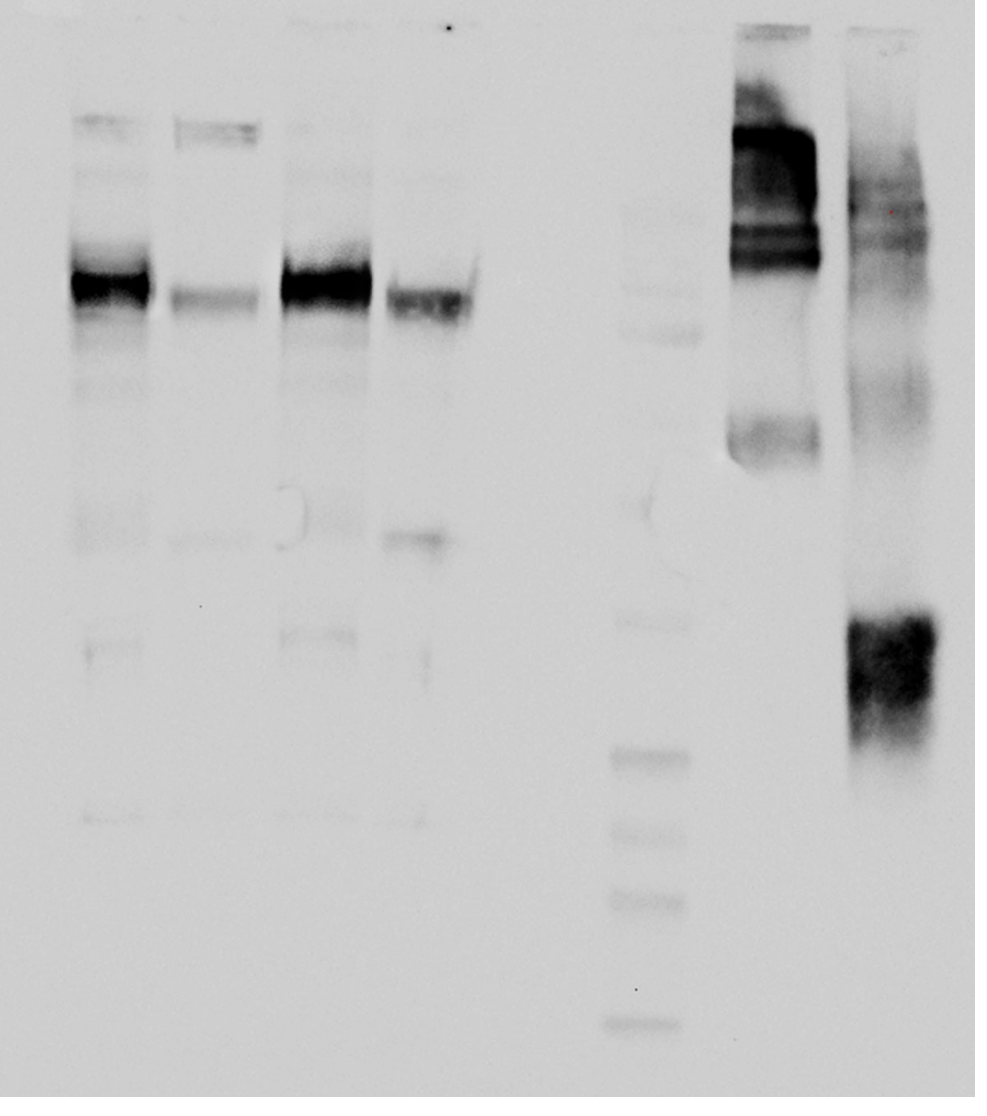

Corresponding Ponceau Staining

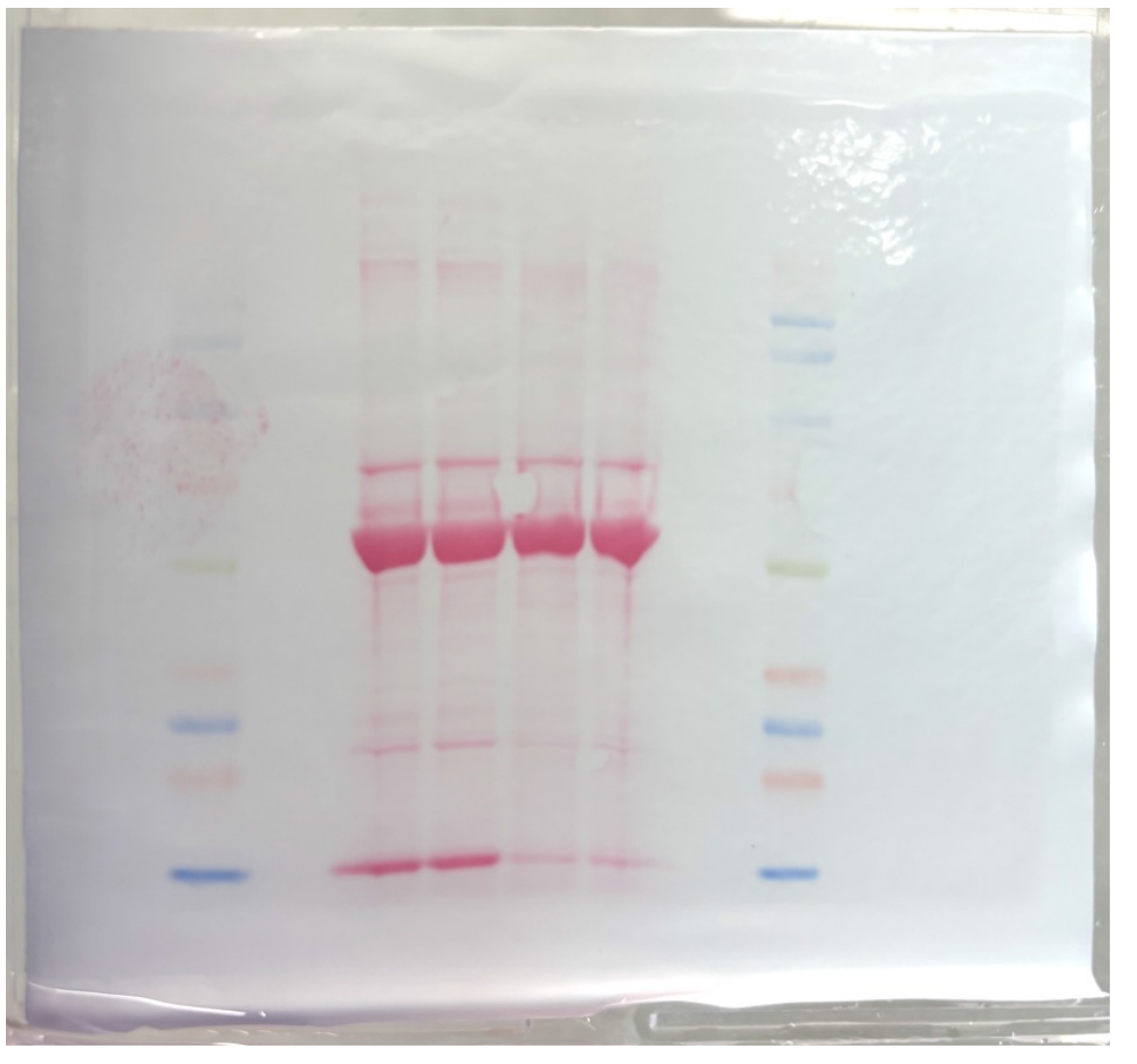

Chameleon Duo
Pre-stained
Protein Ladder

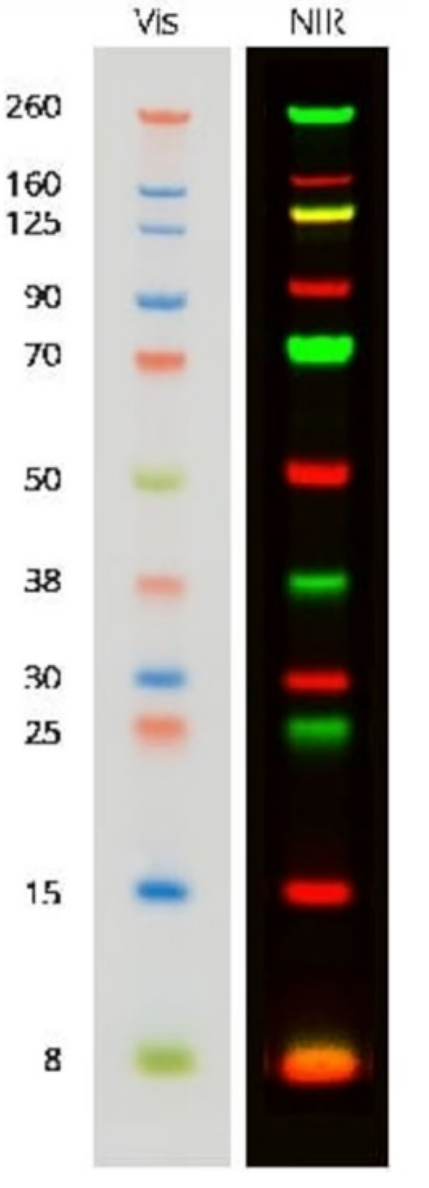

Supplement: Supplementary file 3 [file atv-46-e323867-s003.pdf]
